# Moonlighting glyceraldehyde-3-phosphate dehydrogenase (GAPDH) protein of *Lactobacillus gasseri* attenuates allergic asthma via immunometabolic change in macrophages

**DOI:** 10.1186/s12929-022-00861-8

**Published:** 2022-09-29

**Authors:** Pei-Chi Chen, Miao-Hsi Hsieh, Wen-Shuo Kuo, Lawrence Shih-Hsin Wu, Hui-Fang Kao, Li-Fan Liu, Zhi-Gang Liu, Wen-Yih Jeng, Jiu-Yao Wang

**Affiliations:** 1 Present Address: Center for Allergy, Immunology, and Microbiome (A.I.M.), China Medical University Hospital, No. 2, Yuh-Der Road, Taichung City, 404 Taiwan; 2 Department of Nursing, National Tainan Junior College of Nursing, Tainan, Taiwan; 3 School of Chemistry and Materials Science, Nanjing University of Information Science and Technology, Nanjing, China; 4 Graduate Institute of Biomedical Sciences, China Medical University, Taichung, Taiwan; 5 Institute of Gerontology, College of Medicine, National Cheng Kung University, Tainan, Taiwan; 6 Department of Respirology and Allergy, Third Affiliated Hospital of Shengzhen University, Shengzhen, China; 7 University Center for Bioscience and Biotechnology, National Cheng Kung University, Tainan, Taiwan; 8 Present Address: Department of Biochemistry and Molecular Biology, National Cheng Kung University, No. 1, University Road, Tainan City, 701 Taiwan; 9 Children’s Hospital, China Medical University, Taichung, Taiwan

**Keywords:** Probiotics, Allergic asthma, Moonlighting protein, Macrophages, GAPDH

## Abstract

**Background:**

The extra-intestinal effects of probiotics for preventing allergic diseases are well known. However, the probiotic components that interact with host target molecules and have a beneficial effect on allergic asthma remain unknown. *Lactobacillus gasseri* attenuates allergic airway inflammation through the activation of peroxisome proliferator- activated receptor γ (PPARγ) in dendritic cells. Therefore, we aimed to isolate and investigate the immunomodulatory effect of the PPARγ activation component from *L. gasseri*.

**Methods:**

Culture supernatants of *L. gasseri* were fractionated and screened for the active component for allergic asthma. The isolated component was subjected to in vitro functional assays and then cloned. The crystal structure of this component protein was determined using X-ray crystallography. Intrarectal inoculation of the active component-overexpressing *Clear coli* (lipopolysaccharide-free *Escherichia coli*) and intraperitoneal injection of recombinant component protein were used in a house dust mite (HDM)-induced allergic asthma mouse model to investigate the protective effect. Recombinant mutant component proteins were assayed, and their structures were superimposed to identify the detailed mechanism of alleviating allergic inflammation.

**Results:**

A moonlighting protein, glycolytic glyceraldehyde 3-phosphate dehydrogenase (GAPDH), LGp40, that has multifunctional effects was purified from cultured *L. gasseri*, and the crystal structure was determined. Both intrarectal inoculation of LGp40-overexpressing *Clear coli* and intraperitoneal administration of recombinant LGp40 protein attenuated allergic inflammation in a mouse model of allergic asthma. However, CDp40, GAPDH isolated from *Clostridium difficile* did not possess this anti-asthma effect. LGp40 redirected allergic M2 macrophages toward the M1 phenotype and impeded M2-prompted Th2 cell activation through glycolytic activity that induced immunometabolic changes. Recombinant mutant LGp40, without enzyme activity, showed no protective effect against HDM-induced airway inflammation.

**Conclusions:**

We found a novel mechanism of moonlighting LGp40 in the reversal of M2-prompted Th2 cell activation through glycolytic activity, which has an important immunoregulatory role in preventing allergic asthma. Our results provide a new strategy for probiotics application in alleviating allergic asthma.

**Supplementary Information:**

The online version contains supplementary material available at 10.1186/s12929-022-00861-8.

## Background

Allergic asthma is a mucosal inflammatory lung disease caused by a complex interaction involving allergen exposure, inflammatory cells, and microbiota in the airway mucosal epithelium. The bronchial mucosa forms the first line of defense of our body’s immune system, and thus comes into contact with not only allergens but also numerous commensal microbiota. Upon allergen exposure, macrophages quickly process and present the allergen to lymphocytes, thereby linking the group 2 innate lymphoid cells (ILC2) and type 2 helper T cells (Th2) responses [1, 2]. In this Th2-inflammatory microenvironment, macrophages transition to M2 (or alternatively activated phenotypes) and recruit eosinophils, mast cells, and Th2 cells to the lungs, which exacerbates the lung inflammation in allergic asthma [3, 4].

Probiotics establish immune homeostasis by increasing the regulatory T cell (Tregs) population and inducing their activation, thus preventing the development of allergen-driven inflammation [5]. We previously reported that oral intake of *Lactobacillus gasseri* improved allergic symptoms in children with asthma in a double-blind, randomized controlled trial [6]. In addition, experimental mouse models of asthma also revealed that oral *L. gasseri* administration attenuated allergen-induced airway inflammation and suppressed Th2 and Th17 immune responses [7]. Moreover, *L. gasseri* might act via a peroxisome proliferator-activated receptor-γ (PPARγ) activation pathway in dendritic cells to alleviate allergen-induced airway inflammation in asthma [8]. Additionally, Kelly et al. suggested that *Bacteroides thetaiotaomicron*, a commensal gut microflora, maintains immune homeostasis through a PPARγ-dependent anti-inflammatory mechanism [9]. However, clarity on which probiotic components interact with host target molecules and the immunomodulatory effects of the active components in allergic diseases remain unelucidated.

Bacterial glyceraldehyde-3-phosphate dehydrogenase (GAPDH) is a crucial glycolytic enzyme and has several additional functions beyond metabolism, which include adhesion to the host epithelium and modulation of the host immune response [10]. These multifunctional proteins, also known as moonlighting proteins, perform multiple functions that are not attributable to gene fusions, splice variants, or different protein domains [10]. The diverse localization of GAPDH and its non-enzymatic interaction with host target molecules have been reported previously [11–13]. Surface-exposed GAPDH can aid pathogens to capture host plasminogen to enhance bacterial invasiveness. The GAPDH secreted by pathogens may function as a virulence-associated immunomodulatory antigen to induce both innate and adaptive immunity [11]. Similarly, probiotic GAPDH localizes to the bacterial surface and adheres to host intestinal mucosa by binding to fibronectin or plasminogen in the host epithelium [12, 13]. Although few reports have suggested that probiotic secreted GAPDH is an immunomodulator, the anti-inflammatory effects of probiotics in allergic disease are well reported [14]. Therefore, it is rational to propose that probiotics may interact with host plasminogen through GAPDH to induce immunoregulatory effects in allergic diseases.

Here, we extend our previous studies of the immunomodulatory mechanism of *L. gasseri* on allergen-induced airway inflammation. We identified and purified a moonlighting protein, GAPDH, from *L. gasseri* (LGp40), which could induce PPARγ activation. We aimed to investigate the therapeutic potential and mechanism of LGp40 in house dust mite (HDM)-induced allergic asthma. Furthermore, we intended to explore the different immunomodulatory mechanisms between LGp40 and similar proteins from pathogens in allergic inflammation.

## Methods

### Identification of the anti-allergy fraction, IE3-3G1, from *L. gasseri*

*Lactobacillus gasseri* (BCRC14619) was cultured in Lactobacillus MRS broth (Merck Millipore, Burlington, MA, USA) at 37 °C, according to the ATCC guidelines. The pelleted bacteria were first dissolved in 1% lysozyme (Sigma-Aldrich, St. Louis, MO, USA) solution to lyse the cells. Ammonium sulfate (Sigma-Aldrich) was then added incrementally to the cytosolic fraction. The precipitated crude extracts were then separated through DEAE-Sepharose ion-exchange chromatography (HiTrap™ DEAE FF; GE Healthcare, Chicago, IL, USA) and further sub-separated through Sephacryl S-300 HR size-exclusion chromatography (Amersham Biosciences, Buckinghamshire, UK). The crude extracts, fractions, and sub-fractions were subsequently co-cultured with mouse bone marrow-derived dendritic cells (BMDC) to screen the active ingredient (detailed information is provided in Additional file 1).

### Measurement of interleukin (IL)-12p40 from cultured supernatant of BMDC

For BMDC differentiation, the femurs and tibias were isolated from 6-to 8-week-old BALB/c mice and the muscle tissue was removed. The bone marrow was flushed out with RPMI 1640 (Corning, Glendale, AZ, USA) using a 25-gauge needle. After lysis of erythrocytes, cells were seeded at a density of 5 × 10^5^/mL in RPMI 1640 containing 10% fetal bovine serum (FBS; Corning) and 20 ng/mL granulocyte–macrophage colony-stimulating factor (GM-CSF; PeproTech, Cranbury, NJ, USA). After 8-day culture, suspended BMDC were harvested. Differentiated cells were seeded at a density of 1 × 10^6^ cells/mL and were stimulated with 10 μg/mL lipopolysaccharide (LPS; Sigma-Aldrich), 30 μg/mL phytohemagglutinin (PHA; Sigma-Aldrich), or 10 μg/mL of crude extracts, fractions, and sub-fractions for 48 h. IL-12p40 levels were measured in the cultured supernatant using an enzyme-linked immunosorbent assay (ELISA) assay kit (R&D Systems, Minneapolis, MN, USA).

### Measurement of PPARγ from BMDC cell lysate

Differentiated BMDC from 6-to 8-week-old C57BL/6 J mice were seeded at a density of 1 × 10^6^ cells/mL and were stimulated with 5 μg/mL LPS, 5 μM rosiglitazone (Ro; Sigma-Aldrich), or 1, 10, and 25 μg/mL sub-fraction IE3-3G1 for 24 h. After washing with PBS (phosphate buffered saline), whole-cell lysates were prepared by using radioimmunoprecipitation assay (RIPA) buffer (Sigma-Aldrich) and were then boiled in sample buffer (62.5 mM Tris–Cl [pH 6.8], 2% sodium dodecyl sulfate [SDS], 20% glycerol, 10% 2-mercaptoethanol) at 99 ℃ for 15 min. Samples were electrophoresed on 10% SDS–polyacrylamide gel electrophoresis (PAGE) and transferred onto polyvinylidene difluoride (PVDF) membranes (Merck Millipore) for Western blotting. The membranes were blocked for 1 h with 10% non-fat milk in TBST (150 mM NaCl, 50 mM Tris–Cl [pH 7.4], 0.05% Tween 20), and incubated overnight with PPARγ (Cayman Chemical, Ann Arbor, MI, USA) and β-actin (GeneTex, Irvine, CA, USA) antibodies. The membranes were then incubated with horseradish peroxidase (HRP)-conjugated secondary antibodies (GeneTex) for 2 h, further treated with the Western lightning chemiluminescence reagent (PerkinElmer, Waltham, MA, USA), and visualized with X-ray film. The density of detected proteins was calculated in comparison with the density of β-actin, and the results are reported as the percentage.

### Protein identification of sub-fraction IE3-3G1 by two-dimensional gel electrophoresis (2DE) and liquid chromatography-tandem mass spectrometry (LC–MS/MS)

The protein identification was generated by Proteomics Research Core Laboratory (Medical College, National Cheng Kung University, Tainan, Taiwan) using modified versions of methods that were described previously [15, 16]; 120 μg sub-fraction IE3-3G1 was precipitated with 10% w/v trichloroacetic acid in acetone, and precipitates were washed with acetone containing 20 mM dithiothreitol three times and then dissolved in isoelectric focusing (IEF) rehydration buffer for 2DE analysis that was performed using IEF as the first dimension. The IPGphor system (GE Healthcare) and immobiline DryStrip gels (11 cm, GE Healthcare) with a pH gradient of 4–10 were used. The strips were then transferred onto the second-dimensional 10% SDS-PAGE (Bio-Rad protean II xi cell, Hercules, CA, USA). The gels were fixed and stained in 0.25% w/v silver nitrate solution containing 0.02% formaldehyde. Selected protein spots were excised and then digested with trypsin (Promega, Madison, WI, USA) to generate the constituent peptides for subsequent two-dimensional nano-high performance liquid chromatography and electrospray ionization tandem mass spectrometry (2D nano-HPLC–ESI–MS/MS) analysis. The protein tryptic digests were fractionated using a nano-HPLC system (LC Packings, Amsterdam, The Netherlands) coupled to an ion-trap mass spectrometer (LCQ DECA XP Plus, ThermoFinnigan, San Jose, CA, USA) that was equipped with an electrospray ionization source. Tandem mass spectrometry data were analyzed by the database search software Matrix Science (Boston, MA, USA) with MASCOT tools to interpret the MS/MS spectra.

### Overexpression and purification of His6-tagged recombinant GAPDH proteins

GAPDH from *L. gasseri* (BCRC14619), *L. reuteri* (JCM1112), and *Clostridium difficile* (CD630DERM) were cloned into pET21b vectors (addgene, Watertown, MA, USA) using a restriction enzyme-cloning method [17]. The loss-of-function protein, LGp40-C156S and CDp40-C155S, were cloned using the FastCloning method [18]. All prepared plasmids were transformed into *Escherichia coli* BL21 (DE3). Recombinant His-tagged LGp40, LRp40, CDp40, LGp40-C156S, and CDp40-C155S proteins were induced by isopropyl-β-d-thiogalactopyranoside (IPTG; Sigma-Aldrich). Harvested cells were disrupted by sonication. The supernatant was subsequently loaded into a Ni–NTA column (GE Healthcare) to purify recombinant proteins. These proteins were loaded into a Q column (GE Healthcare) to remove LPS and then concentrated for further use. The concentrated LGp40 protein and sub-fraction IE3-3G1 were subjected to electrophoresis on a 10% SDS-PAGE gel for Coomassie blue staining and Western blot analysis of the antibody to His-tag (GeneTex). Additionally, pET21b-LGp40-His plasmid DNA was transformed into *Clear coli* (LPS free *E. coli*) and LGp40 were induced by IPTG for further animal model (Protocol 1) use. To determine the protein concentrations of LGp40 in IPTG non-induced and IPTG-induced *Clear coli*, the same amounts of bacteria (OD_600_ = 1) were lysed. The supernatant was subjected to electrophoresis on 10% SDS-PAGE gel.

### Crystallization

Purified LGp40 protein was prepared to obtain a concentration of 8–10 mg/mL in protein buffer to enable crystallization. Initial crystallization screening was carried out by a protein crystallization robot Mosquito Crystal (SPT Labtech, Hertfordshire, UK) by using the hanging-drop vapor diffusion method. LGp40 crystals were observed in the hanging-drops containing 50 nL of protein solution and 50 nL of various reservoir solutions at 25 °C within 1 week. Diffraction-quality crystals were obtained using the sitting-drop method by mixing 1 µL each of protein and reservoir solutions (0.1 M sodium cacodylate [pH 6.5], and 16% w/v PEG 4000) for further manual optimization of crystallization. The CombiClover Junior sitting plate (Emerald BioSystems, Bainbridge Island, WA, USA) and 200 μL of reservoir solution was used per well for manual crystallization.

### Diffraction data collection, structure determination, and refinement

Crystals were soaked briefly in a cryoprotectant solution containing 16% w/v PEG 4000 and 20% ethylene glycol in 0.1 M sodium cacodylate buffer (pH 6.5). Then, all crystals were flash-cooled to 100 K in a stream of cold nitrogen prior to data collection. X-ray diffraction data were collected on SPXF beamline BL13C1 at the National Synchrotron Radiation Research Center in Taiwan. The diffraction images were processed using the HKL2000 program package (HLK Research, Inc., Charlottesville, VA, USA) [19]. Structures were determined by molecular replacement with Phaser [20] using the deposited structure of GAPDH1 from methicillin-resistant *Staphylococcus aureus* MRSA252 (Protein Data Bank, PDB, code: 3LVF) as a search model [21]. Automatic model building was performed with Buccaneer [22]. Model completion and refinement were performed with REFMAC5 [23, 24] and COOT [25]. A 5% subset of randomly selected reflections was excluded from computational refinement to calculate the Rfree factor throughout the refinement [26]. All final refinements were carried out using REFMAC5 with the TLS group tensor and anisotropic B factor. The stereochemistry and structure of the final models were analyzed by RAMPAGE [27] and SFCHECK [28] of the CCP4 program suite [29, 30]. The structural figures were produced using PyMOL (DeLano Scientific, South San Francisco, CA, USA, http://www.pymol.org).

### Phylogenetic studies

The phylogenetic tree was based on the GAPDH protein sequences acquired from the KEGG pathway database (Kyoto Encyclopedia of Genes and Genomes, https://www.genome.jp/kegg/) under the following accession numbers: LGAS_1308 (*L. gasseri* BCRC14619/ATCC33323 GAPDH), LAR_0381 (*L. reuteri* JCM1112 GAPDH), lp_0789 (*L. plantarum* WCFS1 GAPDH), BL1363 (*Bifidobacterium longum* NCC2705 GAPDH), CPF_1511 (*C. perfringens* ATCC13124 GAPDH), CD630_17670 (*C. difficile 630* GAPDH), SL1344_1225 (*Salmonella enterica* SL1344 GAPDH), and ECs2488 (*E. coli* O157H7 GAPDH). The phylogenetic tree was generated using Phylogenetic Tree Prediction (GeneBee service, Moscow State University, Moscow, Russia) and then visualized using Phylodendron (D.G. Gilbert, Indiana University, Bloomington, IN, USA).

### Enzymatic activity assay

The enzymatic activity of recombinant GAPDH was determined by assaying the rate of NADH oxidation using the ScienCell™ Colorimetrtic GAPDH Assay kit (ScienCell, Carlsbad, CA, USA).

### Animal experimental models and protocols

We purchased 6-to 8-week-old female BALB/c mice from National Applied Research Laboratories, Taipei, Taiwan. Mice were bred and housed under specific pathogen-free conditions in the Laboratory Animal Center of the National Cheng Kung University and in accordance with national animal guidelines and regulations. The HDM allergen (*Dermatophagoides pteronyssinus*; Allergon AB, Ängelholm, Sweden) was dissolved in pyogenic-free isotonic saline, filtered through a 0.22-μm filter, and stored at − 80 °C until use.

#### Protocol 1

To identify the protective effect of LGp40, mice were orally supplied with a cocktail of broad-spectrum antibiotics for 2 weeks. The antibiotic cocktail consisted of ampicillin (1 g/L; Cyrusbioscience, Taipei, Taiwan), vancomycin (0.5 g/L; Centaur Services Ltd., Castle Cary, UK), neomycin (0.5 g/L; Cyrusbioscience), and metronidazole (1 g/L; Cyrusbioscience). For a disease experimental control, the SC group (mice without allergic asthma) was sensitized and challenged with PBS. Allergic asthma was included in the other mice (including the AS, NC, IC, and LG groups) by intraperitoneal sensitization with a mixture of 50 μg HDM and 1 mg aluminum hydroxide (ThermoFisher, Waltham, MA, USA) on days 0 and 7. The mice were further anesthetized and intranasally challenged with 50 μg HDM from days 7 to 11 and intratracheally challenged with 50 μg HDM on Day 14. Furthermore, each group was intrarectally administered PBS (SC and AS groups), 10^8^ LGp40 non-induced *Clear coli* (NC group), 10^8^ LGp40-induced *Clear coli* (IC group), or 10^8^ *L. gasseri* (LG group) daily during the HDM sensitization period. Airway hyperresponsiveness in mice was measured and animals were sacrificed 48 h after the last HDM exposure.

#### Protocol 2

To identify the different immunomodulatory effects of LGp40 and CDp40, all mice, except those in the SC group, were intraperitoneally sensitized with a mixture of 50 μg HDM and 1 mg aluminum hydroxide on days 0 and 7. Thereafter, the mice were intratracheally challenged with 50 μg HDM on days 11 and 21, followed by intranasal challenge with 25 μg HDM from days 14 to 18. In addition, selected groups were intraperitoneally injected with PBS (AS group), 10 μg LGp40 (Low-LGp40 group), 100 μg LGp40 (High-LGp40 group), or 10 μg CDp40 (CDp40 group), on days 1, 13, and 20. The SB group was orally administered 5 mg sodium butyrate (Sigma-Aldrich) daily during the HDM sensitization period. Mouse airway hyperresponsiveness was measured, and animals were sacrificed 48 h after the last HDM exposure. Lung single-cell suspensions and peritoneal macrophages were harvested for further flow cytometry analysis.

#### Protocol 3

To determine the different immunomodulatory effects between LGp40 and CDp40 in relation to enzyme activity, HDM-induced AS mice were conducted akin to Protocol 2. Selected groups were intraperitoneally injected with 20 μg of recombinant GAPDH proteins (LGp40 and CDp40) and loss-of-function proteins (LGp40-C156S and CDp40-C155S) during the HDM sensitization period.

### Measurement of total and HDM-specific IgE in sera

Sera were collected from mice on the second day after the last allergen challenge. Total IgE levels in sera were assayed using ELISA (Bethyl Laboratories, Montgomery, TX, USA). For HDM-specific IgE, 96-well plates were coated with 10 μg of HDM at 4 °C for 18 h. After blocking, diluted sera (1:5 dilution) were added and incubated overnight at 4 °C. The plates were incubated with HRP-conjugated goat anti-mouse IgE antibodies (Bethyl Laboratories). Finally, the absorbance of the samples was determined at 450 nm.

### Lung function measurements

Airway resistance (Rrs) and elastance (Ers) measurements were performed using the flexiVent FX system (SCIREQ, Montréal, Canada). Mice were anesthetized with an intraperitoneal injection of pentobarbital sodium (50–90 mg/kg body weight) and then tracheostomized for intratracheal microtube measurement. Airway hyperresponsiveness was determined in response to increasing doses of aerosolized methacholine (1–4 mg/mL; Sigma-Aldrich).

### Bronchoalveolar lavage fluid (BALF) collection and cell infiltration

BALF was obtained by inserting a catheter into the trachea, through which cold PBS (1 mL) was administered twice. Supernatants and cells were separated by centrifugation at 300×*g* for 5 min. The total number of cells in the two collections was counted with a hemocytometer. Differential cellular analysis was performed using cytocentrifugation. Cells were then stained with Liu’s Stain solution for microscopic examination, and 200 cells were counted. Levels of TARC (thymus- and activation-regulated chemokine) and CCL24 (C–C motif chemokine 24) in the BALF of experimental animals were measured using an ELISA assay kit (R&D Systems).

### Lung histology and immunohistochemistry (IHC)

The entire lung was removed and fixed in 3.7% neutral buffered formalin (pH 7.4; Sigma-Aldrich) for 2 days. Specimens were embedded in paraffin and sliced at a thickness of 4 μm. H&E (hematoxylin and eosin) staining was performed according to the manufacturers’ protocols (ScyTek Laboratoriea, Logan, UT, USA). Lung sections were deparaffinized for immunostaining. After antigen retrieval and non-specific binding blocking, the samples were incubated with anti-iNOS (anti-inducible nitric oxide synthase; GeneTex) and anti-arginase-1 antibodies (GeneTex). Immunoreactivity was visualized using the diaminobenzidine immunoperoxidase methodology with the Novolink™ Polymer Detection Systems kit (Leica, Wetzlar, Germany).

### Isolation of single-cell lung suspensions and peritoneal lavage cells from Protocol 2 mice

Lungs were minced and incubated in digestion media containing 0.7 mg/mL collagenase (Sigma-Aldrich), and 0.03 mg/mL deoxyribonuclease I (Sigma-Aldrich) for 90 min at 37 °C. The single-cell suspension was obtained by passing through 70-μm cell strainers (BD Bioscience, Franklin Lakes, NJ, USA). Peritoneal lavage cells were harvested by flushing the peritoneal cavity with cold sterile Hanks′ Balanced Salt solution (Sigma-Aldrich). If necessary, erythrocytes were lysed.

### Plasminogen–GAPDH interaction by solid-phase assays

One microgram of human plasminogen (BioVision, Milpitas, CA, USA) was used to coat polystyrene plates and increasing concentrations of LGp40, LGp40-C156S, CDp40, and CDp40-C155S recombinant proteins (3.9 to 250 ng) were added to the wells. In inhibition tests, increasing concentrations of EACA (ε-aminocaproic acid, 1.25 to 10 mM; Sigma-Aldrich) were included with 125 ng of recombinant GAPDH proteins. Detection of bound GAPDH was measured by the addition of anti-His tag antibodies followed by HRP-conjugated secondary antibodies. Absorbance at 450 nm was read using an ELISA reader (Spectramax Plus, Molecular Devices, San Jose, CA, USA).

### Plasminogen activation assay

Briefly, 20 μg of recombinant GAPDH proteins was incubated with 150 nM human plasminogen, 0.5 U urokinase-type plasminogen activator (uPA; Sigma-Aldrich), and 0.2 mM chromogenic substrate of plasmin (MyBioSource, San Diego, CA, USA). Increase in plasmin activity was assessed at 30-min intervals by measuring absorbance at 405 nm for 3 h. In inhibition tests, 10 mM EACA was added.

### Bone marrow-derived macrophage (BMDM) differentiation and naïve Th cells isolation

For BMDM differentiation, bone marrow from 6-to 8-week-old BALB/c mouse was flushed out followed the same procedure as BMDC differentiation without lysed erythrocytes. Cells were seeded at a density of 4 × 10^5^/mL in RPMI 1640 containing 10% FBS and 10 ng/mL of macrophage colony-stimulating factor (M-CSF; PeproTech). Cell culture medium was subsequently changed every 3 days and replaced with fresh complete medium. After 7 days of culture, adherent BMDM were harvested. Naïve Th cells were isolated from spleens of BALB/c mice using the Naive CD4^+^ T Cell Isolation Kit, an LS Column, and a MidiMACS™ Separator (Miltenyi Biotec, Bergisch Gladbach, Germany), according to the manufacturer’s instructions.

### In vitro BMDM experiment

The M1 phenotype was generated by 10 ng/mL LPS and 20 ng/mL interferon γ (IFN-γ; PeproTech), whereas M2 phenotype was generated by 20 ng/mL each IL-4 (PeproTech) and IL-13 (PeproTech) for 24 h. Next, IL-4- and IL-13-driven BMDM at a density of 3 × 10^5^/ml in 12-well plates were stimulated with 5 μg/mL LGp40, LGp40-C156S, CDp40, and CDp40-C155S at 37 °C for 48 h. Cultured supernatants were collected and the levels of IL-6, IL-12p40, and IL-10 were quantified using ELISA assay kits (R&D Systems). Lactate concentrations were determined using fluorometric quantification assay kits (Biovision).

### BMDM and naïve Th cell co-culture system

Twenty-four-well plates were pre-coated with anti-CD3/CD28 antibodies (BD Bioscience) in PBS at 4 °C for 24 h and washed once with PBS before cell plating. Differentiated BMDM were incubated with naïve Th cells in a 1:10 proportion (BMDM: naïve T cells = 5 × 10^4^: 5 × 10^5^). These cells were co-stimulated with 5 μg/mL LGp40, LGp40-C156S, CDp40, and CDp40-C155S and 20 μg/mL LGp40 and CDp40 at 37 °C for 3 days. Cells were then collected for flow cytometric analysis.

### Flow cytometric analysis

Surface markers expressed by macrophages were determined with the following antibodies: FITC-conjugated F4/80, PerCP-Cy5.5-conjugated CD45, APC-Cy7-conjugated Ly6c, and BV605-conjugated CD11b antibodies (ThermoFisher and BD Biosciences). Cells were fixed in PBS containing 1% paraformaldehyde (Sigma-Aldrich). Ten thousand gated events were analyzed using CytoFLEX S (Beckman Coulter, Brea, CA, USA). M1 and M2 cells in AS mice were identified as F4/80^low^CD11b^low^Ly6c^hi^ (M1) and F4/80^hi^CD11b^hi^Ly6c^low^ (M2) using the same gating strategy that has been described previously [31].

### RNA-sequencing (RNA-seq) analysis

BMDM from 6-to 8-week-old C57BL/6J were pre-cultured with 10 μg/mL LGp40 or CDp40 for 6 h, and then re-stimulated with 100 μg/mL HDM for another 6 h. Thereafter, harvested cells were washed twice with sterile PBS, and were then directly prepared for cDNA amplification and RNA-Seq library construction (detailed information is provided in Additional file 2).

### Comparison of LGp40 and CDp40 structures

A structural model of CDp40 binding with NAD^+^ was generated with SWISS-MODEL [32, 33], a fully automated protein structure homology-modeling server. We used the PDB accession code 1GD1 (*Geobacillus stearothermophilus*’ GAPDH complexed with NAD^+^) as the template. Prior to generating the comparison and third dimension alignment structural figure, the crystal structure of LGp40 and the modeling structure of CDp40 were superimposed with the secondary structure matching algorithm of the PDBeFold server [34].

### Statistical analysis

All data are presented as mean ± SEM and analyzed using either two-way or one-way ANOVA, with Bonferroni’s multiple comparison, using GraphPad Prism (San Diego, CA, USA).

## Results

### Characterization of LGp40

**Fig. 1:**
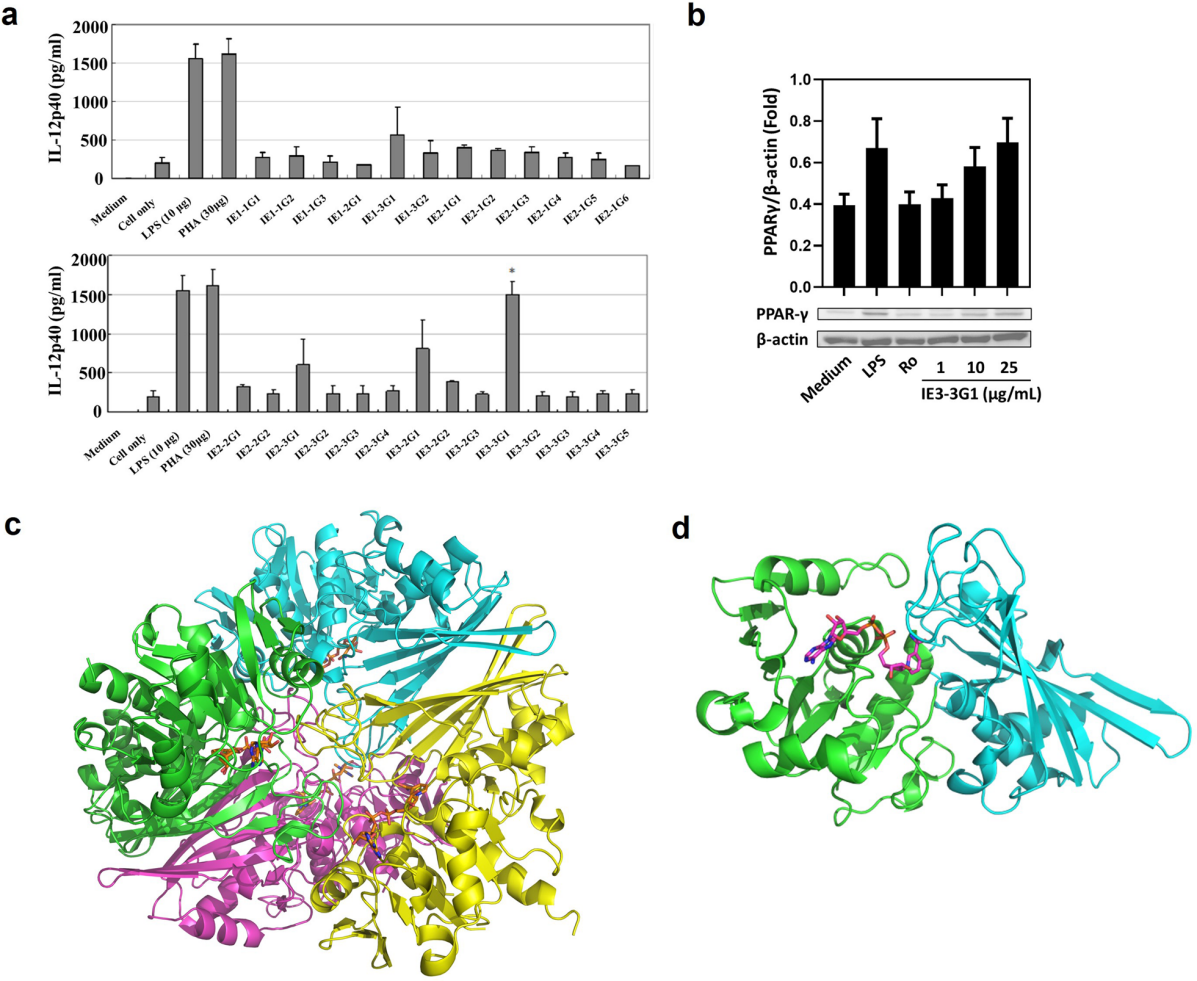
Identification of active ingredients, LGp40, from lysed *L. gasseri* components. **a** IL-12p40 levels of the sub-fraction IE1-1G1 to IE3-3G5 (sub-fractions identified on size-exclusion chromatography) stimulated mouse BMDC. **b** PPARγ expression of sub-fraction IE3-3G1 stimulated mouse BMDC. The production of IL-12p40 and PPARγ was detected with ELISA and Western blot assay, respectively (n = 3, *p < 0.05, one-way ANOVA with Bonferroni multiple comparison test). **c** The tetrameric assembly (biological assembly) of GAPDH of *L. gasseri* is shown (LGp40). The structure representing the tetrameric assembly of the LGp40 molecules through crystal symmetry is depicted. Individual protein subunits are represented as cartoon diagrams and painted in different colors. **d** The LGp40 molecule is represented as a cartoon diagram with green indicating the NAD(P)-binding domain (residues 3–156 and 321–338) and cyan indicating the C-terminal domain (residues 156–320). NAD^+^ molecules are shown as ball-and-stick models. The carbon, nitrogen, oxygen, and phosphate atoms of the NAD^+^ molecule are shown in magenta, blue, red, and orange, respectively. The covalent bonds between the atoms of the NAD^+^ molecule are shown in light gray

To identify the active component of *L. gasseri* that may have beneficial functions on allergic asthma, the bacterial cellular lysates were first precipitated by ammonium sulfate and then subjected to ion-exchange and size-exclusion chromatography for fractional analysis (detailed information is provided in Additional file 1 which includes the explanation of Additional file 3: Tables S1, S2, and Figs. S1–S6). The sub-fraction, named IE3-3G1, was selected based on its highest immunomodulatory ability among other eluent fractions as it upregulated the most amount of IL-12p40 in mouse BMDC (Fig. 1a). Furthermore, IE3-3G1 enhanced PPARγ expression in mouse BMDC in a dose-dependent manner (Fig. 1b). Next, 10% SDS-PAGE electrophoresis showed that IE3-3G1 comprised a major protein band above 36 kDa and a minor protein band below 36 kDa, whereas the native polyacrylamide gel electrophoresis demonstrated a single major band (Additional file 3: Fig. S7a). With 2DE analysis, we found six protein spots in total, including five protein spots in 37 kDa and one protein spot in 25 kDa (Additional file 3: Fig. S7b). Proteomics analysis using LC–MS/MS revealed that protein spots 1–5 were GAPDH, and protein spot 6 was fructose/bisphosphate aldolase (raw data are provided in Additional file 4). Therefore, we identified the sub-fraction of IE3-3G1 that contained proteins, which mostly belongs to GAPDH, an enzyme that catalyzes the classical glycolysis reaction. GAPDH from *L. gasseri* was further cloned into a pET-21b vector carrying a His-tag. The His6-GAPDH of *L. gasseri* eluted as a 37- kDa protein, which was named LGp40, and was detected by His-tag antibody (Additional file 3: Fig. S7c). LGp40 was further subjected to analyze X-ray crystallography. We determined the crystal structure of LGp40 to a resolution of 1.88 Ǻ. Data collection and refinement statistics are summarized in Additional file 3: Table S3. The crystal of LGp40 belongs to the tetragonal space group *I*4_1_22 (International Tables for Crystallography: https://it.iucr.org/). The crystallographic asymmetrical unit contains one copy of the LGp40 molecule that associates through symmetry to form a tetramer (Fig. 1c). The structure of LGp40 can be divided into the following two domains: an N-terminal NAD(P)-binding domain (residues 3–156) and a catalytic C-terminal domain (residues 161–318) (Fig. 1d).

### Administration of LGp40-overexpressing *Clear coli* attenuates airway inflammation in HDM-induced asthmatic mice

**Fig. 2:**
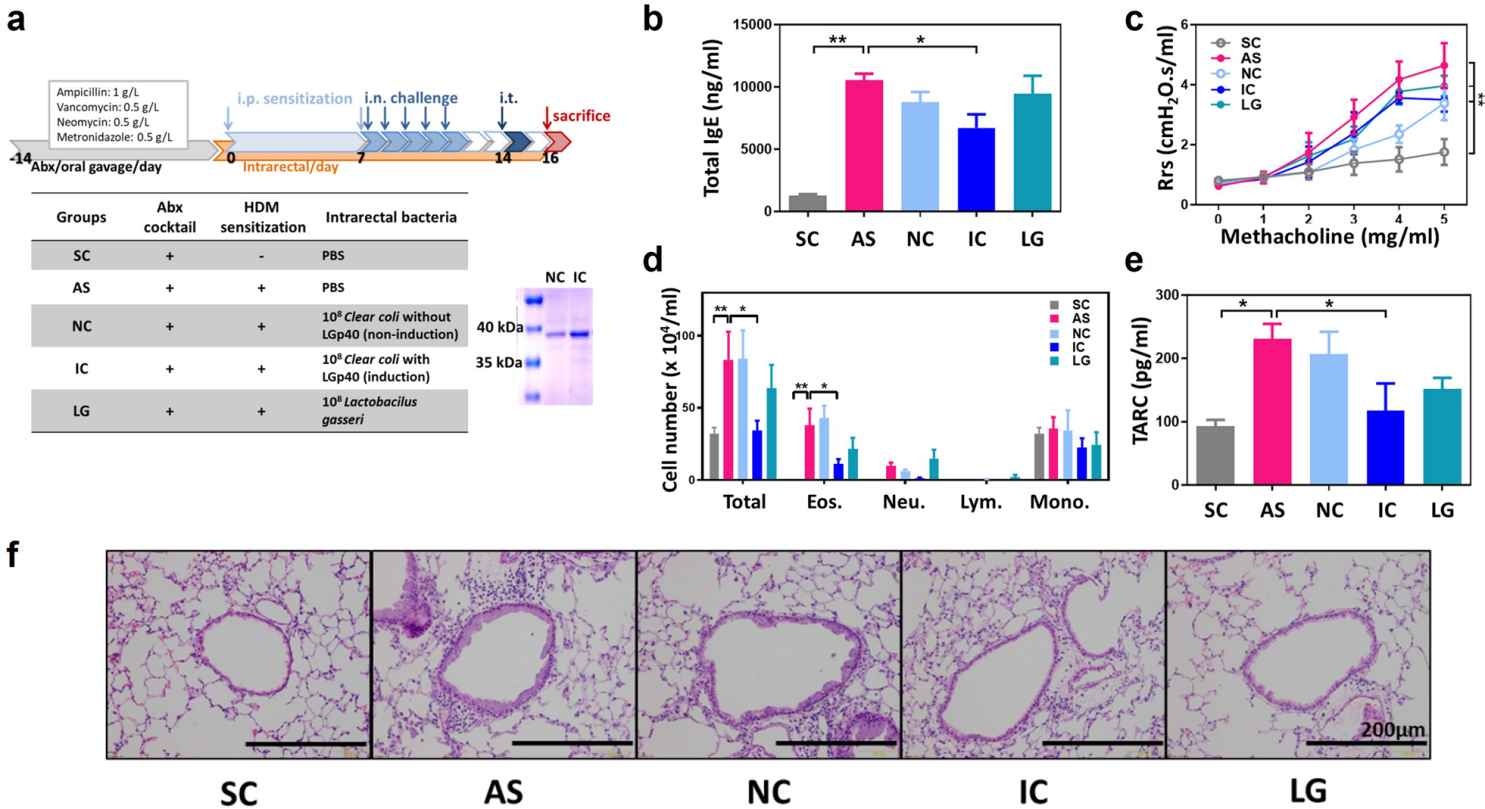
Intrarectal administration of LGp40-overexpressing *Clear coli* alleviated HDM-induced asthma in mice. **a** Experimental scheme of Protocol 1. SDS-PAGE showed the quantities of LGp40 derived from IPTG-non-induced and IPTG-induced *Clear coli*. **b** Total IgE serum concentrations were measured using ELISA (n = 10 mice, *p < 0.05 and **p < 0.01, one-way ANOVA with Bonferroni multiple comparison test). **c** Rrs for increasing dosages of aerosolized methacholine were measured using the flexiVent FX system (n = 10 mice, *p < 0.05 and **p < 0.01, two-way ANOVA with Bonferroni multiple comparison test). **d** Total cell, eosinophil, neutrophil, lymphocyte, and macrophage counts in the BALF were determined (n = 10 mice, *p < 0.05 and **p < 0.01, one-way ANOVA with Bonferroni multiple comparison test). **e** TARC production in BALF was determined using ELISA (n = 10 mice, *p < 0.05, one-way ANOVA with Bonferroni multiple comparison test). **f** Lung histology. Lung sections were stained with hematoxylin and eosin (H&E; scale bar = 200 μm). Results comprise pooled data from two independent experiments

To evaluate the functional role of LGp40 in HDM-induced allergic asthma, IPTG-induced LGp40-overexpressing *Clear coli*, IPTG non-induced *Clear coli*, and *L. gasseri* were intrarectally inoculated daily during HDM sensitization and challenge periods in mice with allergic asthma (Fig. 2a). According to the protein electrophoresis, mice in the IC group were inoculated with LGp40-overexpressing *Clear coli*, whereas mice in the NC group received LGp40 spontaneously expressing minor amount of *Clear coli* (Fig. 2a). AS mice developed the hallmarks of allergic asthma, with noticeably elevated serum total IgE concentrations (Fig. 2b) and Rrs (Fig. 2c). Among the three bacterial intrarectal groups, only the IC group had significantly decreased total IgE production compared with AS mice (Fig. 2b). However, there was no significant difference in Rrs compared with AS mice (Fig. 2c). In the BALFs collected 2 days after allergen challenge, decreased inflammatory cells and eosinophils infiltration (Fig. 2d) and TARC levels (Fig. 2e) were found in both IC and LG groups as compared with AS mice. The reduced mucus production in the bronchial epithelia and peri-bronchial areas were also found in both IC and LG groups (**Fig. ****2****f**). However, these parameters in BALF were significant only in the IC group.

### LGp40, but not CDp40, inhibited asthma in HDM-sensitized mice

**Fig. 3:**
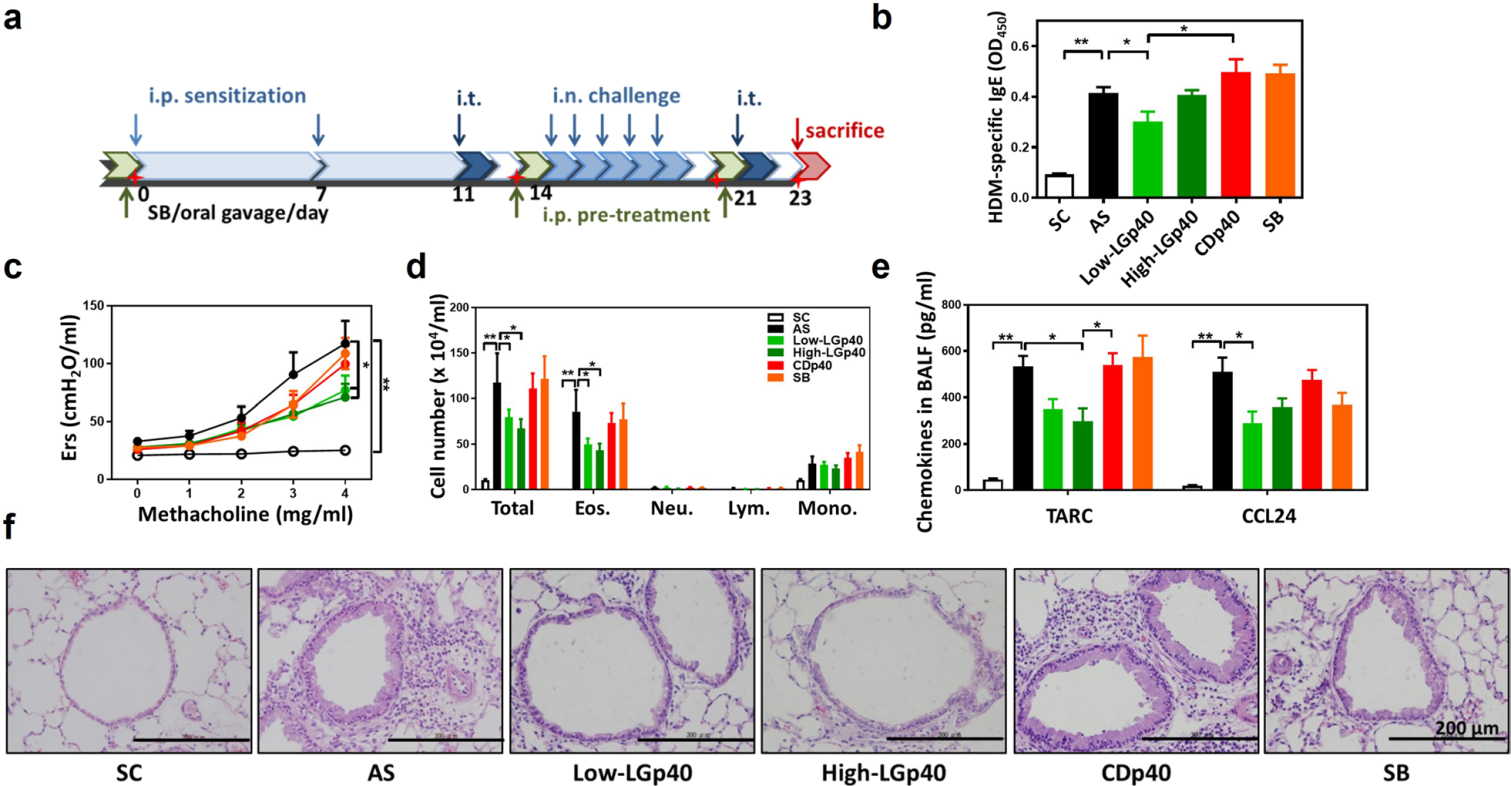
Intraperitoneal administration of LGp40, rather than CDp40, suppressed HDM-induced asthma. **a** Experimental scheme of Protocol 2. **b** HDM-specific IgE serum concentrations were measured using ELISA (n = 15 mice, *p < 0.05 and **p < 0.01, one-way ANOVA with Bonferroni multiple comparison test). **c** Ers for increasing dosages of aerosolized methacholine were measured using the flexiVent FX system (n = 15 mice, *p < 0.05 and **p < 0.01, two-way ANOVA with Bonferroni multiple comparison test). **d** Total cells, eosinophils, neutrophils, lymphocytes, and macrophages in the BALF were counted (n = 15 mice, *p < 0.05 and **p < 0.01, one-way ANOVA with Bonferroni multiple comparison test). **e** TARC and CCL24 production in BALF were determined using an ELISA assay kit (n = 15 mice, *p < 0.05, one-way ANOVA with Bonferroni multiple comparison test). **f** Lung histology. Lung sections were stained with H&E (scale bar = 200 μm). Results comprise pooled data from three independent experiments

To compare the function of GAPDH from different strains of probiotics and pathogenic bacteria, we aligned the GAPDH protein sequences from four probiotic species (*L. gasseri, L. reuteri, L. plantarum, and B. longum*) and four pathogenic species (*C. perfringens, C. difficile, S. enterica, and E. coli*). The phylogenetic study showed that GAPDH of *L. gasseri* shared more similarities with GAPDH from probiotics (66–84%) and less with GAPDH from pathogens (44–57%) (Additional file 3: Fig. S8a). In addition, His-tagged GAPDH from *L. gasseri* (LGp40) and *L. reuteri* (LRp40) have dehydrogenase activity, but not the His-tagged GAPDH from *C. difficile* (CDp40) with enzyme activity (Additional file 3: Fig. S8b). To further investigate whether the diverse characteristics of LGp40 and CDp40 may undertake different regulation in allergic inflammation, low and high doses of LGp40 and low dose of CDp40 were intraperitoneally administered to AS mice. Sodium butyrate was orally administered as a control (Fig. 3a). The Low-LGp40 group showed significantly lower HDM-specific IgE levels compared with untreated and CDp40-treated AS mice (Fig. 3b). Both LGp40-administered mice exhibited a notable reduction of Ers (Fig. 3c) and total inflammatory cells and eosinophils infiltration in the lung (Fig. 3d) compared with AS mice. Reductions in TARC and CCL24 levels were observed in the BALF obtained from the LGp40-treated mice (Fig. 3e). The High-LGp40 group also showed significantly decreased TARC levels compared with the CDp40 group (Fig. 3e). Histological analysis demonstrated decreased mucus production and reduced inflammatory cell infiltration in both LGp40-administered groups and the SB group. Conversely, the CDp40 group developed aggravated inflammation in lung pathology (Fig. 3f).

### LGp40 inhibited M2 and enhanced M1 phenotype of macrophages to improve HDM-induced allergic inflammation

**Fig. 4:**
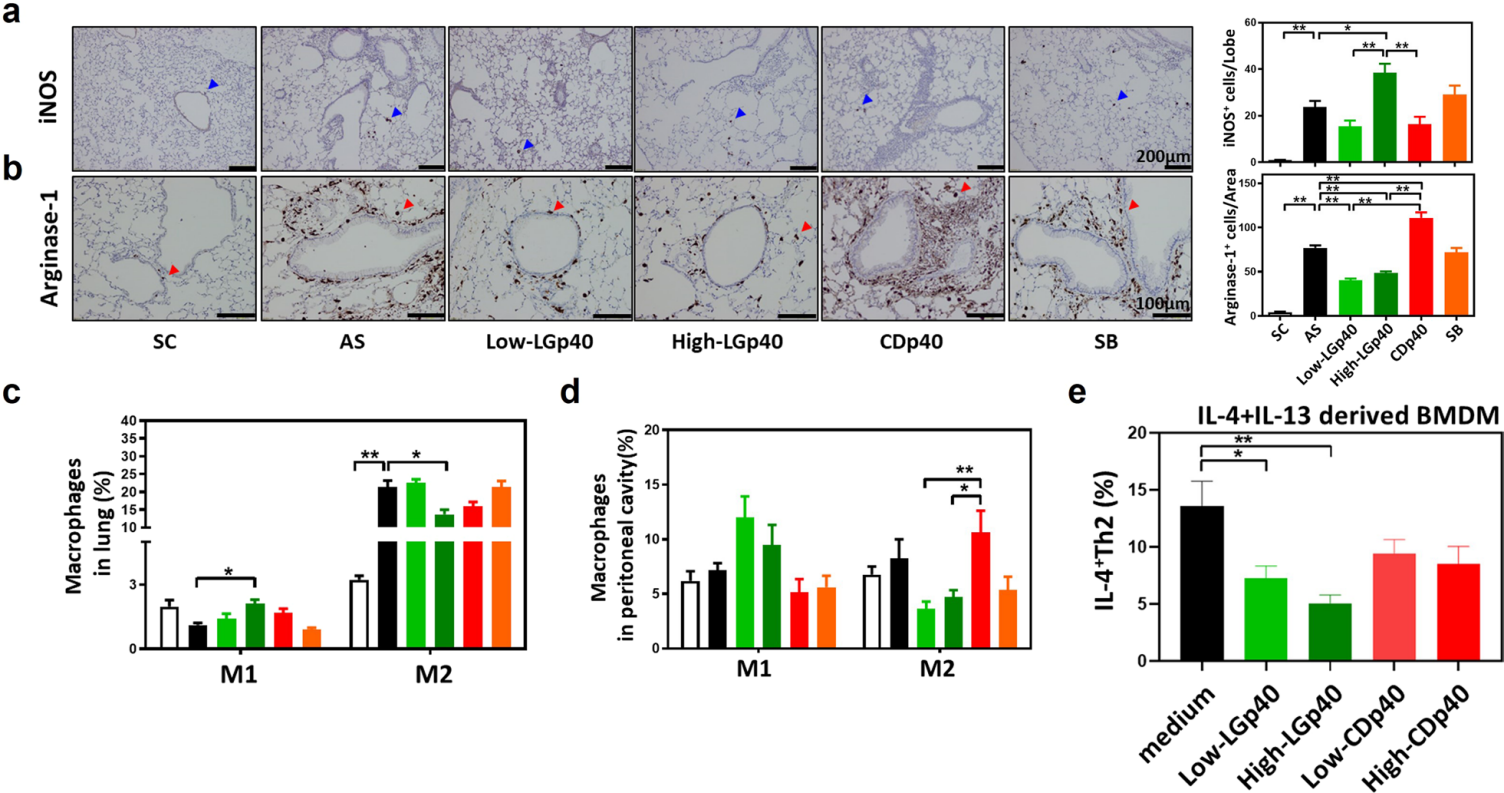
Intraperitoneal administration of LGp40 inhibited M2 macrophage accumulation and promoted accumulation of M1 macrophages in the lung of mice with HDM-induced asthma. Lung sections were immunostained with **a** iNOS and **b** arginase-1 antibodies and then reacted with diaminobenzidine (scale bars = 200 and 100 μm). The iNOS-positive cells (blue arrows) were counted in every lobe per group, and the average numbers were calculated. The arginase-1-positive cells (red arrows) were counted in ×200 microscopic fields per group, and the average numbers were calculated (n = 6 mice, **p < 0.01, one-way ANOVA with Bonferroni multiple comparison test). M1 and M2 cell percentages **c** in single lung suspensions and **d** in peritoneal cavity were determined using flow cytometry analysis (n = 15 mice, *p < 0.05 and **p < 0.01, two-way ANOVA with Bonferroni multiple comparison test). **e** IL-4- and IL-13-stimulated BMDM were incubated with naïve Th cells in the presence of LGp40 and CDp40. IL-4^+^ Th2 cell populations were determined using flow cytometry analysis (n = 6 mice, *p < 0.05 and **p < 0.01, one-way ANOVA with Bonferroni multiple comparison test). Results comprise pooled data from three independent experiments

We further analyzed macrophage subsets in these mice to examine the immunomodulatory difference between LGp40 and CDp40 in allergic inflammation. Compared with untreated and CDp40 treated AS mice, the LGp40 groups displayed markedly increased iNOS^+^ M1 (Fig. 4a) and decreased arginase-1^+^ M2 in the lung (Fig. 4b). In contrast, the CDp40 group exhibited significantly higher arginase-1^+^ M2 populations than the untreated and LGp40-treated AS mice (Fig. 4b). The same results were found in single-cell suspension analysis of the lung and peritoneal fluid. The High-LGp40 group showed significantly increased M1 and decreased M2 populations in the lungs compared with AS mice (Fig. 4c). Both LGp40-administered groups showed increased M1 and decreased M2 in the peritoneal cavity, but not to a significant level, as compared with AS mice (Fig. 4d). Notably, both LGp40 groups had significantly lower peritoneal M2 population than the CDp40 group (Fig. 4d). RNA-seq analysis of HDM stimulated BMDM also demonstrated that LGp40 pretreatment upregulated M1-related genes and downregulated M2-related genes. CDp40 pretreatment conversely presented little influence on these genes (Additional file 3: Fig. S9). We then investigated whether the M2 phenotypic polarization caused by bacterial GAPDH proteins would affect allergic Th2 activation. The results showed that LGp40, rather than CDp40, dramatically suppressed M2-induced Th2 activation in a dose-dependent manner (Fig. 4e). Furthermore, LGp40 stimulation groups showed less IL-4^+^Th2 population than CDp40 groups (Fig. 4e).

### LGp40 had better ability at plasminogen interaction and enhancement of plasmin activation than CDp40

**Fig. 5:**
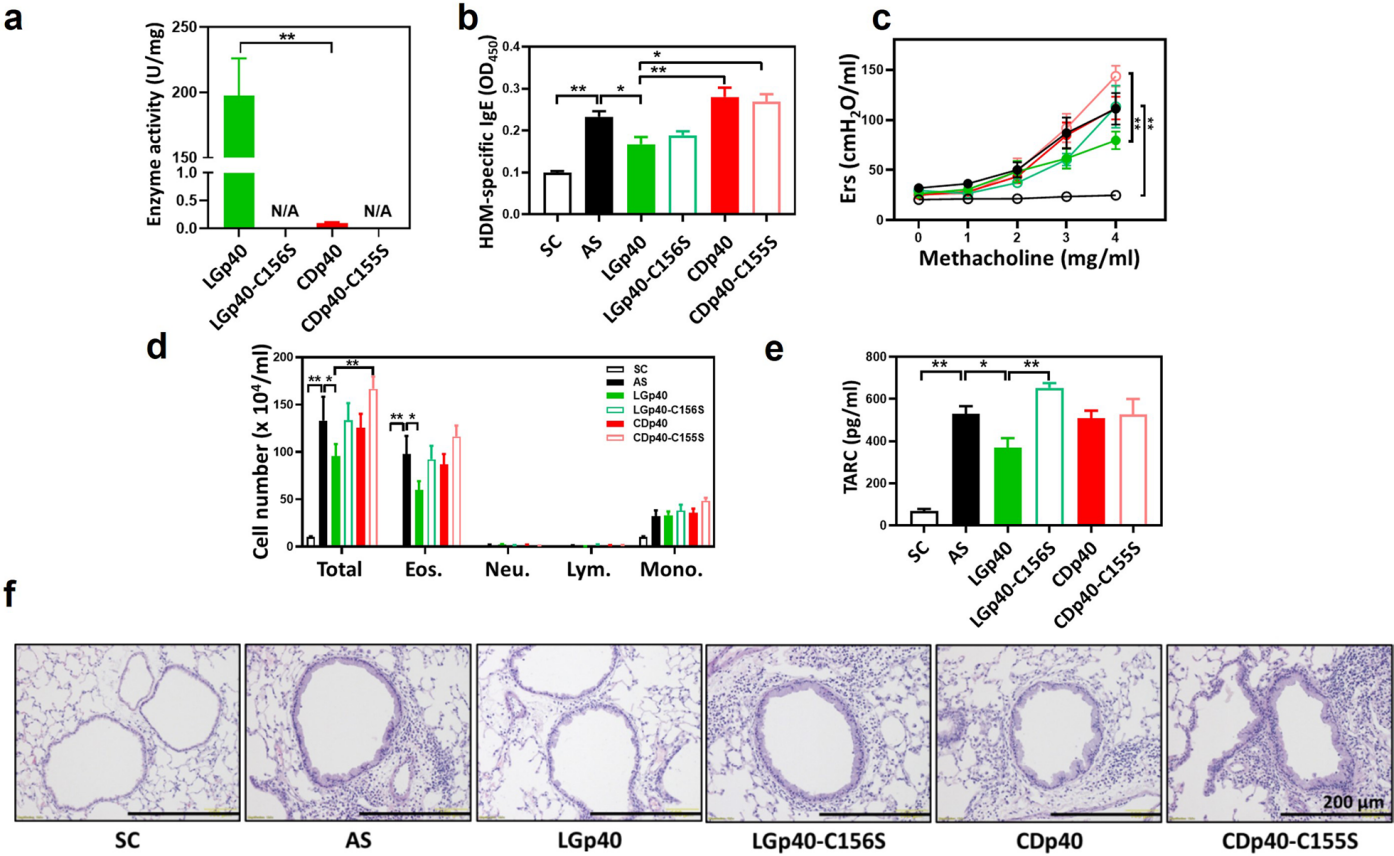
LGp40 failed to alleviate HDM-induced asthma when its enzymatic activity was lost. **a** Enzymatic activity of recombinant GAPDH proteins. **b** HDM-specific IgE serum concentrations were measured using ELISA (n = 5 mice, *p < 0.05 and **p < 0.01, one-way ANOVA with Bonferroni multiple comparison test). **c** Ers for increasing dosages of aerosolized methacholine were measured using the flexiVent FX system (n = 5 mice, *p < 0.05 and **p < 0.01, two-way ANOVA with Bonferroni multiple comparison test). **d** Total cells, eosinophils, neutrophils, lymphocytes, and macrophages in the BALF were counted (n = 5 mice, *p < 0.05 and **p < 0.01, two-way ANOVA with Bonferroni multiple comparison test). **e** TARC production in BALF was determined using an ELISA assay kit (n = 5 mice, *p < 0.05 and **p < 0.01, one-way ANOVA with Bonferroni multiple comparison test). **f** Lung histology. Lung sections were stained with H&E (scale bar = 200 μm)

To explore whether the diverse characteristics of LGp40 and CDp40 persisted in the regulation of host plasminogen/plasmin system and whether their different dehydrogenase activities were involved, we cloned a loss-of-function LGp40-C156S protein wherein the active cysteine site was mutated to serine. A loss-of-function CDp40-C155S protein was cloned as a control. LGp40 had markedly higher dehydrogenase activity than CDp40. Neither LGp40-C156S nor CDp40-C155S proteins had any enzymatic activity (Fig. 5a). Both LGp40 and CDp40 were plasminogen binders and could enhance uPA-mediated plasmin activation. Additionally, the plasminogen interaction and plasmin activation ability of LGp40 were more effective than those of CDp40. This action decreased when the enzyme activity of LGp40 was lost (Additional file 3: Fig. S10a, b). Furthermore, a competitive binding assay using EACA, a lysine analog, confirmed that lysine residues involved in the binding to host plasminogen and plasmin activation ability of bacterial GAPDH proteins (Additional file 3: Fig. S10c, d).

### Immunoregulatory function of LGp40 in AS mice was determined by its dehydrogenase activity

These four recombinant GAPDH proteins were further intraperitoneally injected to HDM-induced AS mice to examine whether the immune modulation effects between LGp40 and CDp40 were related to their different dehydrogenase activities. The results showed that LGp40 group exhibited significantly lower HDM-specific IgE concentrations than AS, CDp40, and CDp40-C155S groups (Fig. 5b). LGp40 group also had noticeably less Ers than CDp40-C155S group after methacholine stimulation (Fig. 5c). Of note, BALF analysis demonstrated that only LGp40-administered mice, and no other treatment groups, displayed a major reduction in total inflammatory cell, eosinophil (Fig. 5d), and TARC levels (Fig. 5e). Furthermore, LGp40 group exhibited significantly less total inflammatory cells than CDp40-C155S group (Fig. 5d), and significantly lower TARC levels than LGp40-C156S group (Fig. 5e). Histological analysis also showed reduced bronchial epithelial swelling and decreased inflammatory cells infiltration in LGp40-treated mice, but not in the other treated mice (Fig. 5f). Among them, LGp40-C156S, CDp40, and CDp40-C155S groups had similar worsen pathological lung features as those observed in AS group. CDp40-C155S group had the most increased inflammatory cell infiltration in the lung (Fig. 5f).

### LGp40 redirected macrophage polarization depend on its dehydrogenase activity

**Fig. 6:**
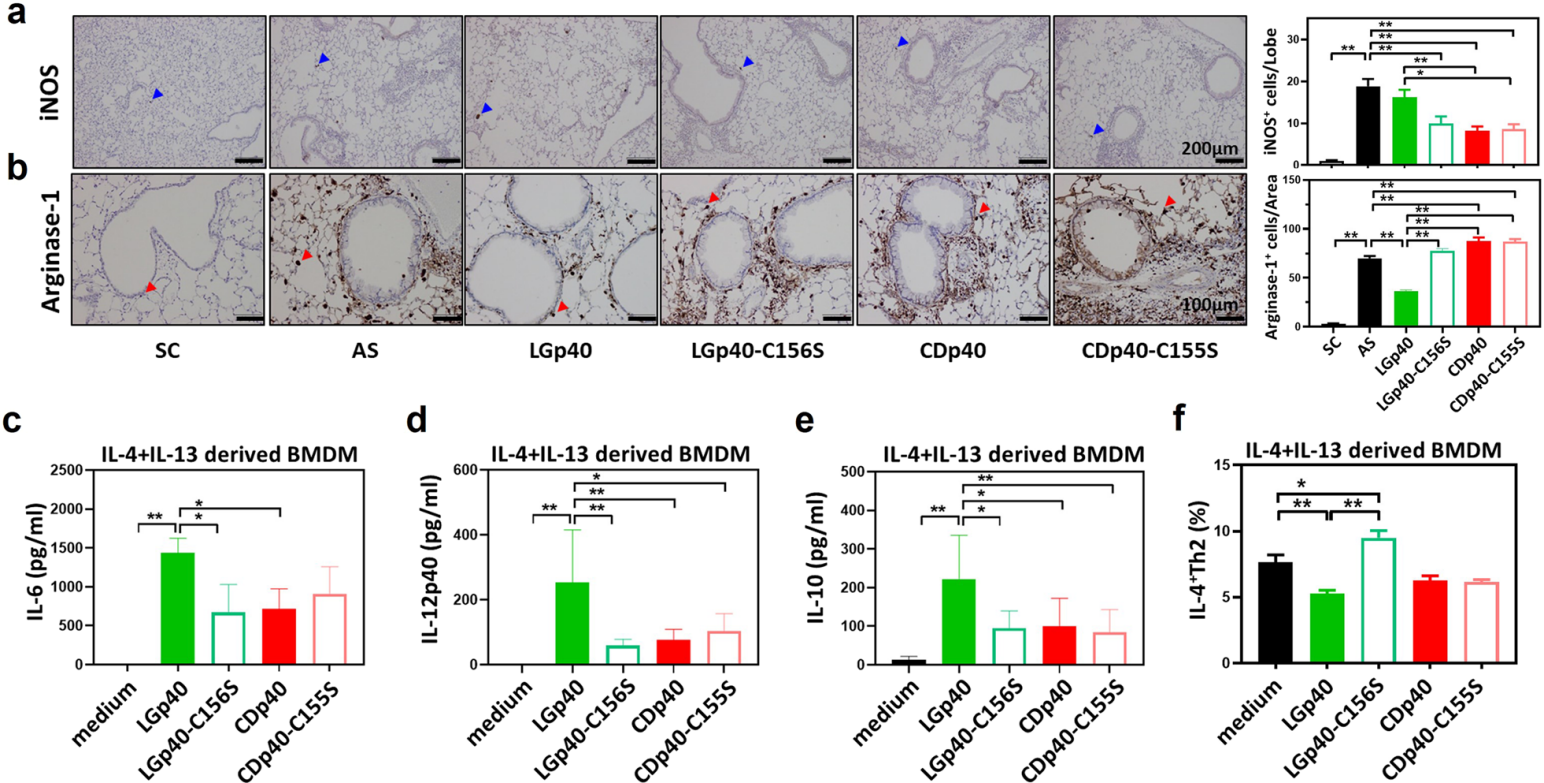
LGp40 failed to inhibit M2 macrophages in HDM-induced asthma when the enzymatic activity was lost. Lung sections were immunostained with **a** iNOS and **b** arginase-1 antibodies then reacted with diaminobenzidine (Scale bars = 200 and 100 μm). The iNOS-positive cells (blue arrows) were counted in every lobe per group, and the average numbers were calculated. The arginase-1-positive cells (red arrows) were counted in ×200 microscopic fields per group. The average numbers were calculated (n = 5 mice, **p < 0.01, one-way ANOVA with Bonferroni multiple comparison test). **c** IL-6, **d** IL-12p40, and **e** IL-10 production in cultured supernatants of IL-4- and IL-13-stimulated BMDM incubated with recombinant proteins were determined using an ELISA assay kit. **f** IL-4- and IL-13-stimulated BMDM were incubated with naïve Th cells in the presence of recombinant proteins. IL-4^+^ Th2 cell populations were determined using flow cytometry analysis (n = 6 mice, *p < 0.05 and **p < 0.01, one-way ANOVA with Bonferroni multiple comparison test). Results comprise pooled data from three independent experiments

From the IHC analysis, we observed that the low enzyme active LGp40-C156S-, CDp40-, and CDp40-C155S-treated mice showed significantly lower iNOS^+^ M1 than the untreated AS mice. In contrast, LGp40-treated mice, with high enzyme activity, displayed more iNOS^+^ M1 expression than the CDp40 and the CDp40-C155S groups (Fig. 6a). Moreover, the LGp40 group also exhibited a remarkable reduction in arginase-1^+^ M2 among treated groups. The other treated groups showed significantly higher arginase-1^+^ M2 than untreated and LGp40-treated AS mice (Fig. 6b). After stimulation with high enzyme active LGp40, IL-4- and IL-13-derived M2 phenotypic BMDM had significantly elevated levels of M1 cytokines, IL-6 and IL-12p40, in the cultured supernatants (Fig. 6c, d). IL-10, an anti-inflammatory cytokine, was markedly elevated (Fig. 6e). In contrast, the low enzyme active CDp40-treated and loss-of-function protein-treated BMDM showed lower levels of IL-6, IL-12p40, and IL-10 compared with LGp40-treated BMDM (Fig. 6c–e). When these pretreated M2 phenotypic BMDM were co-cultured with naïve Th cells, only high enzyme active LGp40 group inhibited M2-induced Th2 activation. The loss-of-function LGp40-C156S group, on the other hand, promoted M2-derived Th2 activation (Fig. 6f).

### NAD^+^ binding activity of LGp40 enhances glycolytic metabolism in M2 macrophages

**Fig. 7:**
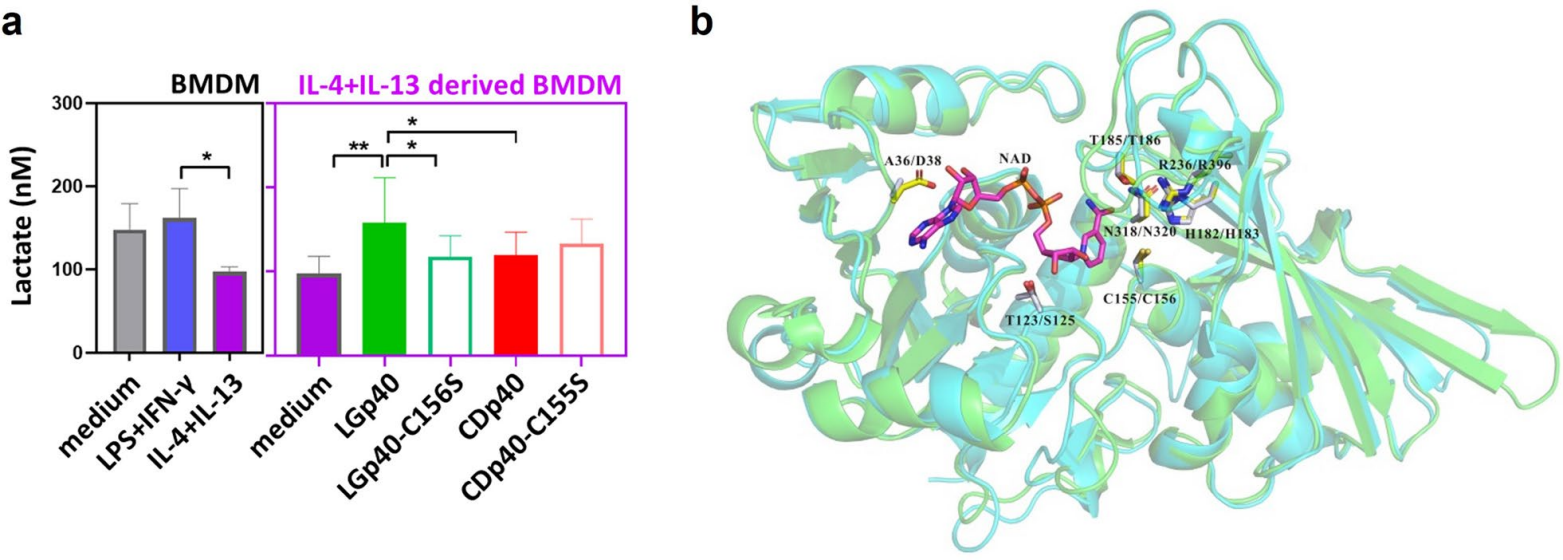
LGp40, rather than CDp40, triggered glycolysis in allergic M2 macrophages. **a** Lactate production in cultured supernatants of IL-4- and IL-13-stimulated BMDM that were incubated with recombinant proteins were determined using an ELISA assay kit (n = 6 mice, *p < 0.05 and **p < 0.01, one-way ANOVA with Bonferroni multiple comparison test). Results are pooled data from three independent experiments. **b** The superimposed structures of LGp40 (green) and CDp40 (cyan) in a schematic representation. The key residues surrounding the NAD-binding sites of LGp40 and CDp40 are indicated. The side chains of the residues around the NAD(P)-binding sites and NAD^+^ molecule are shown as ball-and-stick models. The carbon atoms of the LGp40, the CDp40, and NAD^+^ molecules are shown in yellow, light gray, and magenta, respectively. Oxygen atoms are shown in red, nitrogen in blue, and phosphate in orange. The residues marked at the front (A36, T123, C155, H182, T185, R236, and N318) are key residues that surround the NAD(P)-binding site of CDp40. The corresponding residues (D38, S125, C156, H183, T186, R239 and N320) in LGp40 are marked in back

LPS- and IFN-γ-stimulated M1 phenotypic BMDM had higher lactate levels than IL-4 and IL-13- exposed M2 phenotypes (Fig. 7a), suggesting that anaerobic M1 macrophages rely on glycolysis. Instead, the aerobic M2 phenotype is based on oxidative phosphorylation [35]. After exposure to high enzyme active LGp40, M2 phenotypic BMDM showed higher lactate production, indicating that these cells shift to glycolytic metabolism (Fig. 7a). Conversely, neither the stimulation of low enzyme active CDp40, nor loss-of-function proteins changed the lactate concentration in M2 phenotypic BMDM (Fig. 7a). To interpret the significant difference of LGp40 and CDp40 from a structural perspective, the crystal structure of LGp40 and the modeling structure of CDp40 were superimposed. A significant difference in the NAD-binding site between the structures of LGp40 and CDp40 was revealed. The NAD-binding site of LGp40 is an Asp38 residue, which contributes two hydrogen-bond interactions with NAD^+^ to provide stronger binding forces (Fig. 7b). In contrast, the corresponding residue in CDp40 is Ala36. The side chain of Ala36 could not provide any stable interaction with NAD^+^ (Fig. 7b).

## Discussion

**Fig. 8:**
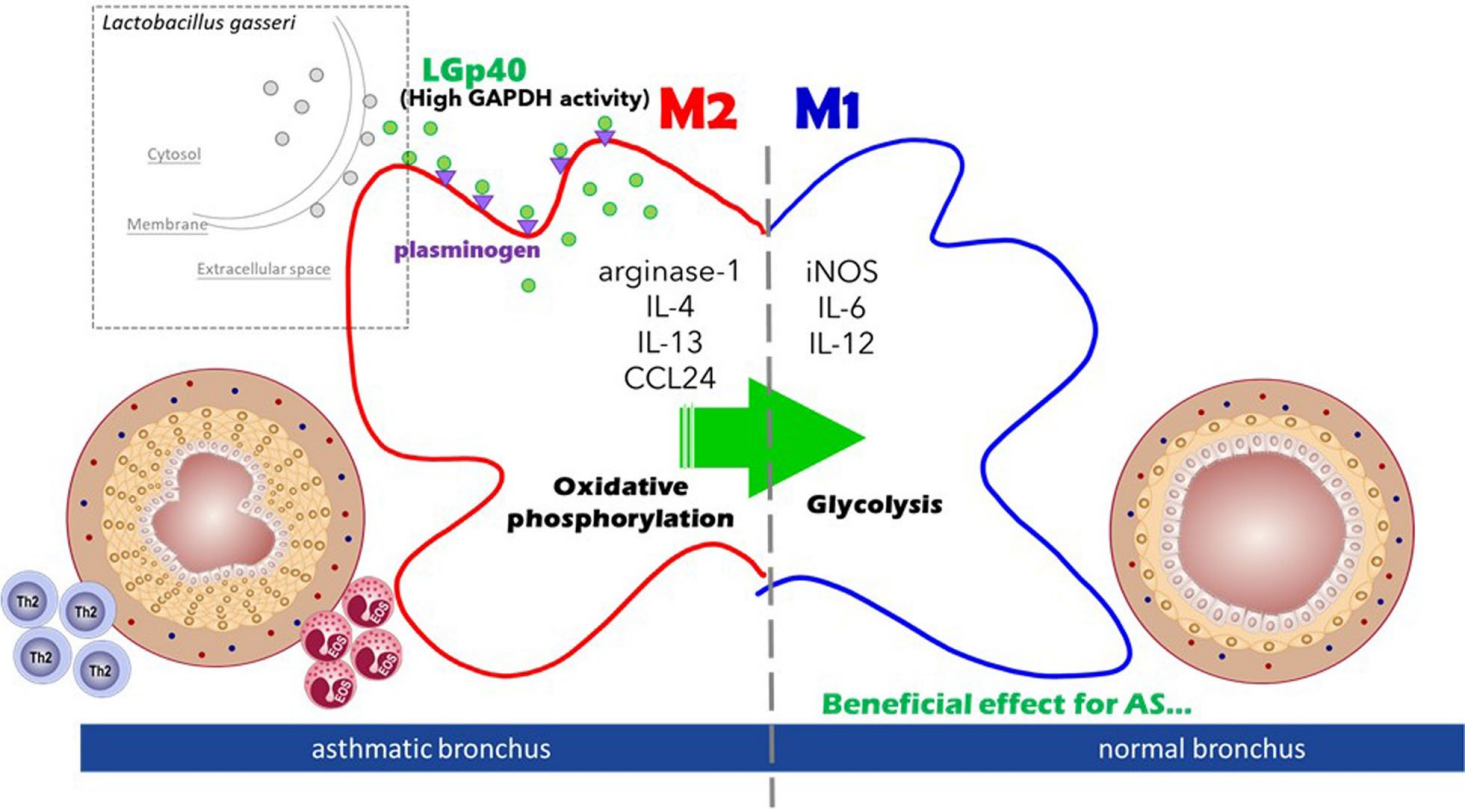
The diverse localization of LGp40 alters macrophage phenotypes with its high glycolytic activity. LGp40 adheres to plasminogen on macrophages and changes the metabolism of allergic M2 macrophage from oxidative phosphorylation to glycolysis, including macrophage polarization toward M1. The moonlighting LGp40 modulates macrophage reprogramming with dehydrogenase activities to alleviate the progression of allergic inflammation of airways. (The graph was generated using BioRender software.)

In this study, we identified and purified the GAPDH protein from *L. gasseri* (LGp40)*,* which performs moonlighting activities including glycolysis and immunomodulation. Intrarectal inoculation of LGp40-overexpressing *Clear coli* and intraperitoneal administration of recombinant LGp40 proteins in HDM-induced AS mice induced attenuation of allergic inflammation. This confirmed the protective effect of LGp40 in AS mice. In contrast, CDp40-treated mice displayed aggravated airway inflammation in experimental asthma, revealing the different immunomodulatory abilities between probiotic and pathogenic GAPDH proteins. In fact, the glycolytic enzyme GAPDH has been shown to be a multi-tasking protein with virulence impact in a number of pathogenic bacteria [11]. This protein can be detected on the bacterial surface or secreted outside the bacterial cells to interact with host proteins [12]. To our knowledge, we are the first to confirm that probiotic and pathogenic GAPDH may share similar functions in adhering to host plasminogen, but they have distinct immunomodulatory activities in allergic asthma depending on their respective dehydrogenase activities (Fig. 8).

It is controversial whether the plasminogen/plasmin system plays pro-inflammatory or anti-inflammatory roles in allergic asthma development [36]. Both plasminogen/plasmin-deficient mice and administration of plasminogen activator inhibitor 1 that promotes plasmin activation successfully reduced allergic inflammation [37, 38]. In this study, our results show that only the stronger plasmin activator-LGp40 alleviates allergic inflammation. Plasminogen and plasmin have also been shown to induce macrophage reprogramming to anti-inflammatory phenotypes that prevent the progression of chronic inflammation [39]. Plasmin-stimulated monocyte-derived dendritic cells induce T cell differentiation into Th1, but not Th2 [40]. Here, we found that both LGp40 and CDp40 are plasminogen binders (Additional file 3: Fig. S10). As plasminogen is the target receptor for bacterial GAPDH on host macrophages [41], we speculated that LGp40 could target to host macrophages and then increased the M1 population and decreased M2 phenotypes in asthmatic mice. Meanwhile, CDp40 maintained allergic M2 subsets according to IHC, flow cytometry, and RNA-seq analyses (Fig. 4 and Additional file 3: Fig. S9). As we did not detect changes in the population of Th and ILC subsets in the lungs (data not shown) after LGp40 and CDp40 administration, we concluded that LGp40 may redirect macrophages from M2 toward M1 and thereby improve airway allergic inflammation. CDp40, on the other hand, triggered allergic asthma progression through maintaining the M2 population. As LGp40 had better enhancement of plasmin activation (Additional file 3: Fig. S10b), further investigation of whether this difference in plasmin activation between LGp40 and CDp40 is involved in their different immunomodulatory effects in macrophages is required.

The metabolic changes in immune cells have crucial roles in their inflammatory or regulatory responses [42]. Macrophage polarization implicates metabolic reprogramming in their functional change in immune response. The function of non-allergic M1 macrophage mainly relies on glycolysis, while the property of allergic M2 macrophage depend on oxidative phosphorylation. Their polarization can be redirected by metabolism modulation [35]. Since GAPDH is an essential glycolytic enzyme, it proposed that whether the LGp40-induced M1 population in AS mice is dependent on its high dehydrogenase activity. Our results showed that the high enzyme active LGp40 induced iNOS^+^ cells (the metabolic enzymes of M1) and reduced arginase-1^+^ cells (the metabolic enzymes of M2), and the low enzyme active CDp40 and loss of dehydrogenase activity proteins generated different phenomena in AS mice (Fig. 6). M2 phenotypic BMDMs shifted to glycolysis metabolism after LGp40 exposure. Conversely, CDp40 and the loss-of-function proteins failed to change allergic M2 macrophage metabolic reprogramming, which augmented allergic inflammation (Fig. 6). These findings are similar to those of previous studies on glycolysis inhibitors [43, 44]. Therefore, it is possible that LGp40, which has high GAPDH activity, may change M2 macrophage metabolism and cause phenotype polarization toward M1.

Macrophages are a heterogeneous population that display a combination of inflammatory and anti-inflammatory functions. At present, there is consensus that M2a cell is elicited by IL-4- and IL-13 and secret high levels of IL-13 and chemokines, including TARC and CCL24, that activate Th2 immunity and promotes eosinophil trafficking, which induces allergic asthma. M2b, which is induced by IL-1 receptor ligands, immune complexes, and LPS, activates Treg cells, which leads to the development of allergic tolerance and deceased inflammation. M2c, which is stimulated by IL-10 and transforming growth factor (TGF)-β, have greater expression of anti-inflammatory IL-10, which is involved in the modulation of wound healing and anti-inflammatory responses. In contrast, LPS- and IFN-γ-stimulated M1 macrophages caused Th1 activation via IL-6 and IL-12 production, and suppressed tumor necrosis factor (TNF)-α, thereby inducing non-allergic inflammation [2]. In our study, we have shown that LGp40-treated AS mice had decreased levels of the chemokines TARC and CCL24 in the BALF as compared to AS mice (Fig. 3e**)**. Furthermore, in vitro BMDM experiments showed that LGp40 could elevate IL-6 levels and enhance IL-12p40 production in IL-4- and IL-13-derived M2a phenotypic BMDMs (Fig. 6c, d). Therefore, we suggested that, in this study, LGp40 may induce the transition of allergic M2a macrophages into non-allergic M1 phenotypes. Furthermore, LGp40 stimulation elevated IL-10 production in these IL-4- and IL-13-derived BMDMs (Fig. 6e), suggesting that LGp40 may redirect M2a into an M2c phenotype. The impact of GAPDH on macrophage polarization has been reported previously, but with the opposite effect. Nakano et al. reported that exogenous GAPDH has an anti-inflammatory function against LPS-stimulated M1 macrophages by suppressing TNF-α production and elevating IL-10 levels, which similarly depends on dehydrogenase activity [45]. This suggested that GAPDH may transform inflammatory M1 macrophages into M2 phenotypes, but specifically into the anti-inflammatory M2c macrophages under a Th1-dominant status. Depending on the circumstance, extracellular GAPDH may modulate macrophage polarization. Alternatively, as in our study, the results showed that under allergic Th2-dominant condition, LGp40 may induce the transformation of allergic M2a macrophages into non-allergic M1 phenotypes, and induces inflammatory M2a macrophages to transition into anti-inflammatory M2c macrophages. Therefore, regardless of Th1 or Th2-dominant conditions, GAPDH could regulate inflammatory macrophages into resting states.

The administration of LGp40-overexpressing *Clear coli* induced similar effects as those of *L. gasseri* in the attenuated allergen-induced airway inflammation in experimental AS mice (Fig. 2). These effects suggested that GAPDH may be one of the active anti-asthma components of *L. gasseri*. Previous studies of the mechanism of how probiotics alleviate allergic inflammation are mostly focused on their metabolites, which act as communicating signals for immune cell development and provide anti-inflammatory effects [46–49]. Trompette et al. reported that short- chain fatty acids (SCFAs), generated by intestinal microbiota, modulate the severity of allergic inflammation in the lung by skewing systemic innate immune cells toward less inflammatory phenotypes with impaired Th2-stimulatory abilities [46]. Other studies have demonstrated that butyrate not only limits ILC2 activation and eosinophil trafficking but also promotes immune tolerance through the induction of colonic Tregs to suppress allergic responses in the airway [47–49]. These soluble metabolites are considered a source for the gut microbiota and lung interaction via the circulation [50]. Similarly, probiotic GAPDH proteins have been reported that can rapidly modify its location in the bacterial cells whenever environmental pH is altered [51]. We believe that GAPDH constitutes a novel strategy by probiotics to alleviate allergic inflammation. Unlike SCFAs that target both innate and adaptive immunity to control allergic inflammation, bacterial GAPDH proteins have minimal effect in the regulation of host adaptive immunity (data not shown). These moonlighting dehydrogenases modulate host innate immunity, particularly through macrophage reprogramming and repolarization, to alter the progress of airway inflammation in asthma.

Probiotic supplementation is emerging as a safe and natural strategy for inhibiting the onset of allergic diseases. However, clinical probiotic intervention studies have so far yielded contradictory outcomes [52]. Increasing issues of host-associated factors such as sex, age, genetic makeup, and resident microbiological composition might determine an individual’s response to a specific probiotic treatment. These factors are thought to influence the marked difference in probiotic strains colonization between individuals [53]. Taken together, outcomes of probiotic prevention in the clinical setting are greatly influenced by host factors. It is crucial to investigate the active anti-allergy components of probiotics to overcome the uncertainty of whether microbes will survive and function in recipients. In this study, we identified a novel mechanism of moonlighting undertaken by LGp40 from *L. gasseri* for reversing M2-prompted Th2 cell activation through its glycolytic activity, which has an important immunoregulatory role in preventing allergic asthma. Therefore, our study’s results provide a new strategy in the application of probiotics to prevent or treat allergic disorders, such as asthma.

## Conclusions

LGp40 protein administration redirected macrophages to transition from the M2 toward M1 phenotype and suppressed Th2 activation. In vivo LGp40 administration attenuated airway inflammation in a mouse model of HDM-induced allergic asthma. In contrast, CDp40, the GAPDH from pathogenic *C. difficile*, promoted asthma exacerbation. Mutant LGp40 that lost its enzymatic effect failed to alleviate allergen-induced airway inflammation in experimental asthma, indicating that the LGp40 dehydrogenase activity is necessary for the anti-allergic effect of probiotics. The investigation of the active anti-allergy components of probiotics to dispel uncertainty on whether microbes will survive and function in recipients is a crucial topic.

## Supplementary Information

**Additional file 1. **Details of the identification of the anti-allergy fraction, IE3-3G1, from *L. gasseri.*

**Additional file 2. **Details of RNA-seq analysis.

**Additional file 3: Table S1.** Experimental protocol of ion-exchange chromatography. **Table S2.** Experimental protocol of size-exclusion chromatography. **Table S3.** Data collection and refinement statistics of the LGp40 crystal. **Figure S1.** IL-12p40 levels of crude extracts used to stimulate mouse BMDC. **Figure S2.** Ion-exchange chromatography of crude extracts on a DEAE-Sepharose Fast Flow column. **Figure S3.** IL-12p40 levels of IE1-1 to IE4-2 fractions (fractions from ion-exchange chromatography) used to stimulate mouse BMDC. **Figure S4.** Size-exclusion chromatography of fractions from crude extract IE1 on a Sephacryl S-300 HR column. **Figure S5.** Size-exclusion chromatography of fractions from crude extract IE2 on a Sephacryl S-300 HR column. **Figure S6.** Size-exclusion chromatography of fractions from crude extract IE3 on a Sephacryl S-300 HR column. **Figure S7.** Identification and purification of sub-fraction IE3-3G1. **Figure S8.** GAPDH derived from probiotics and pathogens are dissimilar. **Figure S9.** RNA-seq analysis showed differentially regulated gene expression between LGp40 and CDp40-stimulated BMDM. **Figure S10.** The plasminogen interaction and plasmin activation ability of LGp40 decreased when the GAPDH activity was lost.

**Additional file 4.** Raw data of sub-fraction IE3-3G1 proteomics analysis using LC–MS/MS.

## Data Availability

The raw sequence reads generated in this study have been deposited in the Sequence Read Archive (SRA) of the NCBI under accession number PRJNA816057. Crystallographic data of LGp40 have been deposited in the PDB under accession code 7WWW.

## Abbreviations

2DE: Two-dimensional gel electrophoresis
2D nano-HPLC–ESI–MS/MS: Two-dimensional nano-high performance liquid chromatography and electrospray ionization tandem mass spectrometry
BALF: Bronchoalveolar lavage fluid
BMDC: Bone marrow-derived dendritic cells
BMDM: Bone marrow-derived macrophages
CCL24: C–C motif chemokine ligand 24
EACA: ε-Aminocaproic acid
ELISA: Enzyme-linked immunosorbent assay
Ers: Airway elastane
FBS: Fetal bovine serum
GAPDH: Glyceraldehyde-3-phosphate dehydrogenase
GM-CSF: Granulocyte–macrophage colony-stimulating factor
HDM: House dust mite
HRP: Horseradish peroxidase
i.n.: Intranasal
i.p.: Intraperitoneal
i.r.: Intrarectal
IEF: Isoelectric focusing
IFN-γ: Interferon-gamma
IHC: Immunohistochemistry
IL: Interleukin
ILC2: Group 2 innate lymphoid cell
iNOS: Inducible nitric oxide synthase
IPTG: Isopropyl-β-d-thiogalactopyranoside
KEGG: Kyoto Encyclopedia of Genes and Genomes
LC–MS/MS: Liquid chromatography-tandem mass spectrometry
LPS: Lipopolysaccharides
PBS: Phosphate buffered saline
PDB: Protein Data Bank
PHA: Phytohemagglutinin
PVDF: Polyvinylidene difluoride
PPARγ: Peroxisome proliferator-activated receptor γ
Rrs: Airway resistance
Ro: Rosiglitazone
RIPA: Radioimmunoprecipitation assay
SCFA: Short-chain fatty acids
SDS-PAGE: Sodium dodecyl sulfate-polyacrylamide gel electrophoresis
TARC: Thymus- and activation-regulated chemokine
Th2: Type 2T helper cell
Treg: Regulator T helper cell
uPA: Urokinase-type plasminogen activator
