# Supplementary material for: Moonlighting glyceraldehyde-3-phosphate dehydrogenase (GAPDH) protein of Lactobacillus gasseri attenuates allergic asthma via immunometabolic change in macrophages
Source: J Biomed Sci. 2022 Sep 29;29:75. doi: 10.1186/s12929-022-00861-8 (PMC9520948; doi:10.1186/s12929-022-00861-8)
Supplement: Supplementary file 1 — Additional file 1. Details of the identification of the anti-allergy fraction, IE3-3G1, from L. gasseri. [file 12929_2022_861_MOESM1_ESM.docx]

**Additional file 1: Details of the identification of the anti-allergy fraction, IE3-3G1, from *L. gasseri.***

*Lactobacillus gasseri* was cultured from a single colony in Microprocessor Control Fermenter (model: Major Science, MS-F1) with MRS medium at 37°C for 5.5 hours. The 10^9^ of bacterial pellet was harvested through centrifuging at a high speed. After washed three times with phosphate buffered saline (PBS), the pelleted were homogeneously suspended in 50 mM Tris-HCl (pH 8.0) with 1% lysozyme on ice for 2 hours to release its cytoplasmic contents. The cytosolic fraction was than harvested through centrifugation at 22,500 × *g* for 30 minutes at 4°C. Afterward, ammonium sulfate (AS) was added incrementally at 0-25%, 25-50%, 50-75%, and 75-100% saturation to precipitate protein at 4°C. Following centrifugation at 22,500 × *g* for 30 minutes at 4°C, the fractionated proteins were dialyzed against PBS buffer in MW6000~8000 dialysis bag at 4°C for 24 hours. The crude extracts in 4 saturations were obtained as IE1 (AS 0-25%), IE2 (AS 25-50%), IE3 (AS 50-75%), and IE4 (AS 75-100%) at concentration 2.5 mg, 8 mg, 160 mg, and 18 mg, respectively. The 4 crude extracts (10 μg/mL) induced significantly higher IL-12p40 levels in mouse bone marrow derived dendritic cells (BMDC) than that of cell only group (**Additional file 3: Fig. S1**), suggesting that the proteins among them have immunomodulatory ability.

The crude extracts were further separated on ion-exchange chromatography system (BioLogic Duo Flow Chromatography System, Bio-Rad, Hercules, CA, USA) with DEAE-Sepharose Fast Flow column. Fraction proteins were eluted using 50 mM Tris-HCl (pH 8.5), containing sequential concentrations of NaCl (0 to 1 M). The detail of experimental protocol is shown in **Additional file 3: Table S1**. Three fraction, IE1-1 (19.58 μg/mL), IE1-2 (1.02 mg/mL), and IE1-3 (1.46 mg/mL), were isolated from crude extract IE1 (**Additional file 3: Fig. S2a)**; three fraction, IE2-1 (91.85 μg/mL), IE2-2 (118.06 μg/mL), and IE2-3 (9.12 mg/mL), were isolated from crude extract IE2 (**Additional file 3: Fig. S2b)**; three fraction, IE3-1 (23.159 mg/mL), IE3-2 (10.467 mg/mL), and IE3-3 (46.6 mg/mL), were isolated from crude extract IE3 (**Additional file 3: Fig. S2c)**; and two fraction, IE4-1 (3.72 mg/mL) and IE4-2 (35.24 mg/mL), were isolated from crude extract IE4 (**Additional file 3: Fig. S2d)**. These 11 fractions (10 μg/mL) were then co-cultured with mouse BMDC to evaluate its immune modulation ability. IL-12p40 production showed significantly higher when BMDC stimulated with IE1-1, IE1-2, IE1-3, IE2-1, IE2-2, IE2-3, IE3-2, and IE3-3 than that of cell only group (**Additional file 3: Fig. S3**).

Thereafter, these 8 fractions were additional divided on size-exclusion chromatography with a Sephacryl S-300 HR column. Sub-fraction proteins were eluted using 50 mM NaCl in 50 mM Tris-HCl (pH 9.5). The detail of experimental protocol is shown in **Additional file 3: Table S2.** Among fraction IE1-1 to IE1-3, three sub-fractions, IE1-1G1 (0.13 mg/mL), IE1-1G2 (0.21 mg/mL), and IE1-1G3 (2.32 mg/mL) were isolated from fraction IE1-1 (**Additional file 3: Fig. S4a)**; one sub-fraction, IE1-2G1 (0.14 mg/mL) was isolated from fraction IE1-2 (**Additional file 3: Fig. S4b)**; two sub-fractions, IE1-3G1 (0.25 mg/mL) and IE1-3G2 (0.15 mg/mL) were isolated from fraction IE1-3 (**Additional file 3: Fig. S4c).** Among fraction IE2-1 to IE2-3, six sub-fractions, IE2-1G1 (0.2 mg/mL), IE2-1G2 (0.21 mg/mL), IE2-1G3 (0.13 mg/mL), IE2-1G4 (0.16 mg/mL), IE2-1G5 (0.25 mg/mL), and IE2-1G6 (0.31 mg/mL) were isolated from fraction IE2-1 (**Additional file 3: Fig. S5a)**; two sub-fractions, IE2-2G1 (0.13 mg/mL) and IE2-2G2 (0.16 mg/mL) were isolated from fraction IE2-2 (**Additional file 3: Fig. S5b)**; four sub-fractions, IE2-3G1 (0.09 mg/mL), IE2-3G2 (0.19 mg/mL), IE2-3G3 (0.16 mg/mL),and IE2-3G4 (0.06 mg/mL) were isolated from fraction IE2-3 (**Additional file 3: Fig. S5c).** Among fraction IE3-2 to IE3-3, three sub-fractions, IE3-2G1 (0.24 mg/mL), IE3-2G2 (1.32 mg/mL), and IE3-2G3 (0.21 mg/mL) were isolated from fraction IE3-2 (**Additional file 3: Fig. S6a)**; five sub-fractions, IE3-2G1 (1.64 mg/mL), IE3-2G2 (2.53 mg/mL), IE3-2G3 (22.04 mg/mL), IE3-2G4 (1.83 mg/mL), and IE3-2G5 (0.22 mg/mL) were isolated from fraction IE3-2 (**Additional file 3: Fig. S6b).** To investigate the active ingredient, all these 26 sub-fractions (10 μg/mL) were co-cultured with mouse BMDC. Sub-fraction IE3-3G1 was selected by its highest immunomodulatory ability of upregulating IL-12p40 and PPARγ expression in mouse BMDC (**Fig. 1a, b**).
