# Supplementary material for: Moonlighting glyceraldehyde-3-phosphate dehydrogenase (GAPDH) protein of Lactobacillus gasseri attenuates allergic asthma via immunometabolic change in macrophages
Source: J Biomed Sci. 2022 Sep 29;29:75. doi: 10.1186/s12929-022-00861-8 (PMC9520948; doi:10.1186/s12929-022-00861-8)
Supplement: Supplementary file 2 — Additional file 2. Details of RNA-seq analysis. [file 12929_2022_861_MOESM2_ESM.docx]

**Additional file 2: Details of RNA-seq analysis**

Differentiated BMDM from 6-to 8-week-old C57BL/J mice were pre-cultured with 10 μg/mL LGp40 or CDp40 for 6 hours, and then re-stimulated with 100 μg/mL HDM for another 6 hours. Thereafter, harvested cells were washed twice with sterile PBS, and were then directly prepared for cDNA amplification and RNA-Seq library construction. Total RNA was extracted using Trizol® Reagent (Invitrogen, Waltham, MA, USA), according to the instructions’ manual. All RNA sample preparation procedures were carried out according to the official Illumina protocol (San Diego, CA, USA). SureSelect strand-specific RNA library preparation kit (Agilent, Santa Clara, CA, USA) was used for library construction followed by AMPure XP beads size selection (Beckman, Brea, CA, USA). The sequence was directly determined using Illumina’s sequencing-by-synthesis technology. Sequencing data (FASTQ files) were generated by Welgene Biotech Co. (Taipei, Taiwan) based on Illumina’s base calling program bcl2fastq v2.2.0. The heat map was generated using Morpheus software (<https://software.broadinstitute.org/morpheus>).
