# Supplementary material for: Moonlighting glyceraldehyde-3-phosphate dehydrogenase (GAPDH) protein of Lactobacillus gasseri attenuates allergic asthma via immunometabolic change in macrophages
Source: J Biomed Sci. 2022 Sep 29;29:75. doi: 10.1186/s12929-022-00861-8 (PMC9520948; doi:10.1186/s12929-022-00861-8)
Supplement: Supplementary file 3 — Additional file 3: Table S1. Experimental protocol of ion-exchange chromatography. Table S2. Experimental protocol of size-exclusion chromatography. Table S3. Data collection and refinement statistics of the LGp40 crystal. Figure S1. IL-12p40 levels of crude extracts used to stimulate mouse BMDC. Figure S2. Ion-exchange chromatography of crude extracts on a DEAE-Sepharose Fast Flow column. Figure S3. IL-12p40 levels of IE1-1 to IE4-2 fractions (fractions from ion-exchange chromatography) used to stimulate mouse BMDC. Figure S4. Size-exclusion chromatography of fractions from crude extract IE1 on a Sephacryl S-300 HR column. Figure S5. Size-exclusion chromatography of fractions from crude extract IE2 on a Sephacryl S-300 HR column. Figure S6. Size-exclusion chromatography of fractions from crude extract IE3 on a Sephacryl S-300 HR column. Figure S7. Identification and purification of sub-fraction IE3-3G1. Figure S8. GAPDH derived from probiotics and pathogens are dissimilar. Figure S9. RNA-seq analysis showed differentially regulated gene expression between LGp40 and CDp40-stimulated BMDM. Figure S10. The plasminogen interaction and plasmin activation ability of LGp40 decreased when the GAPDH activity was lost. [file 12929_2022_861_MOESM3_ESM.docx]

**Additional file 3.**

**Table S1.** Experimental protocol of ion-exchange chromatography.

**Table S2.** Experimental protocol of size-exclusion chromatography.

**Table S3.** Data collection and refinement statistics of the LGp40 crystal.

**Figure S1.** IL-12p40 levels of crude extracts used to stimulate mouse BMDC.

**Figure S2.** Ion-exchange chromatography of crude extracts on a DEAE-Sepharose Fast Flow column.

**Figure S3.** IL-12p40 levels of IE1-1 to IE4-2 fractions (fractions from ion-exchange chromatography) used to stimulate mouse BMDC.

**Figure S4** Size-exclusion chromatography of fractions from crude extract IE1 on a Sephacryl S-300 HR column.

**Figure S5.** Size-exclusion chromatography of fractions from crude extract IE2 on a Sephacryl S-300 HR column.

**Figure S6.** Size-exclusion chromatography of fractions from crude extract IE3 on a Sephacryl S-300 HR column.

**Figure S7.** Identification and purification of sub-fraction IE3-3G1.

**Figure S8.** GAPDH derived from probiotics and pathogens are dissimilar.

**Figure S9.** RNA-seq analysis showed differentially regulated gene expression between LGp40 and CDp40-stimulated BMDM.

**Figure S10.** The plasminogen interaction and plasmin activation ability of LGp40 decreased when the GAPDH activity was lost.

**Table S1. Experimental protocol of ion-exchange chromatography.**

Buffer A: 50 mM Tris-HCl (pH 8.5); Buffer B: 1 M NaCl in 50 mM Tris-HCl (pH 8.5)

**Table S2. Experimental protocol of size-exclusion chromatography.**

Buffer A: 50 mM NaCl in 50 mM Tris-HCl (pH 9.5)

**Table S3.** **Data collection and refinement statistics of the LGp40 crystal.**

| Crystal | LGp40 (7WWW) |
| --- | --- |
| Data collection | |
| Radiation source | NSRRC BL13C1 |
| Wavelength (Å) | 0.97622 |
| Space group | *I*4_1_22 |
| Unit cell parameter | |
| a (Å) | 114.92 |
| b (Å) | 114.92 |
| c (Å) | 118.62 |
| Resolution (Å) | 30-1.88 (1.95-1.88)^a^ |
| Number of reflections | 32168 (3031) |
| Completeness (%) | 98.8 (94.8) |
| Redundancy | 12.0 (10.5) |
| *R*_merge_ (%) | 6.7 (54.6) |
| I/σ (I) | 41.7 (3.7) |
| Overall Wilson B factor (Å^2^) | 32.0 |
| Refinement | |
| Resolution | 29.7-1.88 (1.98-1.88) |
| Reflections (work) | 30413 (4180) |
| Reflections (free) | 1625 (240) |
| *R*_work_ (%) | 13.4 (20.3) |
| R_free_ (%) | 21.7 (31.3) |
| Geometry deviations |  |
| Bond length (Å) | 0.007 |
| Bond angles (^o^) | 1.4 |
| Mean B-values (Å) / No. |  |
| Protein atoms | 44.2 / 2534 |
| Water molecules | 52.5 / 247 |
| Ligand atoms | 39.8 / 48 |
| Ramachandran plot (%)^b^ |  |
| Favored region | 97.6 |
| Allowed region | 2.4 |

^a^Values in the parentheses are for the highest resolution shells.

^b^Categories were defined by RAMPAGE.

**
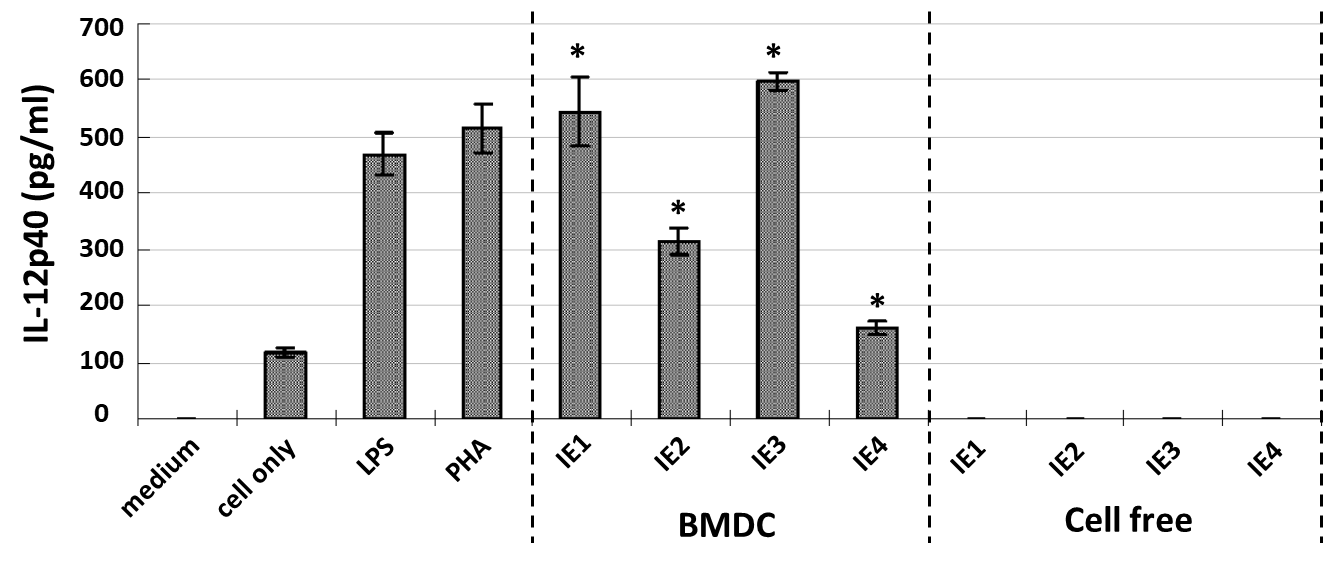
**

**Fig. S1 IL-12p40 levels of crude extracts used to stimulate mouse BMDC.**

AS 0-25% precipitates (IE1), AS 25-50% precipitates (IE2), AS 50-75% precipitates (IE3), and AS 75-100% precipitates (IE4). (n = 3, *p < 0.05 compared to cell only group, one-way ANOVA with Bonferroni multiple comparison test.)

**
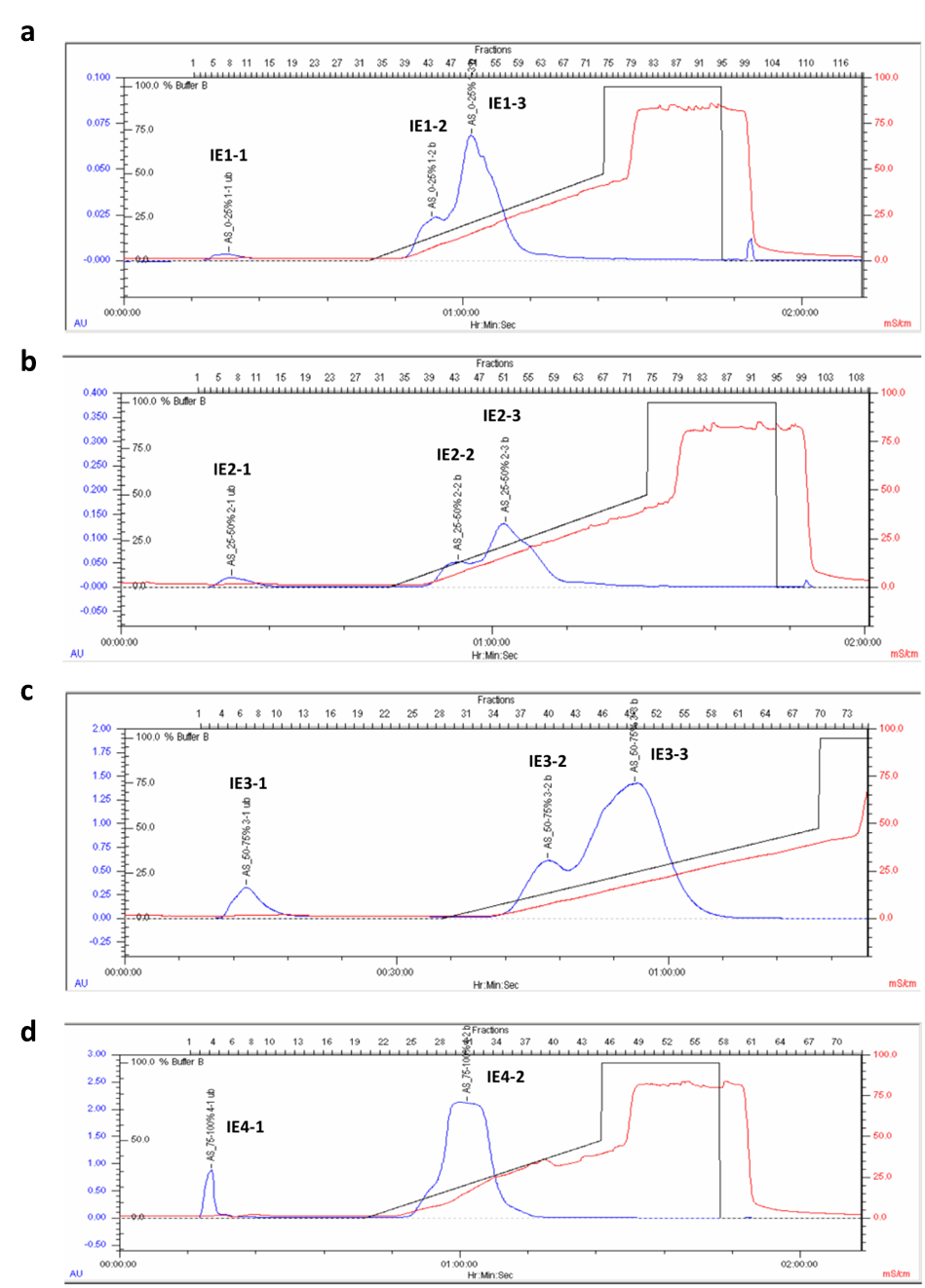
**

**Fig. S2 Ion-exchange chromatography of crude extracts on a DEAE-Sepharose Fast Flow column.**

The curve of elution in DEAE Sepharose Fast Flow chromatography. Samples were **a** AS 0-25% precipitates (IE1), **b** AS 25-50% precipitates (IE2), **c** AS 50-75% precipitates (IE3), and **d** AS 75-100% precipitates (IE4). Starting chromatography buffer (buffer A): 50 mM Tris-HCl (pH 8.5). Elution buffer (buffer B): 1 M NaCl in starting buffer. Elution was a linear gradient (from 0 to 1 M) of NaCl in Tris-HCl buffer. Detection wavelength: UV 280 nm; Flow rate: 1.2 ml/min; Collection rate: 1 ml/tube.

**
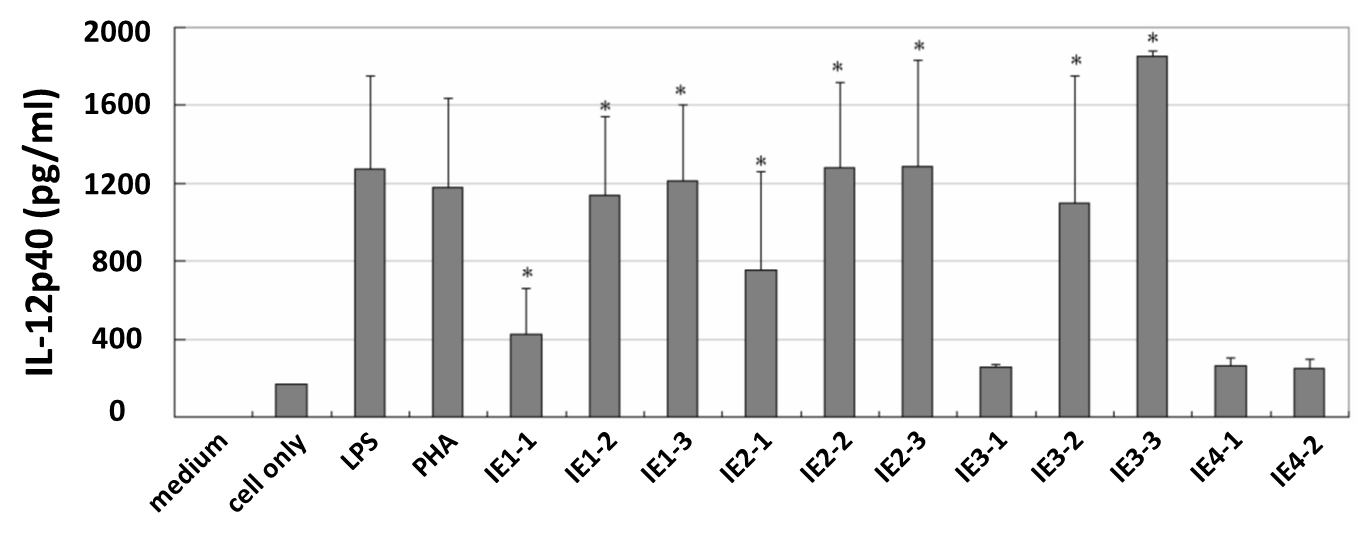
**

**Fig. S3 IL-12p40 levels of IE1-1 to IE4-2 fractions (fractions from ion-exchange chromatography) used to stimulate mouse BMDC.**

n = 3, *p < 0.05 compared to cell only group, one-way ANOVA with Bonferroni multiple comparison test.

**
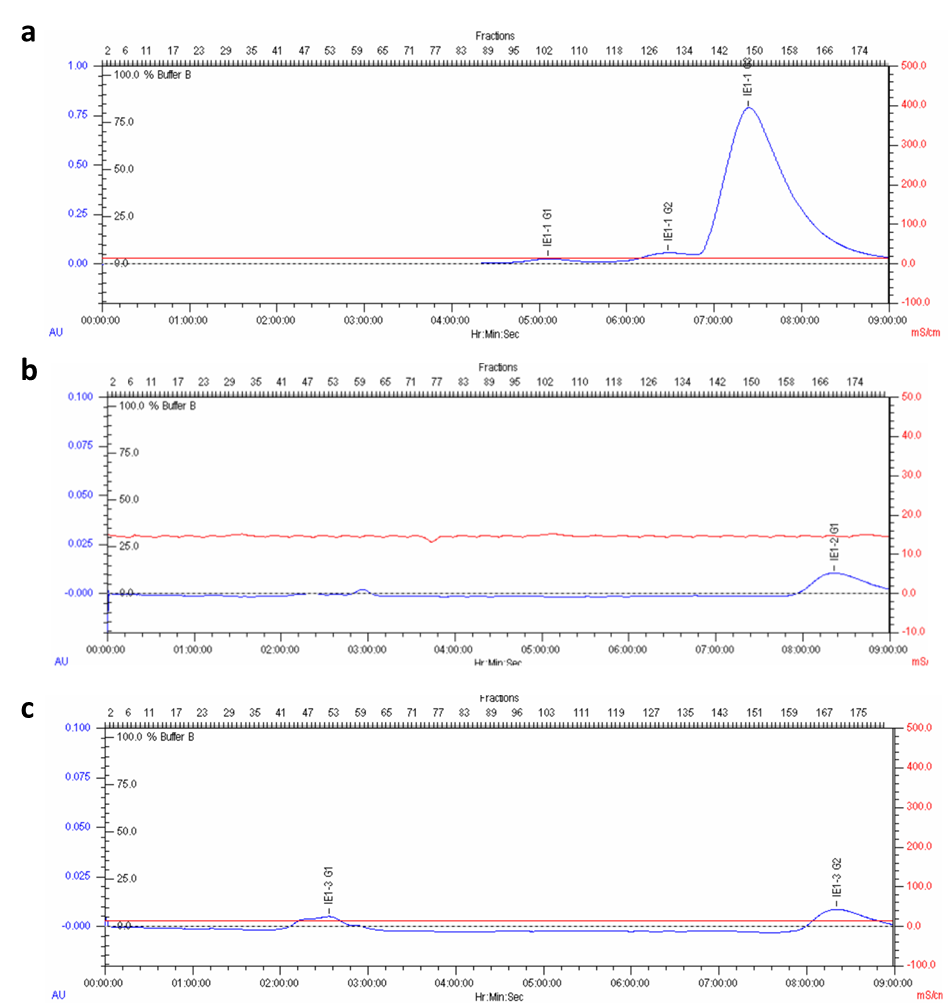
**

**Fig. S4 Size-exclusion chromatography of fractions from crude extract IE1 on a Sephacryl S-300 HR column.**

The curve of elution in Sephacryl S-300 HR chromatography. Samples were fraction **a** IE1-1, **b** IE1-2, and **c** IE1-3 from DEAE-Sepharose Fast Flow column. Equilibration and elution buffer (buffer A): 50 mM NaCl in 50 mM Tris-HCl (pH 9.5). Detection wavelength: UV 280 nm; Flow rate: 0.5 ml/min; Collection rate: 1 ml/tube.

**
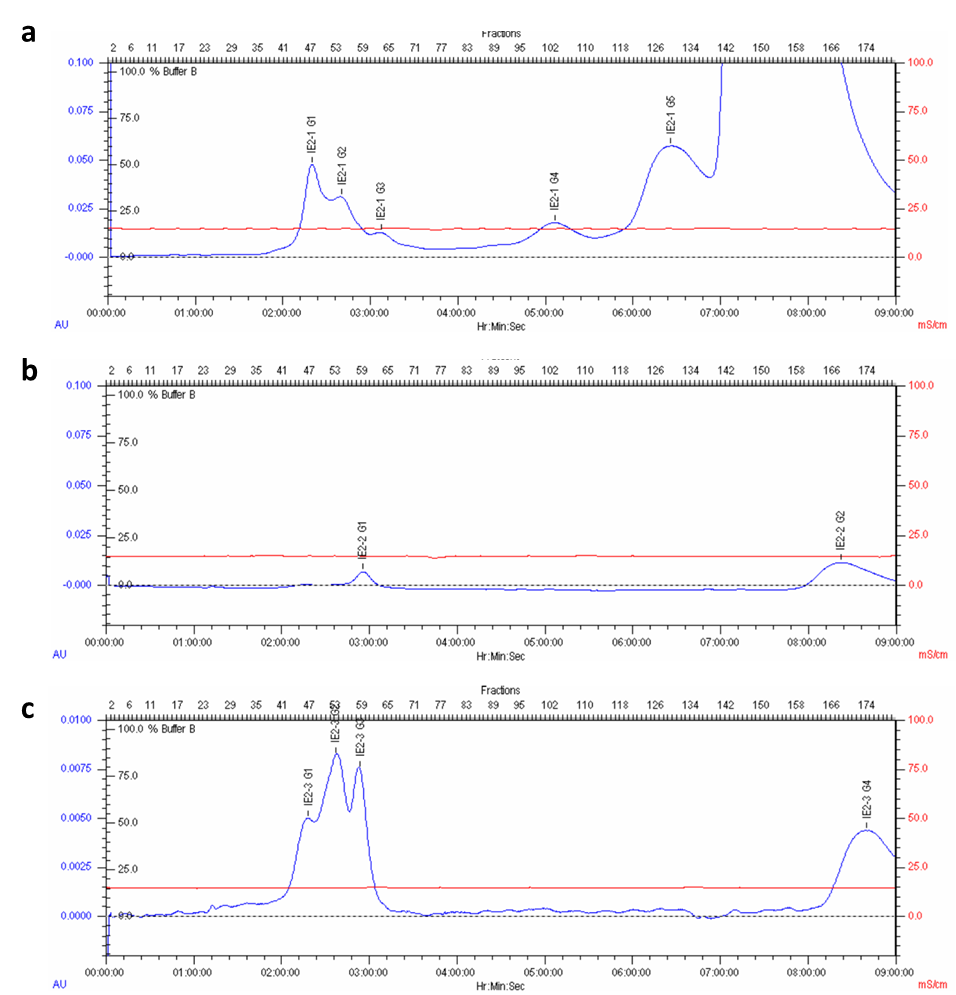
**

**Fig. S5 Size-exclusion chromatography of fractions from crude extract IE2 on a Sephacryl S-300 HR column.**

The curve of elution in Sephacryl S-300 HR chromatography. Samples were fraction **a** IE2-1, **b** IE2-2, and **c** IE2-3 from DEAE-Sepharose Fast Flow column. Equilibration and elution buffer (buffer A): 50 mM NaCl in 50 mM Tris-HCl (pH 9.5). Detection wavelength: UV 280 nm; Flow rate: 0.5 mL/min; Collection rate: 1 mL/tube.

**
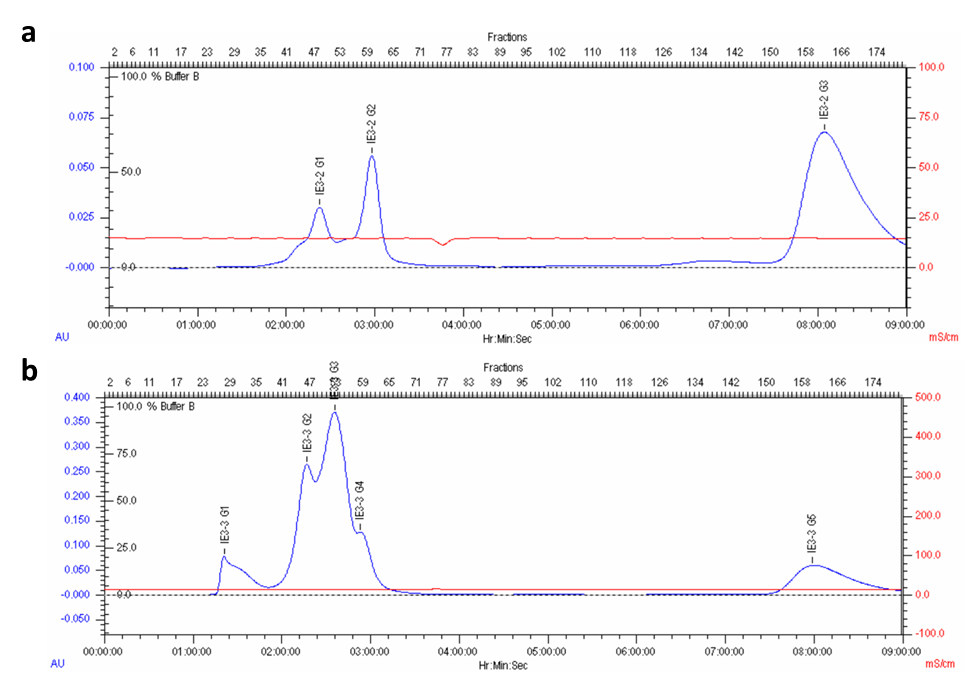
**

**Fig. S6 Size-exclusion chromatography of fractions from crude extract IE3 on a Sephacryl S-300 HR column.**

The curve of elution in Sephacryl S-300 HR chromatography. Samples were fraction **a** IE3-2 and **b** IE3-3 from DEAE-Sepharose Fast Flow column. Equilibration and elution buffer (buffer A): 50 mM NaCl in 50 mM Tris-HCl (pH 9.5). Detection wavelength: UV 280 nm; Flow rate: 0.5 mL/min; Collection rate: 1 mL/tube.

**
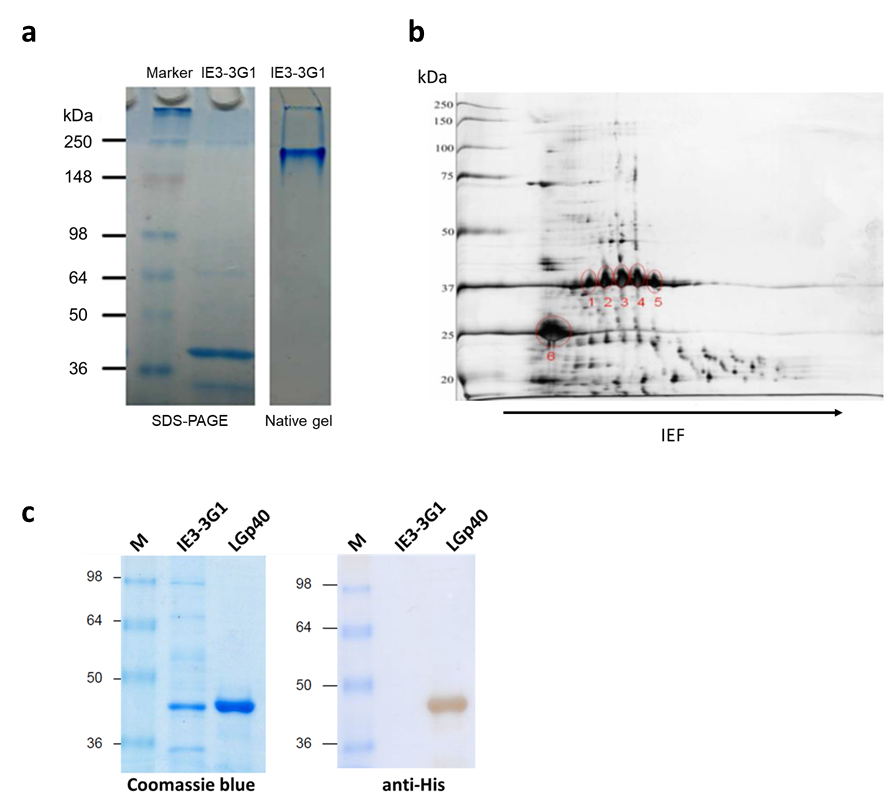
**

**Fig. S7 Identification and purification of sub-fraction IE3-3G1.**

**a** Sub-fraction IE3-3G1 was subjected to electrophoresis on a 10% SDS-PAGE gel and a native gel. **b** 2-DE separation of proteins from fraction IE3-3G1. **c** Sub-fraction IE3-3G1 and recombinant LGp40 protein were subjected to electrophoresis on a 10% SDS-PAGE gel and were detected by His-tag antibody.

**
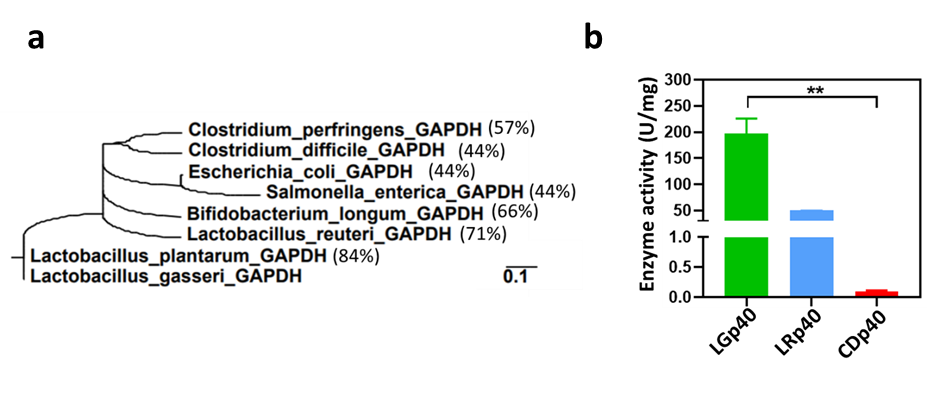
Fig. S8** **GAPDH derived from probiotic and pathogen are dissimilar.**

**a** Phylogenetic relationship of the GAPDH. **b** Enzyme activity of recombinant proteins (n= 3-6, **p < 0.01, one-way ANOVA with Bonferroni multiple comparison test).

**
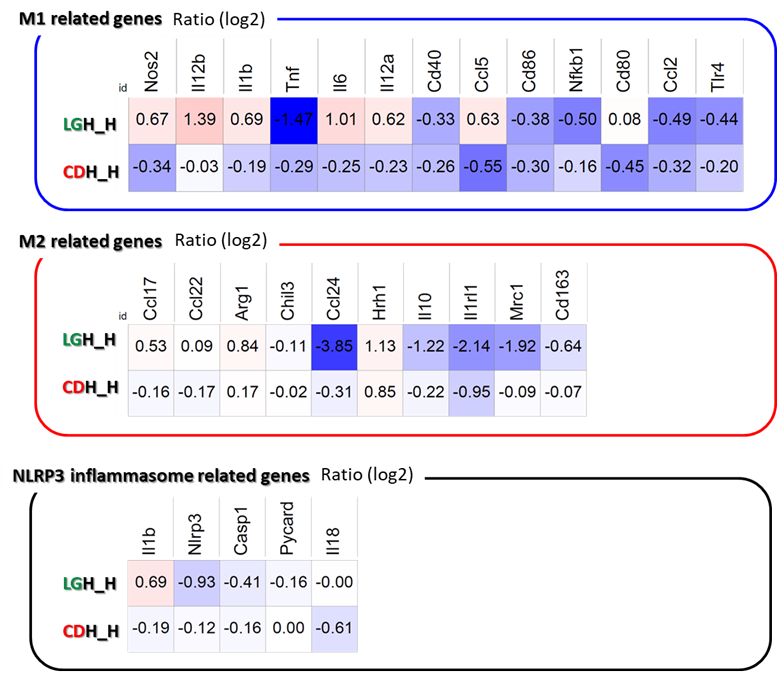
Fig. S9 RNA-seq analysis showed differentially regulated gene expression between LGp40- and CDp40-stimulated BMDM.**

BMDM were pre-incubated with LGp40 and CDp40 and then re-stimulated with HDM. Afterward, these cells were harvested and ran the RNA-seq analysis.


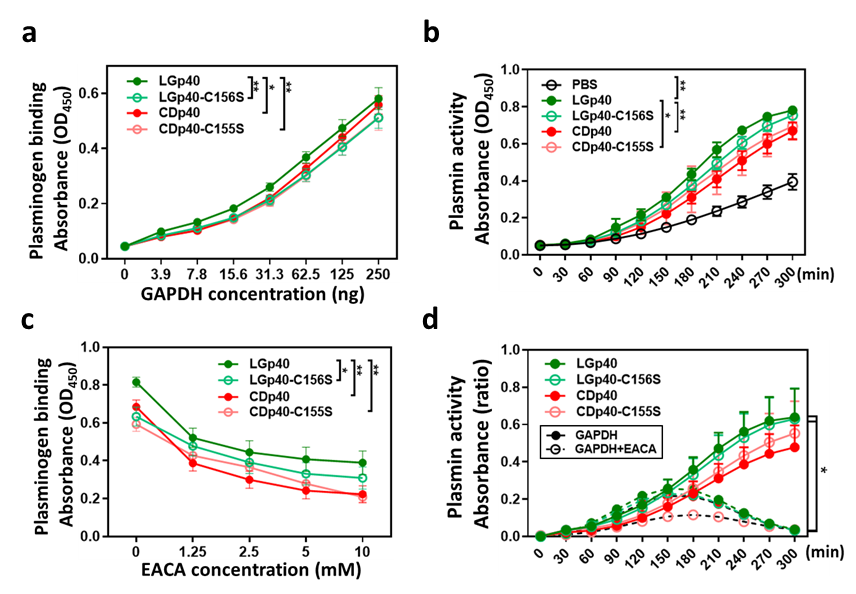
**Fig. S10 The plasminogen interaction and plasmin activation ability of LGp40 decreased when the GAPDH activity was lost.**

**a** Increasing concentration of recombinant proteins (3.9 to 250 ng) interact with plasminogen in vitro. **b** Kinetic changes of uPA-mediated plasmin activation by the recombinant proteins. **c** Binding of recombinant proteins to plasminogen in presence of increasing concentration of the lysine analog EACA (1.25 to 10mM) was measured by ELISA. **d** Kinetic changes of uPA-mediated plasmin activation by the recombinant proteins in presence of EACA (Ration with PBS). Experiment was repeated 3 times (*p < 0.05 and **p < 0.01, two-way ANOVA with Bonferroni multiple comparison test).
