# Supplementary material for: Moonlighting glyceraldehyde-3-phosphate dehydrogenase (GAPDH) protein of Lactobacillus gasseri attenuates allergic asthma via immunometabolic change in macrophages
Source: J Biomed Sci. 2022 Sep 29;29:75. doi: 10.1186/s12929-022-00861-8 (PMC9520948; doi:10.1186/s12929-022-00861-8)
Supplement: Supplementary file 4 — Additional file 4. Raw data of sub-fraction IE3-3G1 proteomics analysis using LC–MS/MS. [file 12929_2022_861_MOESM4_ESM.pdf]

Additional file 4. Raw data of sub-fraction IE3-3G1 proteomics analysis using LC-MS/MS.

(1-1) Peptide Summary Report (protein spot 1, IE-1) Bacteria (Eubacteria)

Mascot Search Results

**User**	**: liaopc**
**Email**	**:**
**Search title**	**: IE-1**
**MS data file**	**: merge.txt**
**Database**	**: NCBInr 090616 (9069431 sequences; 3106408609 residues)**
**Taxonomy**	**: Bacteria (Eubacteria) (4853765 sequences)**
**Timestamp**	**: 9 Jan 2012 at 09:15:28 GMT**
**Protein hits**	: gi|42518797 glyceraldehyde 3-phosphate dehydrogenase [Lactobacillus johnsonii NCC 533]
	gi|227361679 glyceraldehyde-3-phosphate dehydrogenase [Lactobacillus sakei subsp. carnosus DSM 15831]
	gi|227525966 glyceraldehyde-3-phosphate dehydrogenase (phosphorylating) [Lactobacillus jensenii JV-V16]
	gi|116492261 glyceraldehyde-3-phosphate dehydrogenase [Pediococcus pentosaceus ATCC 25745]
	gi|170016655 glyceraldehyde-3-phosphate dehydrogenase/erythrose-4-phosphate dehydrogenase [Leuconostoc citreum KM20]
	gi|158334876 hypothetical protein AM1_1713 [Acaryochloris marina MBIC11017]
	gi|148266124 hypothetical protein Gura_4114 [Geobacter uraniireducens Rf4]
	gi|217077222 chromosome segregation SMC protein, putative [Thermosipho africanus TCF52B]
	gi|20799584 glyceraldehyde-3-phosphate dehydrogenase [Staphylococcus arlettae]
	gi|182412978 excinuclease ABC subunit C [Opitutus terrae PB90-1]
	gi|42561108 holo-[acyl-carrier-protein] synthase [Mycoplasma mycoides subsp. mycoides SC str. PG1]
	gi|51244876 Ntr family two-component response regulator [Desulfotalea psychrophila LSv54]
	gi|172057364 hypothetical protein Exig_1335 [Exiguobacterium sibiricum 255-15]
	gi|227424225 biopolymer transport protein [Denitrovibrio acetiphilus DSM 12809]
	gi|72382545 fused serine peptidase/N-terminal uncharacterized domain protein [Prochlorococcus marinus str. NATL2A]
	gi|237751407 glycosyl transferase [Helicobacter bilis ATCC 43879]
	gi|123965634 putative deoxyribodipyrimidine photolyase [Prochlorococcus marinus str. MIT 9515]
	gi|24213647 polynucleotide phosphorylase/polyadenylase [Leptospira interrogans serovar Lai str. 56601]
	gi|84385691 ISTde1, transposase [Vibrio splendidus 12B01]
	gi|88805502 putative two-component system sensor kinase/response regulator fusion protein [Robiginitalea biformata H'
	gi|15924260 ribosome-binding factor A [Staphylococcus aureus subsp. aureus Mu50]
	gi|1706361 RecName: Full=Transcriptional regulatory protein degU
	gi|223483524 single-strand binding protein [Brevundimonas sp. BAL3]
	gi|86131594 putative SpoU rRNA methylase family protein [Cellulophaga sp. MED134]
	gi|172058506 diguanylate cyclase/phosphodiesterase with PAS/PAC sensor(s) [Exiguobacterium sibiricum 255-15]
	gi|110005394 putative atpase with chaperone activity, clp protease subunit protein [Spiroplasma citri]
	gi|150016208 trigger factor [Clostridium beijerinckii NCIMB 8052]
	gi|224534714 UDP-N-acetylglucosamine 1-carboxyvinyltransferase [Borrelia spielmanii A14S]
	gi|153811501 hypothetical protein RUMOBE_01893 [Ruminococcus obeum ATCC 29174]
	gi|85715004 aldehyde oxidase and xanthine dehydrogenase [Nitrobacter sp. Nb-311A]
	gi|84494635 putative glycerol phosphate dehydrogenase [Janibacter sp. HTCC2649]
	gi|160894823 hypothetical protein CLOL250_02373 [Clostridium sp. L2-50]
	gi|114568029 hypothetical protein Swol_2524 [Syntrophomonas wolfei subsp. wolfei str. Goettingen]

Probability Based Mowse Score

Ions score is -10*Log(P), where P is the probability that the observed match is a random event.
Individual ions scores > 55 indicate identity or extensive homology (p<0.05).
Protein scores are derived from ions scores as a non-probabilistic basis for ranking protein hits.

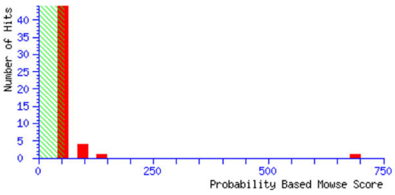

Peptide Summary Report

Format As | Peptide Summary | Help

Significance threshold p< 0.05 | Max. number of hits AUTO

Standard scoring ◉ MudPIT scoring ○ | Ions score or expect cut-off 0 | Show sub-sets 0

Show pop-ups ◉ Suppress pop-ups ○ | Sort unassigned Decreasing Score | Require bold red ☐

(1-2) Peptide Summary Report (protein spot 1, IE-1) Firmicutes (gram-positive bacteria)

Mascot Search Results

**User**	: liaopc
**Email**	:
**Search title**	: IE-1
**MS data file**	: merge.txt
**Database**	: NCBInr 090616 (9069431 sequences; 3106408609 residues)
**Taxonomy**	: Firmicutes (gram-positive bacteria) (1006590 sequences)
**Timestamp**	: 10 Jan 2012 at 08:20:50 GMT
**Protein hits**	: gi|42518797 glyceraldehyde 3-phosphate dehydrogenase [Lactobacillus johnsonii NCC 533]
	gi|227361679 glyceraldehyde-3-phosphate dehydrogenase [Lactobacillus sakei subsp. carnosus DSM 15831]
	gi|227525966 glyceraldehyde-3-phosphate dehydrogenase (phosphorylating) [Lactobacillus jensenii JV-V16]
	gi|172057364 hypothetical protein Exig_1335 [Exiguobacterium sibiricum 255-15]
	gi|229004124 hypothetical protein bmyco0002_10890 [Bacillus mycoides Rock1-4]
	gi|154502799 hypothetical protein RUMGNA_00613 [Ruminococcus gnavus ATCC 29149]
	gi|20799584 glyceraldehyde-3-phosphate dehydrogenase [Staphylococcus arlettae]
	gi|197301564 hypothetical protein RUMLAC_00290 [Ruminococcus lactaris ATCC 29176]
	gi|116492261 glyceraldehyde-3-phosphate dehydrogenase [Pediococcus pentosaceus ATCC 25745]
	gi|149181561 hypothetical protein BSG1_13616 [Bacillus sp. SG-1]
	gi|150016208 trigger factor [Clostridium beijerinckii NCIMB 8052]
	gi|1706361 RecName: Full=Transcriptional regulatory protein degU
	gi|15924260 ribosome-binding factor A [Staphylococcus aureus subsp. aureus Mu50]
	gi|228918843 hypothetical protein bthur0012_59520 [Bacillus thuringiensis serovar pulsiensis BGSC 4CC1]
	gi|170016655 glyceraldehyde-3-phosphate dehydrogenase/erythrose-4-phosphate dehydrogenase [Leuconostoc citreum KM20]
	gi|228948651 Cell surface protein [Bacillus thuringiensis serovar monterrey BGSC 4AJ1]
	gi|172058506 diguanylate cyclase/phosphodiesterase with PAS/PAC sensor(s) [Exiguobacterium sibiricum 255-15]
	gi|218273468 hypothetical protein ROSINTL182_01355 [Roseburia intestinalis L1-82]
	gi|150388754 phosphoribosylglycinamide formyltransferase [Alkaliphilus metalliredigens QYMF]
	gi|153811501 hypothetical protein RUMOBE_01893 [Ruminococcus obeum ATCC 29174]
	gi|229229306 ATPase component of various ABC-type transport systems with duplicated ATPase domain [Desulfotomaculum a
	gi|238927776 conserved hypothetical protein [Selenomonas flueggei ATCC 43531]
	gi|160894823 hypothetical protein CLOL250_02373 [Clostridium sp. L2-50]
	gi|30260965 hypothetical protein BA0824 [Bacillus anthracis str. Ames]
	gi|29376021 terminase, small subunit, internal deletion [Enterococcus faecalis V583]
	gi|229827478 hypothetical protein GCWU000182_02867 [Abiotrophia defectiva ATCC 49176]
	gi|226323766 hypothetical protein COPCOM_01541 [Coprococcus comes ATCC 27758]
	gi|89897039 hypothetical protein DSY4293 [Desulfitobacterium hafniense Y51]
	gi|126700567 putative phage anti-repressor [Clostridium difficile 630]
	gi|116510863 transcriptional antiterminator [Lactococcus lactis subsp. cremoris SK11]
	gi|150391186 YheD [Alkaliphilus metalliredigens QYMF]
	gi|199598725 Chaperone ClpB [Lactobacillus rhamnosus HN001]
	gi|20808539 O-acetylhomoserine sulfhydrylase [Thermoanaerobacter tengcongensis MB4]
	gi|42519394 oligopeptide ABC transporter solute-binding component [Lactobacillus johnsonii NCC 533]
	gi|125973701 Alpha/beta hydrolase fold-3 [Clostridium thermocellum ATCC 27405]
	gi|167465604 possible phospholipase [Paenibacillus larvae subsp. larvae BRL-230010]
	gi|167750765 hypothetical protein EUBSIR_01743 [Eubacterium siraeum DSM 15702]
	gi|226313151 hypothetical protein BBR47_35640 [Brevibacillus brevis NBRC 100599]
	gi|229917100 nucleotide sugar dehydrogenase [Exiguobacterium sp. AT1b]
	gi|56961831 lysine decarboxylase [Bacillus clausii KSM-K16]
	gi|134301021 phenylacetate--CoA ligase [Desulfotomaculum reducens MI-1]
	gi|153953104 recombinase [Clostridium kluyveri DSM 555]

Probability Based Mowse Score

Ions score is -10*Log(P), where P is the probability that the observed match is a random event.
Individual ions scores > 52 indicate identity or extensive homology (p<0.05).
Protein scores are derived from ions scores as a non-probabilistic basis for ranking protein hits.

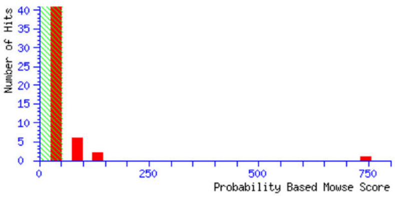

Peptide Summary Report

Format As [Peptide Summary] Help

Significance threshold p< 0.05 Max. number of hits AUTO

Standard scoring ○ MudPIT scoring ◉ Ions score or expect cut-off 0 Show sub-sets 0

Show pop-ups ◉ Suppress pop-ups ○ Sort unassigned [Decreasing Score] Require bold red ☐

(1-3) Peptide Summary Report (protein spot 1, IE-1) Other Firmicutes

Mascot Search Results

User	:	liaopc
Email	:	
Search title	:	IE-1
MS data file	:	merge.txt
Database	:	NCBInr 090616 (9069431 sequences; 3106408609 residues)
Taxonomy	:	Other Firmicutes (967613 sequences)
Timestamp	:	10 Jan 2012 at 08:33:58 GMT
Protein hits	:	gi|42518797 glyceraldehyde 3-phosphate dehydrogenase [Lactobacillus johnsonii NCC 533]
		gi|227361679 glyceraldehyde-3-phosphate dehydrogenase [Lactobacillus sakei subsp. carnosus DSM 15831]
		gi|227525966 glyceraldehyde-3-phosphate dehydrogenase (phosphorylating) [Lactobacillus jensenii JV-V16]
		gi|172057364 hypothetical protein Exig_1335 [Exiguobacterium sibiricum 255-15]
		gi|229004124 hypothetical protein bmyco0002_10890 [Bacillus mycoides Rock1-4]
		gi|154502799 hypothetical protein RUMGNA_00613 [Ruminococcus gnavus ATCC 29149]
		gi|20799584 glyceraldehyde-3-phosphate dehydrogenase [Staphylococcus arlettae]
		gi|197301564 hypothetical protein RUMLAC_00290 [Ruminococcus lactaris ATCC 29176]
		gi|116492261 glyceraldehyde-3-phosphate dehydrogenase [Pediococcus pentosaceus ATCC 25745]
		gi|149181561 hypothetical protein BSG1_13616 [Bacillus sp. SG-1]
		gi|150016208 trigger factor [Clostridium beijerinckii NCIMB 8052]
		gi|1706361 RecName: Full=Transcriptional regulatory protein degU
		gi|15924260 ribosome-binding factor A [Staphylococcus aureus subsp. aureus Mu50]
		gi|228918843 hypothetical protein bthur0012_59520 [Bacillus thuringiensis serovar pulsiensis BGSC 4CC1]
		gi|170016655 glyceraldehyde-3-phosphate dehydrogenase/erythrose-4-phosphate dehydrogenase [Leuconostoc citreum KM20]
		gi|228948651 Cell surface protein [Bacillus thuringiensis serovar monterrey BGSC 4AJ1]
		gi|172058506 diguanylate cyclase/phosphodiesterase with PAS/PAC sensor(s) [Exiguobacterium sibiricum 255-15]
		gi|218273468 hypothetical protein ROSINTL182_01355 [Roseburia intestinalis L1-82]
		gi|150388754 phosphoribosylglycinamide formyltransferase [Alkaliphilus metalliredigens QYMF]
		gi|153811501 hypothetical protein RUMOBE_01893 [Ruminococcus obeum ATCC 29174]
		gi|229229306 ATPase component of various ABC-type transport systems with duplicated ATPase domain [Desulfotomaculum a
		gi|238927776 conserved hypothetical protein [Selenomonas flueggei ATCC 43531]
		gi|160894823 hypothetical protein CLOL250_02373 [Clostridium sp. L2-50]
		gi|30260965 hypothetical protein BA0824 [Bacillus anthracis str. Ames]
		gi|29376021 terminase, small subunit, internal deletion [Enterococcus faecalis V583]
		gi|229827478 hypothetical protein GCWU000182_02867 [Abiotrophia defectiva ATCC 49176]
		gi|226323766 hypothetical protein COPCOM_01541 [Coprococcus comes ATCC 27758]
		gi|89897039 hypothetical protein DSY4293 [Desulfitobacterium hafniense Y51]
		gi|126700567 putative phage anti-repressor [Clostridium difficile 630]
		gi|116510863 transcriptional antiterminator [Lactococcus lactis subsp. cremoris SK11]
		gi|150391186 YheD [Alkaliphilus metalliredigens QYMF]
		gi|199598725 Chaperone ClpB [Lactobacillus rhamnosus HN001]
		gi|20808539 O-acetylhomoserine sulfhydrylase [Thermoanaerobacter tengcongensis MB4]
		gi|42519394 oligopeptide ABC transporter solute-binding component [Lactobacillus johnsonii NCC 533]
		gi|125973701 Alpha/beta hydrolase fold-3 [Clostridium thermocellum ATCC 27405]
		gi|167465604 possible phospholipase [Paenibacillus larvae subsp. larvae BRL-230010]
		gi|167750765 hypothetical protein EUBSIR_01743 [Eubacterium siraeum DSM 15702]
		gi|226313151 hypothetical protein BBR47_35640 [Brevibacillus brevis NBRC 100599]
		gi|229917100 nucleotide sugar dehydrogenase [Exiguobacterium sp. AT1b]
		gi|56961831 lysine decarboxylase [Bacillus clausii KSM-K16]
		gi|134301021 phenylacetate--CoA ligase [Desulfotomaculum reducens MI-1]
		gi|153953104 recombinase [Clostridium kluyveri DSM 555]

Probability Based Mowse Score

Ions score is -10*Log(P), where P is the probability that the observed match is a random event.
Individual ions scores > 52 indicate identity or extensive homology (p<0.05).
Protein scores are derived from ions scores as a non-probabilistic basis for ranking protein hits.

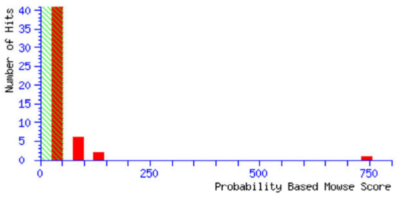

Peptide Summary Report

Format As [Peptide Summary] Help

Significance threshold p< [0.05] Max. number of hits [AUTO]

Standard scoring ○ MudPIT scoring ◉ Ions score or expect cut-off [0] Show sub-sets [0]

Show pop-ups ◉ Suppress pop-ups ○ Sort unassigned [Decreasing Score] Require bold red ☐

(2-1) Peptide Summary Report (protein spot 2, IE-2) Bacteria (Eubacteria)

Mascot Search Results

User : liaopc
Email :
Search title : IE-2
MS data file : merge.txt
Database : NCBInr 090616 (9069431 sequences; 3106408609 residues)
Taxonomy : Bacteria (Eubacteria) (4853765 sequences)
Timestamp : 10 Jan 2012 at 01:19:37 GMT
Protein hits : gi|42518797 glyceraldehyde 3-phosphate dehydrogenase [Lactobacillus johnsonii NCC 533]
gi|227520527 glyceraldehyde-3-phosphate dehydrogenase, phosphorylating [Lactobacillus gasseri JV-V03]
gi|227361679 glyceraldehyde-3-phosphate dehydrogenase [Lactobacillus sakei subsp. carnosus DSM 15831]
gi|227525966 glyceraldehyde-3-phosphate dehydrogenase (phosphorylating) [Lactobacillus jensenii JV-V16]
gi|111610131 glyceraldehyde 3-phosphate dehydrogenase [Lactobacillus helveticus CNRZ32]
gi|90962141 glyceraldehyde 3-phosphate dehydrogenase [Lactobacillus salivarius UCC118]
gi|116492261 glyceraldehyde-3-phosphate dehydrogenase [Pediococcus pentosaceus ATCC 25745]
gi|170016655 glyceraldehyde-3-phosphate dehydrogenase/erythrose-4-phosphate dehydrogenase [Leuconostoc citreum KM20]
gi|229233449 dehydrogenase of unknown specificity similar to short-chain alcohol dehydrogenase [Chitinophaga pinensis
gi|94499337 hypothetical protein RED65_11124 [Oceanobacter sp. RED65]
gi|225103805 glyceraldehyde-3-phosphate dehydrogenase, type I [Methylophaga sp. DMS010]
gi|237751407 glycosyl transferase [Helicobacter bilis ATCC 43879]
gi|117921863 prepilin-type cleavage/methylation-like protein [Shewanella sp. ANA-3]
gi|148553294 beta-lactamase [Sphingomonas wittichii RW1]
gi|111022069 non-ribosomal peptide synthetase [Rhodococcus jostii RHA1]
gi|228577910 pilus biogenesis protein [Citrobacter sp. 30_2]
gi|197762281 predicted protein [Streptomyces sp. SPB74]
gi|167764151 hypothetical protein BACSTE_02535 [Bacteroides stercoris ATCC 43183]
gi|148266124 hypothetical protein Gura_4114 [Geobacter uraniireducens Rf4]
gi|1706361 RecName: Full=Transcriptional regulatory protein degU
gi|118473519 thiocyanate hydrolase beta subunit [Mycobacterium smegmatis str. MC2 155]
gi|20799584 glyceraldehyde-3-phosphate dehydrogenase [Staphylococcus arlettae]
gi|227424225 biopolymer transport protein [Denitrovibrio acetiphilus DSM 12809]
gi|239616934 amidohydrolase [Kosmotoga olearia TBF 19.5.1]
gi|224391617 cobalamin biosynthesis protein CobT [Candidatus Pelagibacter sp. HTCC7211]
gi|227500542 DNA-binding protein [Anaerococcus tetradius ATCC 35098]
gi|42561108 holo-[acyl-carrier-protein] synthase [Mycoplasma mycoides subsp. mycoides SC str. PG1]
gi|229175622 Cell surface protein [Bacillus cereus MM3]
gi|150016208 trigger factor [Clostridium beijerinckii NCIMB 8052]
gi|229556841 glycosyltransferase [Listeria grayi DSM 20601]
gi|196228419 hypothetical protein CfE428DRAFT_0450 [Chthoniobacter flavus Ellin428]
gi|57234620 glyceraldehyde-3-phosphate dehydrogenase, type I [Dehalococcoides ethenogenes 195]
gi|158337485 ATP-dependent metalloprotease FtsH-like protein [Acaryochloris marina MBIC11017]
gi|150385193 Fibronectin, type III domain protein [Victivallis vadensis ATCC BAA-548]
gi|146295492 hypothetical protein Csac_0434 [Caldicellulosiruptor saccharolyticus DSM 8903]
gi|197301564 hypothetical protein RUMLAC_00290 [Ruminococcus lactaris ATCC 29176]
gi|227979847 putative proteasome component/protein of unknown function, DUF275 [Tsukamurella paurometabola DSM 20162]
gi|20799602 glyceraldehyde-3-phosphate dehydrogenase [Staphylococcus piscifermentans]
gi|171473203 glyceraldehyde-3-phosphate dehydrogenase [Candidatus Phytoplasma vitis]
gi|148380680 hydrolase [Clostridium botulinum A str. ATCC 3502]

Probability Based Mowse Score

Ions score is -10*Log(P), where P is the probability that the observed match is a random event.
Individual ions scores > 59 indicate identity or extensive homology (p<0.05).
Protein scores are derived from ions scores as a non-probabilistic basis for ranking protein hits.

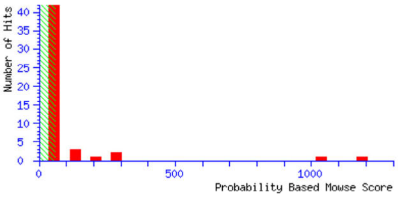

Peptide Summary Report

Format As | Peptide Summary | Help

Significance threshold p< 0.05 | Max. number of hits AUTO

Standard scoring ◉ MudPIT scoring ○ | Ions score or expect cut-off 0 | Show sub-sets 0

Show pop-ups ◉ Suppress pop-ups ○ | Sort unassigned Decreasing Score | Require bold red ☐

(2-2) Peptide Summary Report (protein spot 2, IE-2) Firmicutes (gram-positive bacteria)

MATRIX SCIENCE Mascot Search Results

User : liaopc
Email :
Search title : IE-2
MS data file : merge.txt
Database : NCBInr 090616 (9069431 sequences; 3106408609 residues)
Taxonomy : Firmicutes (gram-positive bacteria) (1006590 sequences)
Timestamp : 10 Jan 2012 at 09:18:16 GMT
Protein hits :

gi|42518797 glyceraldehyde 3-phosphate dehydrogenase [Lactobacillus johnsonii NCC 533]
gi|227520527 glyceraldehyde-3-phosphate dehydrogenase, phosphorylating [Lactobacillus gasseri JV-V03]
gi|227361679 glyceraldehyde-3-phosphate dehydrogenase [Lactobacillus sakei subsp. carnosus DSM 15831]
gi|227525966 glyceraldehyde-3-phosphate dehydrogenase (phosphorylating) [Lactobacillus jensenii JV-V16]
gi|90962141 glyceraldehyde 3-phosphate dehydrogenase [Lactobacillus salivarius UCC118]
gi|20799584 glyceraldehyde-3-phosphate dehydrogenase [Staphylococcus arlettae]
gi|227500542 DNA-binding protein [Anaerococcus tetradius ATCC 35098]
gi|148380680 hydrolase [Clostridium botulinum A str. ATCC 3502]
gi|197301564 hypothetical protein RUMLAC_00290 [Ruminococcus lactaris ATCC 29176]
gi|116492261 glyceraldehyde-3-phosphate dehydrogenase [Pediococcus pentosaceus ATCC 25745]
gi|229828817 hypothetical protein GCWU000342_00900 [Shuttleworthia satelles DSM 14600]
gi|146295492 hypothetical protein Csac_0434 [Caldicellulosiruptor saccharolyticus DSM 8903]
gi|170016655 glyceraldehyde-3-phosphate dehydrogenase/erythrose-4-phosphate dehydrogenase [Leuconostoc citreum KM20]
gi|229175622 Cell surface protein [Bacillus cereus MM3]
gi|150016208 trigger factor [Clostridium beijerinckii NCIMB 8052]
gi|20799602 glyceraldehyde-3-phosphate dehydrogenase [Staphylococcus piscifermentans]
gi|1706361 RecName: Full=Transcriptional regulatory protein degU
gi|148378084 hypothetical protein CBO0076 [Clostridium botulinum A str. ATCC 3502]
gi|154502799 hypothetical protein RUMGNA_00613 [Ruminococcus gnavus ATCC 29149]
gi|228995199 Collagen adhesion protein [Bacillus pseudomycoides DSM 12442]
gi|228583035 trigger factor protein [Clostridium sp. 7_2_43FAA]
gi|149183693 hypothetical protein BSG1_13561 [Bacillus sp. SG-1]
gi|150388754 phosphoribosylglycinamide formyltransferase [Alkaliphilus metalliredigens QYMF]
gi|168335477 hypothetical protein Epulo_10467 [Epulopiscium sp. 'N.t. morphotype B']
gi|220930911 L-aspartate oxidase [Halothermothrix orenii H 168]
gi|16079728 hypothetical protein BSU26750 [Bacillus subtilis subsp. subtilis str. 168]
gi|111610131 glyceraldehyde 3-phosphate dehydrogenase [Lactobacillus helveticus CNRZ32]
gi|229229306 ATPase component of various ABC-type transport systems with duplicated ATPase domain [Desulfotomaculum a
gi|150391186 YheD [Alkaliphilus metalliredigens QYMF]
gi|229094029 Cell surface protein [Bacillus cereus Rock3-42]
gi|229541561 two component transcriptional regulator, LuxR family [Bacillus coagulans 36D1]
gi|192808885 YjgB [Geobacillus sp. Y412MC10]
gi|18310785 hypothetical protein CPE1803 [Clostridium perfringens str. 13]
gi|172057364 hypothetical protein Exig_1335 [Exiguobacterium sibiricum 255-15]

Probability Based Mowse Score

Ions score is -10*Log(P), where P is the probability that the observed match is a random event.
Individual ions scores > 52 indicate identity or extensive homology (p<0.05).
Protein scores are derived from ions scores as a non-probabilistic basis for ranking protein hits.

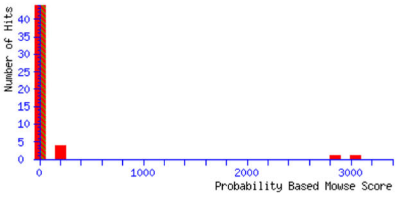

Peptide Summary Report

Format As | Peptide Summary | Help

Significance threshold p< 0.05 | Max. number of hits AUTO

Standard scoring ○ MudPIT scoring ◉ | Ions score or expect cut-off 0 | Show sub-sets 0

Show pop-ups ◉ Suppress pop-ups ○ | Sort unassigned Decreasing Score | Require bold red ☐

(2-3) Peptide Summary Report (protein spot 2, IE-2) Other Firmicutes

MATRIX SCIENCE Mascot Search Results

User : **liaopc**
Email :
Search title : **IE-2**
MS data file : **merge.txt**
Database : **NCBInr 090616 (9069431 sequences; 3106408609 residues)**
Taxonomy : **Other Firmicutes (967613 sequences)**
Timestamp : **10 Jan 2012 at 08:44:03 GMT**
Protein hits :
gi|42518797 glyceraldehyde 3-phosphate dehydrogenase [Lactobacillus johnsonii NCC 533]
gi|227520527 glyceraldehyde-3-phosphate dehydrogenase, phosphorylating [Lactobacillus gasseri JV-V03]
gi|227361679 glyceraldehyde-3-phosphate dehydrogenase [Lactobacillus sakei subsp. carnosus DSM 15831]
gi|227525966 glyceraldehyde-3-phosphate dehydrogenase (phosphorylating) [Lactobacillus jensenii JV-V16]
gi|90962141 glyceraldehyde 3-phosphate dehydrogenase [Lactobacillus salivarius UCC118]
gi|20799584 glyceraldehyde-3-phosphate dehydrogenase [Staphylococcus arlettae]
gi|227500542 DNA-binding protein [Anaerococcus tetradius ATCC 35098]
gi|148380680 hydrolase [Clostridium botulinum A str. ATCC 3502]
gi|197301564 hypothetical protein RUMLAC_00290 [Ruminococcus lactaris ATCC 29176]
gi|116492261 glyceraldehyde-3-phosphate dehydrogenase [Pediococcus pentosaceus ATCC 25745]
gi|229828817 hypothetical protein GCWU000342_00900 [Shuttleworthia satelles DSM 14600]
gi|146295492 hypothetical protein Csac_0434 [Caldicellulosiruptor saccharolyticus DSM 8903]
gi|170016655 glyceraldehyde-3-phosphate dehydrogenase/erythrose-4-phosphate dehydrogenase [Leuconostoc citreum KM20]
gi|229175622 Cell surface protein [Bacillus cereus MM3]
gi|150016208 trigger factor [Clostridium beijerinckii NCIMB 8052]
gi|20799602 glyceraldehyde-3-phosphate dehydrogenase [Staphylococcus piscifermentans]
gi|1706361 RecName: Full=Transcriptional regulatory protein degU
gi|148378084 hypothetical protein CBO0076 [Clostridium botulinum A str. ATCC 3502]
gi|154502799 hypothetical protein RUMGNA_00613 [Ruminococcus gnavus ATCC 29149]
gi|228995199 Collagen adhesion protein [Bacillus pseudomycoides DSM 12442]
gi|228583035 trigger factor protein [Clostridium sp. 7_2_43FAA]
gi|149183693 hypothetical protein BSG1_13561 [Bacillus sp. SG-1]
gi|150388754 phosphoribosylglycinamide formyltransferase [Alkaliphilus metalliredigens QYMF]
gi|168335477 hypothetical protein Epulo_10467 [Epulopiscium sp. 'N.t. morphotype B']
gi|220930911 L-aspartate oxidase [Halothermothrix orenii H 168]
gi|111610131 glyceraldehyde 3-phosphate dehydrogenase [Lactobacillus helveticus CNRZ32]
gi|229229306 ATPase component of various ABC-type transport systems with duplicated ATPase domain [Desulfotomaculum a
gi|150391186 YheD [Alkaliphilus metalliredigens QYMF]
gi|229094029 Cell surface protein [Bacillus cereus Rock3-42]
gi|229541561 two component transcriptional regulator, LuxR family [Bacillus coagulans 36D1]
gi|192808885 YjgB [Geobacillus sp. Y412MC10]
gi|18310785 hypothetical protein CPE1803 [Clostridium perfringens str. 13]
gi|172057364 hypothetical protein Exig_1335 [Exiguobacterium sibiricum 255-15]
gi|20799590 glyceraldehyde-3-phosphate dehydrogenase [Staphylococcus chromogenes]

Probability Based Mowse Score

Ions score is -10*Log(P), where P is the probability that the observed match is a random event.
Individual ions scores > 52 indicate identity or extensive homology (p<0.05).
Protein scores are derived from ions scores as a non-probabilistic basis for ranking protein hits.

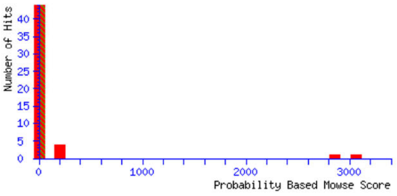

Peptide Summary Report

Format As | Peptide Summary | Help

Significance threshold p< 0.05 Max. number of hits AUTO

Standard scoring ○ MudPIT scoring ◉ Ions score or expect cut-off 0 Show sub-sets 0

Show pop-ups ◉ Suppress pop-ups ○ Sort unassigned Decreasing Score Require bold red ☐

(3-1) Peptide Summary Report (protein spot 3, IE-3) Bacteria (Eubacteria)

Mascot Search Results

User : liaopc
Email :
Search title : IE-3
MS data file : merge.txt
Database : NCBInr 090616 (9069431 sequences; 3106408609 residues)
Taxonomy : Bacteria (Eubacteria) (4853765 sequences)
Timestamp : 10 Jan 2012 at 04:04:37 GMT
Protein hits :

gi|42518797	glyceraldehyde 3-phosphate dehydrogenase [Lactobacillus johnsonii NCC 533]
gi|227520527	glyceraldehyde-3-phosphate dehydrogenase, phosphorylating [Lactobacillus gasseri JV-V03]
gi|227361679	glyceraldehyde-3-phosphate dehydrogenase [Lactobacillus sakei subsp. carnosus DSM 15831]
gi|227525966	glyceraldehyde-3-phosphate dehydrogenase (phosphorylating) [Lactobacillus jensenii JV-V16]
gi|111610131	glyceraldehyde 3-phosphate dehydrogenase [Lactobacillus helveticus CNRZ32]
gi|116494473	glyceraldehyde-3-phosphate dehydrogenase [Lactobacillus casei ATCC 334]
gi|90962141	glyceraldehyde 3-phosphate dehydrogenase [Lactobacillus salivarius UCC118]
gi|116492261	glyceraldehyde-3-phosphate dehydrogenase [Pediococcus pentosaceus ATCC 25745]
gi|170016655	glyceraldehyde-3-phosphate dehydrogenase/erythrose-4-phosphate dehydrogenase [Leuconostoc citreum KM20]
gi|227874212	zinc-exporting ATPase [Oribacterium sinus F0268]
gi|148252055	glyceraldehyde-3-phosphate dehydrogenase [Bradyrhizobium sp. BTAi1]
gi|225135183	Helicase conserved C-terminal domain protein [Ruegeria sp. R11]
gi|237751407	glycosyl transferase [Helicobacter bilis ATCC 43879]
gi|57234620	glyceraldehyde-3-phosphate dehydrogenase, type I [Dehalococcoides ethenogenes 195]
gi|20799584	glyceraldehyde-3-phosphate dehydrogenase [Staphylococcus arlettae]
gi|227979847	putative proteasome component/protein of unknown function, DUF275 [Tsukamurella paurometabola DSM 20162]
gi|229880970	CoA-substrate-specific enzyme activase, putative [Slackia heliotrinireducens DSM 20476]
gi|226312803	two-component response regulator [Brevibacillus brevis NBRC 100599]
gi|148266124	hypothetical protein Gura_4114 [Geobacter uraniireducens Rf4]
gi|126347884	putative glyceraldehyde 3-phosphate dehydrogenase [Streptomyces ambofaciens ATCC 23877]
gi|55821675	penicillin binding protein 2X [Streptococcus thermophilus LMG 18311]
gi|24213647	polynucleotide phosphorylase/polyadenylase [Leptospira interrogans serovar Lai str. 56601]
gi|39996379	transcription elongation factor GreA [Geobacter sulfurreducens PCA]
gi|167951074	hypothetical protein Epers_33717 [Endoriftia persephone 'Hot96_1+Hot96_2']
gi|228583035	trigger factor protein [Clostridium sp. 7_2_43FAA]
gi|149199701	hypothetical protein LNTAR_25135 [Lentisphaera araneosa HTCC2155]
gi|154502799	hypothetical protein RUMGNA_00613 [Ruminococcus gnavus ATCC 29149]
gi|227500542	DNA-binding protein [Anaerococcus tetradius ATCC 35098]
gi|94972048	lipoprotein signal peptidase [Deinococcus geothermalis DSM 11300]
gi|23099893	glyceraldehyde-3-phosphate dehydrogenase [Oceanobacillus iheyensis HTE831]
gi|42561108	holo-[acyl-carrier-protein] synthase [Mycoplasma mycoides subsp. mycoides SC str. PG1]
gi|224476119	putative pyridine nucleotide-disulfide oxidoreductase [Staphylococcus carnosus subsp. carnosus TM300]
gi|194246487	hypothetical protein ATP_00086 [Candidatus Phytoplasma mali]
gi|93007110	ATP-dependent helicase HepA [Psychrobacter cryohalolentis K5]
gi|226310996	glyceraldehyde 3-phosphate dehydrogenase [Brevibacillus brevis NBRC 100599]
gi|197301564	hypothetical protein RUMLAC_00290 [Ruminococcus lactaris ATCC 29176]
gi|91204595	similar to (3R)-hydroxymyristoyl acyl carrier protein dehydrase [Candidatus Kuenenia stuttgartiensis]
gi|404160	outer membrane protein (40K) [Fusobacterium nucleatum]
gi|84494635	putative glycerol phosphate dehydrogenase [Janibacter sp. HTCC2649]
gi|146295492	hypothetical protein Csac_0434 [Caldicellulosiruptor saccharolyticus DSM 8903]
gi|20799590	glyceraldehyde-3-phosphate dehydrogenase [Staphylococcus chromogenes]
gi|224391617	cobalamin biosynthesis protein CobT [Candidatus Pelagibacter sp. HTCC7211]
gi|172063180	NMT1/THI5-like domain-containing protein [Burkholderia ambifaria MC40-6]
gi|167764151	hypothetical protein BACSTE_02535 [Bacteroides stercoris ATCC 43183]
gi|162450188	hypothetical protein sce1916 [Sorangium cellulosum 'So ce 56']
gi|91202749	strongly similar to glyceraldehyde-3-phosphate dehydrogenase [Candidatus Kuenenia stuttgartiensis]

Probability Based Mowse Score

Ions score is -10*Log(P), where P is the probability that the observed match is a random event.
Individual ions scores > 59 indicate identity or extensive homology (p<0.05).
Protein scores are derived from ions scores as a non-probabilistic basis for ranking protein hits.

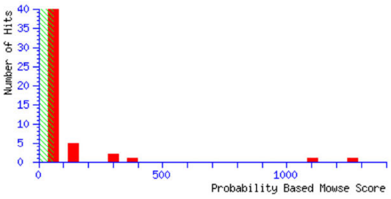

Peptide Summary Report

Format As | Peptide Summary | Help

Significance threshold p< 0.05 | Max. number of hits AUTO

Standard scoring ◉ MudPIT scoring ○ | Ions score or expect cut-off 0 | Show sub-sets 0

Show pop-ups ◉ Suppress pop-ups ○ | Sort unassigned Decreasing Score | Require bold red ☐

(3-2) Peptide Summary Report (protein spot 3, IE-3) Firmicutes (gram-positive bacteria)

Mascot Search Results

User : **liaopc**
Email :
Search title : **IE-3**
MS data file : **merge.txt**
Database : **NCBInr 090616 (9069431 sequences; 3106408609 residues)**
Taxonomy : **Firmicutes (gram-positive bacteria) (1006590 sequences)**
Timestamp : **10 Jan 2012 at 09:31:47 GMT**
Protein hits : gi|42518797 glyceraldehyde 3-phosphate dehydrogenase [Lactobacillus johnsonii NCC 533]
gi|227520527 glyceraldehyde-3-phosphate dehydrogenase, phosphorylating [Lactobacillus gasseri JV-V03]
gi|58337019 glyceraldehyde-3-p dehydrogenase [Lactobacillus acidophilus NCFM]
gi|227361679 glyceraldehyde-3-phosphate dehydrogenase [Lactobacillus sakei subsp. carnosus DSM 15831]
gi|227525966 glyceraldehyde-3-phosphate dehydrogenase (phosphorylating) [Lactobacillus jensenii JV-V16]
gi|116494473 glyceraldehyde-3-phosphate dehydrogenase [Lactobacillus casei ATCC 334]
gi|20799584 glyceraldehyde-3-phosphate dehydrogenase [Staphylococcus arlettae]
gi|90962141 glyceraldehyde 3-phosphate dehydrogenase [Lactobacillus salivarius UCC118]
gi|116492261 glyceraldehyde-3-phosphate dehydrogenase [Pediococcus pentosaceus ATCC 25745]
gi|111610131 glyceraldehyde 3-phosphate dehydrogenase [Lactobacillus helveticus CNRZ32]
gi|170016655 glyceraldehyde-3-phosphate dehydrogenase/erythrose-4-phosphate dehydrogenase [Leuconostoc citreum KM20]
gi|227500542 DNA-binding protein [Anaerococcus tetradius ATCC 35098]
gi|20799590 glyceraldehyde-3-phosphate dehydrogenase [Staphylococcus chromogenes]
gi|197301564 hypothetical protein RUMLAC_00290 [Ruminococcus lactaris ATCC 29176]
gi|148380680 hydrolase [Clostridium botulinum A str. ATCC 3502]
gi|148378084 hypothetical protein CBO0076 [Clostridium botulinum A str. ATCC 3502]
gi|228583035 trigger factor protein [Clostridium sp. 7_2_43FAA]
gi|154502799 hypothetical protein RUMGNA_00613 [Ruminococcus gnavus ATCC 29149]
gi|224476119 putative pyridine nucleotide-disulfide oxidoreductase [Staphylococcus carnosus subsp. carnosus TM300]
gi|146295492 hypothetical protein Csac_0434 [Caldicellulosiruptor saccharolyticus DSM 8903]
gi|150388754 phosphoribosylglycinamide formyltransferase [Alkaliphilus metalliredigens QYMF]
gi|150391186 YheD [Alkaliphilus metalliredigens QYMF]
gi|20799602 glyceraldehyde-3-phosphate dehydrogenase [Staphylococcus piscifermentans]
gi|1706361 RecName: Full=Transcriptional regulatory protein degU
gi|229175622 Cell surface protein [Bacillus cereus MM3]
gi|212640452 S-layer glycoprotein protein, contains three N-terminal SLH domains [Anoxybacillus flavithermus WK1]
gi|222529355 von Willebrand factor type A [Anaerocellum thermophilum DSM 6725]
gi|134298892 hypothetical protein Dred_1028 [Desulfotomaculum reducens MI-1]
gi|163763733 tetrahydrofolate dehydrogenase/cyclohydrolase [Bacillus selenitireducens MLS10]
gi|228982852 hypothetical protein bthur0002_59850 [Bacillus thuringiensis Bt407]

Probability Based Mowse Score

Ions score is -10*Log(P), where P is the probability that the observed match is a random event.
Individual ions scores > 52 indicate identity or extensive homology (p<0.05).
Protein scores are derived from ions scores as a non-probabilistic basis for ranking protein hits.

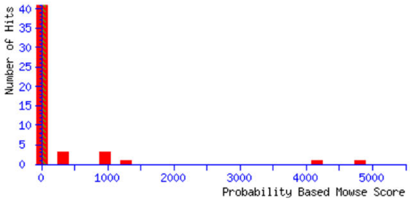

Peptide Summary Report

Format As | Peptide Summary | Help

Significance threshold p< 0.05 Max. number of hits AUTO

Standard scoring ○ MudPIT scoring ◉ Ions score or expect cut-off 0 Show sub-sets 0

Show pop-ups ◉ Suppress pop-ups ○ Sort unassigned Decreasing Score Require bold red ☐

(3-3) Peptide Summary Report (protein spot 3, IE-3) Other Firmicutes

Mascot Search Results

User : liaopc
Email :
Search title : IE-3
MS data file : merge.txt
Database : NCBInr 090616 (9069431 sequences; 3106408609 residues)
Taxonomy : Other Firmicutes (967613 sequences)
Timestamp : 11 Jan 2012 at 01:21:23 GMT
Protein hits : gi|42518797 glyceraldehyde 3-phosphate dehydrogenase [Lactobacillus johnsonii NCC 533]
gi|227520527 glyceraldehyde-3-phosphate dehydrogenase, phosphorylating [Lactobacillus gasseri JV-V03]
gi|58337019 glyceraldehyde-3-p dehydrogenase [Lactobacillus acidophilus NCFM]
gi|227361679 glyceraldehyde-3-phosphate dehydrogenase [Lactobacillus sakei subsp. carnosus DSM 15831]
gi|227525966 glyceraldehyde-3-phosphate dehydrogenase (phosphorylating) [Lactobacillus jensenii JV-V16]
gi|116494473 glyceraldehyde-3-phosphate dehydrogenase [Lactobacillus casei ATCC 334]
gi|20799584 glyceraldehyde-3-phosphate dehydrogenase [Staphylococcus arlettae]
gi|90962141 glyceraldehyde 3-phosphate dehydrogenase [Lactobacillus salivarius UCC118]
gi|116492261 glyceraldehyde-3-phosphate dehydrogenase [Pediococcus pentosaceus ATCC 25745]
gi|111610131 glyceraldehyde 3-phosphate dehydrogenase [Lactobacillus helveticus CNRZ32]
gi|170016655 glyceraldehyde-3-phosphate dehydrogenase/erythrose-4-phosphate dehydrogenase [Leuconostoc citreum KM20]
gi|227500542 DNA-binding protein [Anaerococcus tetradius ATCC 35098]
gi|20799590 glyceraldehyde-3-phosphate dehydrogenase [Staphylococcus chromogenes]
gi|197301564 hypothetical protein RUMLAC_00290 [Ruminococcus lactaris ATCC 29176]
gi|148380680 hydrolase [Clostridium botulinum A str. ATCC 3502]
gi|148378084 hypothetical protein CBO0076 [Clostridium botulinum A str. ATCC 3502]
gi|228583035 trigger factor protein [Clostridium sp. 7_2_43FAA]
gi|154502799 hypothetical protein RUMGNA_00613 [Ruminococcus gnavus ATCC 29149]
gi|224476119 putative pyridine nucleotide-disulfide oxidoreductase [Staphylococcus carnosus subsp. carnosus TM300]
gi|146295492 hypothetical protein Csac_0434 [Caldicellulosiruptor saccharolyticus DSM 8903]
gi|150388754 phosphoribosylglycinamide formyltransferase [Alkaliphilus metalliredigens QYMF]
gi|20799602 glyceraldehyde-3-phosphate dehydrogenase [Staphylococcus piscifermentans]
gi|150391186 YheD [Alkaliphilus metalliredigens QYMF]
gi|1706361 RecName: Full=Transcriptional regulatory protein degU
gi|229175622 Cell surface protein [Bacillus cereus MM3]
gi|212640452 S-layer glycoprotein protein, contains three N-terminal SLH domains [Anoxybacillus flavithermus WK1]
gi|222529355 von Willebrand factor type A [Anaerocellum thermophilum DSM 6725]
gi|134298892 hypothetical protein Dred_1028 [Desulfotomaculum reducens MI-1]
gi|163763733 tetrahydrofolate dehydrogenase/cyclohydrolase [Bacillus selenitireducens MLS10]
gi|228982852 hypothetical protein bthur0002_59850 [Bacillus thuringiensis Bt407]

Probability Based Mowse Score

Ions score is -10*Log(P), where P is the probability that the observed match is a random event.
Individual ions scores > 52 indicate identity or extensive homology (p<0.05).
Protein scores are derived from ions scores as a non-probabilistic basis for ranking protein hits.

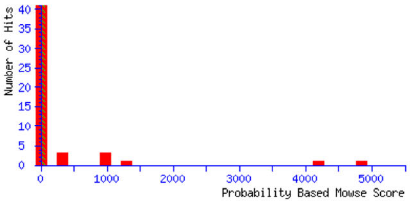

Peptide Summary Report

Format As | Peptide Summary | Help

Significance threshold p< 0.05 | Max. number of hits AUTO

Standard scoring ○ MudPIT scoring ◉ | Ions score or expect cut-off 0 | Show sub-sets 0

Show pop-ups ◉ Suppress pop-ups ○ | Sort unassigned Decreasing Score | Require bold red ☐

(4-1) Peptide Summary Report (protein spot 4, IE-4) Bacteria (Eubacteria)

MATRIX SCIENCE Mascot Search Results

```
User            : liaopc
Email           :
Search title    : IE-4
MS data file    : merge.txt
Database        : NCBInr 090616 (9069431 sequences; 3106408609 residues)
Taxonomy        : Bacteria (Eubacteria) (4853765 sequences)
Timestamp       : 10 Jan 2012 at 05:46:12 GMT
Protein hits    : gi|42518797  glyceraldehyde 3-phosphate dehydrogenase [Lactobacillus johnsonii NCC 533]
                  gi|227520527 glyceraldehyde-3-phosphate dehydrogenase, phosphorylating [Lactobacillus gasseri JV-V03]
                  gi|227361679 glyceraldehyde-3-phosphate dehydrogenase [Lactobacillus sakei subsp. carnosus DSM 15831]
                  gi|227525966 glyceraldehyde-3-phosphate dehydrogenase (phosphorylating) [Lactobacillus jensenii JV-V16]
                  gi|116492261 glyceraldehyde-3-phosphate dehydrogenase [Pediococcus pentosaceus ATCC 25745]
                  gi|111610131 glyceraldehyde 3-phosphate dehydrogenase [Lactobacillus helveticus CNRZ32]
                  gi|90962141  glyceraldehyde 3-phosphate dehydrogenase [Lactobacillus salivarius UCC118]
                  gi|170016655 glyceraldehyde-3-phosphate dehydrogenase/erythrose-4-phosphate dehydrogenase [Leuconostoc citreum KM20]
                  gi|225103805 glyceraldehyde-3-phosphate dehydrogenase, type I [Methylophaga sp. DMS010]
                  gi|94499337  hypothetical protein RED65_11124 [Oceanobacter sp. RED65]
                  gi|148252055 glyceraldehyde-3-phosphate dehydrogenase [Bradyrhizobium sp. BTAi1]
                  gi|196230628 hypothetical protein CfE428DRAFT_2655 [Chthoniobacter flavus Ellin428]
                  gi|57234620  glyceraldehyde-3-phosphate dehydrogenase, type I [Dehalococcoides ethenogenes 195]
                  gi|87161642  alkyl hydroperoxide reductase subunit F [Staphylococcus aureus subsp. aureus USA300_FPR3757]
                  gi|20799584  glyceraldehyde-3-phosphate dehydrogenase [Staphylococcus arlettae]
                  gi|126347884 putative glyceraldehyde 3-phosphate dehydrogenase [Streptomyces ambofaciens ATCC 23877]
                  gi|84494635  putative glycerol phosphate dehydrogenase [Janibacter sp. HTCC2649]
                  gi|218149927 phosphopantothenoylcysteine decarboxylase/phosphopantothenate/cysteine ligase [Desulfovibrio salexigens
                  gi|196036424 phage infection protein [Bacillus cereus W]
                  gi|227979847 putative proteasome component/protein of unknown function, DUF275 [Tsukamurella paurometabola DSM 20162]
                  gi|24213647  polynucleotide phosphorylase/polyadenylase [Leptospira interrogans serovar Lai str. 56601]
                  gi|224391617 cobalamin biosynthesis protein CobT [Candidatus Pelagibacter sp. HTCC7211]
                  gi|23099893  glyceraldehyde-3-phosphate dehydrogenase [Oceanobacillus iheyensis HTE831]
                  gi|197301564 hypothetical protein RUMLAC_00290 [Ruminococcus lactaris ATCC 29176]
                  gi|227500542 DNA-binding protein [Anaerococcus tetradius ATCC 35098]
                  gi|238027761 hypothetical protein bglu_1g21850 [Burkholderia glumae BGR1]
                  gi|238927776 conserved hypothetical protein [Selenomonas flueggei ATCC 43531]
                  gi|149199701 hypothetical protein LNTAR_25135 [Lentisphaera araneosa HTCC2155]
                  gi|226310996 glyceraldehyde 3-phosphate dehydrogenase [Brevibacillus brevis NBRC 100599]
                  gi|1706361   RecName: Full=Transcriptional regulatory protein degU
                  gi|225022855 hypothetical protein CORMATOL_02901 [Corynebacterium matruchotii ATCC 33806]
                  gi|218439438 DNA topoisomerase I [Cyanothece sp. PCC 7424]
                  gi|20799590  glyceraldehyde-3-phosphate dehydrogenase [Staphylococcus chromogenes]
                  gi|114321786 TolC family type I secretion outer membrane protein [Alkalilimnicola ehrlichii MLHE-1]
                  gi|110835656 aminoglycoside-3-phospho transferase [Acetobacter pasteurianus]
                  gi|229175622 Cell surface protein [Bacillus cereus MM3]
                  gi|999601    Chain O, The Crystal Structure Of Holo-Glyceraldehyde-3-Phosphate Dehydrogenase From The Hyperthermophil
                  gi|162450188 hypothetical protein sce1916 [Sorangium cellulosum 'So ce 56']
                  gi|237751407 glycosyl transferase [Helicobacter bilis ATCC 43879]
                  gi|167764151 hypothetical protein BACSTE_02535 [Bacteroides stercoris ATCC 43183]
```

Probability Based Mowse Score

Ions score is -10*Log(P), where P is the probability that the observed match is a random event.
Individual ions scores > 59 indicate identity or extensive homology (p<0.05).
Protein scores are derived from ions scores as a non-probabilistic basis for ranking protein hits.

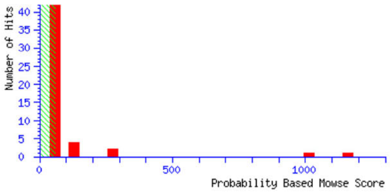

Peptide Summary Report

Format As | Peptide Summary | Help

Significance threshold p< 0.05 Max. number of hits AUTO

Standard scoring ◉ MudPIT scoring ○ Ions score or expect cut-off 0 Show sub-sets 0

Show pop-ups ◉ Suppress pop-ups ○ Sort unassigned Decreasing Score Require bold red ☐

(4-2) Peptide Summary Report (protein spot 4, IE-4) Firmicutes (gram-positive bacteria)

MATRIX SCIENCE Mascot Search Results

User : liaopc
Email :
Search title : IE-4
MS data file : merge.txt
Database : NCBInr 090616 (9069431 sequences; 3106408609 residues)
Taxonomy : Firmicutes (gram-positive bacteria) (1006590 sequences)
Timestamp : 10 Jan 2012 at 09:46:08 GMT
Protein hits :

gi|42518797 glyceraldehyde 3-phosphate dehydrogenase [Lactobacillus johnsonii NCC 533]
gi|227520527 glyceraldehyde-3-phosphate dehydrogenase, phosphorylating [Lactobacillus gasseri JV-V03]
gi|227361679 glyceraldehyde-3-phosphate dehydrogenase [Lactobacillus sakei subsp. carnosus DSM 15831]
gi|227525966 glyceraldehyde-3-phosphate dehydrogenase (phosphorylating) [Lactobacillus jensenii JV-V16]
gi|20799584 glyceraldehyde-3-phosphate dehydrogenase [Staphylococcus arlettae]
gi|90962141 glyceraldehyde 3-phosphate dehydrogenase [Lactobacillus salivarius UCC118]
gi|116492261 glyceraldehyde-3-phosphate dehydrogenase [Pediococcus pentosaceus ATCC 25745]
gi|170016655 glyceraldehyde-3-phosphate dehydrogenase/erythrose-4-phosphate dehydrogenase [Leuconostoc citreum KM20]
gi|20799590 glyceraldehyde-3-phosphate dehydrogenase [Staphylococcus chromogenes]
gi|227500542 DNA-binding protein [Anaerococcus tetradius ATCC 35098]
gi|197301564 hypothetical protein RUMLAC_00290 [Ruminococcus lactaris ATCC 29176]
gi|148380680 hydrolase [Clostridium botulinum A str. ATCC 3502]
gi|148378084 hypothetical protein CBO0076 [Clostridium botulinum A str. ATCC 3502]
gi|229175622 Cell surface protein [Bacillus cereus MM3]
gi|49478820 cell surface anchor [Bacillus thuringiensis serovar konkukian str. 97-27]
gi|228981615 Cell surface protein [Bacillus thuringiensis Bt407]
gi|20799602 glyceraldehyde-3-phosphate dehydrogenase [Staphylococcus piscifermentans]
gi|238927776 conserved hypothetical protein [Selenomonas flueggei ATCC 43531]
gi|1706361 RecName: Full=Transcriptional regulatory protein degU
gi|111610131 glyceraldehyde 3-phosphate dehydrogenase [Lactobacillus helveticus CNRZ32]
gi|220931194 hypothetical protein Hore_03460 [Halothermothrix orenii H 168]
gi|229229306 ATPase component of various ABC-type transport systems with duplicated ATPase domain [Desulfotomaculum a
gi|169187565 two component transcriptional regulator, LuxR family [Paenibacillus sp. JDR-2]
gi|146295492 hypothetical protein Csac_0434 [Caldicellulosiruptor saccharolyticus DSM 8903]
gi|229829382 hypothetical protein GCWU000342_01471 [Shuttleworthia satelles DSM 14600]
gi|225027108 hypothetical protein EUBHAL_01364 [Eubacterium hallii DSM 3353]
gi|154502799 hypothetical protein RUMGNA_00613 [Ruminococcus gnavus ATCC 29149]
gi|212640452 S-layer glycoprotein protein, contains three N-terminal SLH domains [Anoxybacillus flavithermus WK1]
gi|42519693 hypothetical protein LJ0632 [Lactobacillus johnsonii NCC 533]
gi|228918843 hypothetical protein bthur0012_59520 [Bacillus thuringiensis serovar pulsiensis BGSC 4CC1]
gi|222529096 glycoside hydrolase family 4 [Anaerocellum thermophilum DSM 6725]
gi|229541561 two component transcriptional regulator, LuxR family [Bacillus coagulans 36D1]

Probability Based Mowse Score

Ions score is -10*Log(P), where P is the probability that the observed match is a random event.
Individual ions scores > 52 indicate identity or extensive homology (p<0.05).
Protein scores are derived from ions scores as a non-probabilistic basis for ranking protein hits.

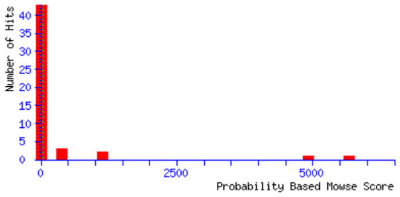

Peptide Summary Report

Format As [Peptide Summary] Help

Significance threshold p< 0.05 Max. number of hits AUTO

Standard scoring ○ MudPIT scoring ◉ Ions score or expect cut-off 0 Show sub-sets 0

Show pop-ups ◉ Suppress pop-ups ○ Sort unassigned [Decreasing Score] Require bold red ☐

(4-3) Peptide Summary Report (protein spot 4, IE-4) Other Firmicutes

Mascot Search Results

```
User          : liaopc
Email         :
Search title  : IE-4
MS data file  : merge.txt
Database      : NCBInr 090616 (9069431 sequences; 3106408609 residues)
Taxonomy      : Other Firmicutes (967613 sequences)
Timestamp     : 11 Jan 2012 at 01:54:33 GMT
Protein hits  : gi|42518797  glyceraldehyde 3-phosphate dehydrogenase [Lactobacillus johnsonii NCC 533]
                gi|227520527 glyceraldehyde-3-phosphate dehydrogenase, phosphorylating [Lactobacillus gasseri JV-V03]
                gi|227361679 glyceraldehyde-3-phosphate dehydrogenase [Lactobacillus sakei subsp. carnosus DSM 15831]
                gi|227525966 glyceraldehyde-3-phosphate dehydrogenase (phosphorylating) [Lactobacillus jensenii JV-V16]
                gi|20799584  glyceraldehyde-3-phosphate dehydrogenase [Staphylococcus arlettae]
                gi|90962141  glyceraldehyde 3-phosphate dehydrogenase [Lactobacillus salivarius UCC118]
                gi|116492261 glyceraldehyde-3-phosphate dehydrogenase [Pediococcus pentosaceus ATCC 25745]
                gi|20799590  glyceraldehyde-3-phosphate dehydrogenase [Staphylococcus chromogenes]
                gi|170016655 glyceraldehyde-3-phosphate dehydrogenase/erythrose-4-phosphate dehydrogenase [Leuconostoc citreum KM20]
                gi|148380680 hydrolase [Clostridium botulinum A str. ATCC 3502]
                gi|227500542 DNA-binding protein [Anaerococcus tetradius ATCC 35098]
                gi|197301564 hypothetical protein RUMLAC_00290 [Ruminococcus lactaris ATCC 29176]
                gi|148378084 hypothetical protein CBO0076 [Clostridium botulinum A str. ATCC 3502]
                gi|229175622 Cell surface protein [Bacillus cereus MM3]
                gi|49478820  cell surface anchor [Bacillus thuringiensis serovar konkukian str. 97-27]
                gi|228981615 Cell surface protein [Bacillus thuringiensis Bt407]
                gi|20799602  glyceraldehyde-3-phosphate dehydrogenase [Staphylococcus piscifermentans]
                gi|238927776 conserved hypothetical protein [Selenomonas flueggei ATCC 43531]
                gi|1706361   RecName: Full=Transcriptional regulatory protein degU
                gi|111610131 glyceraldehyde 3-phosphate dehydrogenase [Lactobacillus helveticus CNRZ32]
                gi|220931194 hypothetical protein Hore_03460 [Halothermothrix orenii H 168]
                gi|229229306 ATPase component of various ABC-type transport systems with duplicated ATPase domain [Desulfotomaculum a
                gi|169187565 two component transcriptional regulator, LuxR family [Paenibacillus sp. JDR-2]
                gi|146295492 hypothetical protein Csac_0434 [Caldicellulosiruptor saccharolyticus DSM 8903]
                gi|229829382 hypothetical protein GCWU000342_01471 [Shuttleworthia satelles DSM 14600]
                gi|225027108 hypothetical protein EUBHAL_01364 [Eubacterium hallii DSM 3353]
                gi|154502799 hypothetical protein RUMGNA_00613 [Ruminococcus gnavus ATCC 29149]
                gi|212640452 S-layer glycoprotein protein, contains three N-terminal SLH domains [Anoxybacillus flavithermus WK1]
                gi|42519693  hypothetical protein LJ0632 [Lactobacillus johnsonii NCC 533]
                gi|228918843 hypothetical protein bthur0012_59520 [Bacillus thuringiensis serovar pulsiensis BGSC 4CC1]
                gi|222529096 glycoside hydrolase family 4 [Anaerocellum thermophilum DSM 6725]
                gi|229541561 two component transcriptional regulator, LuxR family [Bacillus coagulans 36D1]
```

Probability Based Mowse Score

Ions score is -10*Log(P), where P is the probability that the observed match is a random event.
Individual ions scores > 52 indicate identity or extensive homology (p<0.05).
Protein scores are derived from ions scores as a non-probabilistic basis for ranking protein hits.

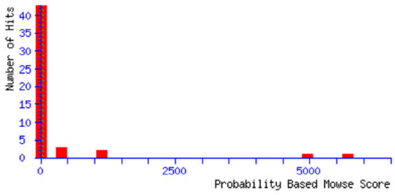

Peptide Summary Report

Format As | Peptide Summary | Help

Significance threshold p< 0.05 | Max. number of hits AUTO

Standard scoring ○ MudPIT scoring ◉ | Ions score or expect cut-off 0 | Show sub-sets 0

Show pop-ups ◉ Suppress pop-ups ○ | Sort unassigned Decreasing Score | Require bold red ☐

(5-1) Peptide Summary Report (protein spot 5, IE-5) Bacteria (Eubacteria)

{MATRIX SCIENCE} Mascot Search Results

**User**	: **liaopc**
**Email**	:
**Search title**	: **IE-5**
**MS data file**	: **merge.txt**
**Database**	: **NCBInr 090616 (9069431 sequences; 3106408609 residues)**
**Taxonomy**	: **Bacteria (Eubacteria) (4853765 sequences)**
**Timestamp**	: **10 Jan 2012 at 07:20:47 GMT**
**Protein hits**	: gi|42518797 glyceraldehyde 3-phosphate dehydrogenase [Lactobacillus johnsonii NCC 533]
	gi|227520527 glyceraldehyde-3-phosphate dehydrogenase, phosphorylating [Lactobacillus gasseri JV-V03]
	gi|227361679 glyceraldehyde-3-phosphate dehydrogenase [Lactobacillus sakei subsp. carnosus DSM 15831]
	gi|227525966 glyceraldehyde-3-phosphate dehydrogenase (phosphorylating) [Lactobacillus jensenii JV-V16]
	gi|111610131 glyceraldehyde 3-phosphate dehydrogenase [Lactobacillus helveticus CNRZ32]
	gi|90962141 glyceraldehyde 3-phosphate dehydrogenase [Lactobacillus salivarius UCC118]
	gi|158334876 hypothetical protein AM1_1713 [Acaryochloris marina MBIC11017]
	gi|237751407 glycosyl transferase [Helicobacter bilis ATCC 43879]
	gi|162148108 putative peptidyl-prolyl cis-trans isomerase B [Gluconacetobacter diazotrophicus PAl 5]
	gi|20799584 glyceraldehyde-3-phosphate dehydrogenase [Staphylococcus arlettae]
	gi|217077222 chromosome segregation SMC protein, putative [Thermosipho africanus TCF52B]
	gi|116492261 glyceraldehyde-3-phosphate dehydrogenase [Pediococcus pentosaceus ATCC 25745]
	gi|172057364 hypothetical protein Exig_1335 [Exiguobacterium sibiricum 255-15]
	gi|196228419 hypothetical protein CfE428DRAFT_0450 [Chthoniobacter flavus Ellin428]
	gi|160894823 hypothetical protein CLOL250_02373 [Clostridium sp. L2-50]
	gi|84494635 putative glycerol phosphate dehydrogenase [Janibacter sp. HTCC2649]
	gi|124024825 hypothetical protein NATL1_01121 [Prochlorococcus marinus str. NATL1A]
	gi|116491203 ABC-type metal ion transport system, periplasmic component/surface adhesin [Oenococcus oeni PSU-1]

Probability Based Mowse Score

Ions score is -10*Log(P), where P is the probability that the observed match is a random event.
Individual ions scores > 59 indicate identity or extensive homology (p<0.05).
Protein scores are derived from ions scores as a non-probabilistic basis for ranking protein hits.

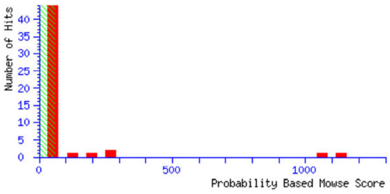

Peptide Summary Report

Format As | Peptide Summary | Help

Significance threshold p< 0.05 | Max. number of hits AUTO

Standard scoring ◉ MudPIT scoring ○ | Ions score or expect cut-off 0 | Show sub-sets 0

Show pop-ups ◉ Suppress pop-ups ○ | Sort unassigned Decreasing Score | Require bold red ☐

(5-2) Peptide Summary Report (protein spot 5, IE-5) Firmicutes (gram-positive bacteria)

Mascot Search Results

**User**	: liaopc
**Email**	:
**Search title**	: IE-5
**MS data file**	: merge.txt
**Database**	: NCBInr 090616 (9069431 sequences; 3106408609 residues)
**Taxonomy**	: Firmicutes (gram-positive bacteria) (1006590 sequences)
**Timestamp**	: 11 Jan 2012 at 02:17:38 GMT
**Protein hits**	: gi|42518797 glyceraldehyde 3-phosphate dehydrogenase [Lactobacillus johnsonii NCC 533]
	gi|227520527 glyceraldehyde-3-phosphate dehydrogenase, phosphorylating [Lactobacillus gasseri JV-V03]
	gi|227361679 glyceraldehyde-3-phosphate dehydrogenase [Lactobacillus sakei subsp. carnosus DSM 15831]
	gi|227525966 glyceraldehyde-3-phosphate dehydrogenase (phosphorylating) [Lactobacillus jensenii JV-V16]
	gi|116494473 glyceraldehyde-3-phosphate dehydrogenase [Lactobacillus casei ATCC 334]
	gi|111610131 glyceraldehyde 3-phosphate dehydrogenase [Lactobacillus helveticus CNRZ32]
	gi|20799584 glyceraldehyde-3-phosphate dehydrogenase [Staphylococcus arlettae]
	gi|116492261 glyceraldehyde-3-phosphate dehydrogenase [Pediococcus pentosaceus ATCC 25745]
	gi|172057364 hypothetical protein Exig_1335 [Exiguobacterium sibiricum 255-15]
	gi|160894823 hypothetical protein CLOL250_02373 [Clostridium sp. L2-50]
	gi|90962141 glyceraldehyde 3-phosphate dehydrogenase [Lactobacillus salivarius UCC118]
	gi|150016208 trigger factor [Clostridium beijerinckii NCIMB 8052]
	gi|150391186 YheD [Alkaliphilus metalliredigens QYMF]
	gi|154502799 hypothetical protein RUMGNA_00613 [Ruminococcus gnavus ATCC 29149]
	gi|148380680 hydrolase [Clostridium botulinum A str. ATCC 3502]
	gi|20799590 glyceraldehyde-3-phosphate dehydrogenase [Staphylococcus chromogenes]
	gi|170016655 glyceraldehyde-3-phosphate dehydrogenase/erythrose-4-phosphate dehydrogenase [Leuconostoc citreum KM20]
	gi|227500542 DNA-binding protein [Anaerococcus tetradius ATCC 35098]
	gi|192808885 YjgB [Geobacillus sp. Y412MC10]
	gi|225390610 hypothetical protein CLOSTASPAR_04365 [Clostridium asparagiforme DSM 15981]
	gi|150388754 phosphoribosylglycinamide formyltransferase [Alkaliphilus metalliredigens QYMF]

Probability Based Mowse Score

Ions score is -10*Log(P), where P is the probability that the observed match is a random event.
Individual ions scores > 52 indicate identity or extensive homology (p<0.05).
Protein scores are derived from ions scores as a non-probabilistic basis for ranking protein hits.

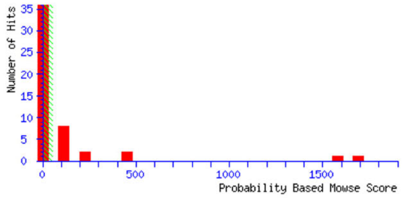

Peptide Summary Report

Format As [Peptide Summary] Help

Significance threshold p< [0.05] Max. number of hits [AUTO]

Standard scoring ○ MudPIT scoring ◉ Ions score or expect cut-off [0] Show sub-sets [0]

Show pop-ups ◉ Suppress pop-ups ○ Sort unassigned [Decreasing Score] Require bold red ☐

(5-3) Peptide Summary Report (protein spot 5, IE-5) Other Firmicutes

Mascot Search Results

```
User          : liaopc
Email         :
Search title  : IE-5
MS data file  : merge.txt
Database      : NCBInr 090616 (9069431 sequences; 3106408609 residues)
Taxonomy      : Other Firmicutes (967613 sequences)
Timestamp     : 11 Jan 2012 at 02:05:35 GMT
Protein hits  : gi|42518797  glyceraldehyde 3-phosphate dehydrogenase [Lactobacillus johnsonii NCC 533]
                gi|227520527 glyceraldehyde-3-phosphate dehydrogenase, phosphorylating [Lactobacillus gasseri JV-V03]
                gi|227361679 glyceraldehyde-3-phosphate dehydrogenase [Lactobacillus sakei subsp. carnosus DSM 15831]
                gi|227525966 glyceraldehyde-3-phosphate dehydrogenase (phosphorylating) [Lactobacillus jensenii JV-V16]
                gi|116494473 glyceraldehyde-3-phosphate dehydrogenase [Lactobacillus casei ATCC 334]
                gi|111610131 glyceraldehyde 3-phosphate dehydrogenase [Lactobacillus helveticus CNRZ32]
                gi|20799584  glyceraldehyde-3-phosphate dehydrogenase [Staphylococcus arlettae]
                gi|116492261 glyceraldehyde-3-phosphate dehydrogenase [Pediococcus pentosaceus ATCC 25745]
                gi|172057364 hypothetical protein Exig_1335 [Exiguobacterium sibiricum 255-15]
                gi|160894823 hypothetical protein CLOL250_02373 [Clostridium sp. L2-50]
                gi|90962141  glyceraldehyde 3-phosphate dehydrogenase [Lactobacillus salivarius UCC118]
                gi|150016208 trigger factor [Clostridium beijerinckii NCIMB 8052]
                gi|150391186 YheD [Alkaliphilus metalliredigens QYMF]
                gi|154502799 hypothetical protein RUMGNA_00613 [Ruminococcus gnavus ATCC 29149]
                gi|148380680 hydrolase [Clostridium botulinum A str. ATCC 3502]
                gi|20799590  glyceraldehyde-3-phosphate dehydrogenase [Staphylococcus chromogenes]
                gi|170016655 glyceraldehyde-3-phosphate dehydrogenase/erythrose-4-phosphate dehydrogenase [Leuconostoc citreum KM20]
                gi|227500542 DNA-binding protein [Anaerococcus tetradius ATCC 35098]
                gi|192808885 YjgB [Geobacillus sp. Y412MC10]
                gi|225390610 hypothetical protein CLOSTASPAR_04365 [Clostridium asparagiforme DSM 15981]
                gi|150388754 phosphoribosylglycinamide formyltransferase [Alkaliphilus metalliredigens QYMF]
                gi|220932533 DegT/DnrJ/EryC1/StrS aminotransferase [Halothermothrix orenii H 168]
```

Probability Based Mowse Score

Ions score is -10*Log(P), where P is the probability that the observed match is a random event.
Individual ions scores > 52 indicate identity or extensive homology (p<0.05).
Protein scores are derived from ions scores as a non-probabilistic basis for ranking protein hits.

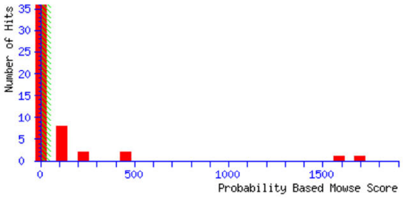

Peptide Summary Report

Format As [Peptide Summary] Help

Significance threshold p< [0.05] Max. number of hits [AUTO]

Standard scoring ○ MudPIT scoring ◉ Ions score or expect cut-off [0] Show sub-sets [0]

Show pop-ups ◉ Suppress pop-ups ○ Sort unassigned [Decreasing Score] Require bold red ☐

(6-1) Peptide Summary Report (protein spot 6, IE-6) Bacteria (Eubacteria)

Mascot Search Results

User : liaopc
Email :
Search title : IE-6
MS data file : merge.txt
Database : NCBInr 090616 (9069431 sequences; 3106408609 residues)
Taxonomy : Bacteria (Eubacteria) (4853765 sequences)
Timestamp : 10 Jan 2012 at 02:16:55 GMT
Protein hits :
gi|116629131 fructose/tagatose bisphosphate aldolase [Lactobacillus gasseri ATCC 33323]
gi|42518609 fructose-bisphosphate aldolase [Lactobacillus johnsonii NCC 533]
gi|227360402 fructose-bisphosphate aldolase [Lactobacillus sakei subsp. carnosus DSM 15831]
gi|227876934 fructose-bisphosphate aldolase [Lactobacillus crispatus JV-V01]
gi|164687868 hypothetical protein CLOBAR_01512 [Clostridium bartlettii DSM 16795]
gi|119961101 hypothetical protein AAur_3947 [Arthrobacter aurescens TC1]
gi|228948651 Cell surface protein [Bacillus thuringiensis serovar monterrey BGSC 4AJ1]
gi|229541583 galactokinase [Bacillus coagulans 36D1]
gi|114561971 UspA domain-containing protein [Shewanella frigidimarina NCIMB 400]
gi|229135967 transposase, IS605 [Bacillus cereus BDRD-ST196]
gi|196157130 DNA polymerase III subunit delta [Alteromonas macleodii 'Deep ecotype']
gi|237712148 alpha-1,2-fucosyltransferase [Bacteroides sp. 9_1_42FAA]
gi|224437247 lipid A biosynthesis lauroyl acyltransferase [Helicobacter cinaedi CCUG 18818]
gi|19704335 biotin synthase [Fusobacterium nucleatum subsp. nucleatum ATCC 25586]
gi|67922674 hypothetical protein CwatDRAFT_3622 [Crocosphaera watsonii WH 8501]
gi|1706361 RecName: Full=Transcriptional regulatory protein degU
gi|158321234 adenosylcobinamide-phosphate guanylyltransferase [Alkaliphilus oremlandii OhILAs]
gi|90407501 stringent starvation protein A [Psychromonas sp. CNPT3]
gi|73667069 hypothetical protein Ecaj_0447 [Ehrlichia canis str. Jake]
gi|237751407 glycosyl transferase [Helicobacter bilis ATCC 43879]
gi|197301564 hypothetical protein RUMLAC_00290 [Ruminococcus lactaris ATCC 29176]
gi|110638554 Zn-binding alcohol dehydrogenase [Cytophaga hutchinsonii ATCC 33406]
gi|123442441 hemin storage receptor protein [Yersinia enterocolitica subsp. enterocolitica 8081]
gi|149199701 hypothetical protein LNTAR_25135 [Lentisphaera araneosa HTCC2155]
gi|224391617 cobalamin biosynthesis protein CobT [Candidatus Pelagibacter sp. HTCC7211]
gi|163786638 hypothetical protein FBALC1_15672 [Flavobacteriales bacterium ALC-1]
gi|29840049 polymorphic outer membrane protein G family protein/autotransporter, putative [Chlamydophila caviae GPIC
gi|30262473 hypothetical protein BA2474 [Bacillus anthracis str. Ames]
gi|167764151 hypothetical protein BACSTE_02535 [Bacteroides stercoris ATCC 43183]
gi|42561108 holo-[acyl-carrier-protein] synthase [Mycoplasma mycoides subsp. mycoides SC str. PG1]
gi|15594477 fused transcript cleavage factor/uncharacterized domain-containing protein [Borrelia burgdorferi B31]
gi|153813549 hypothetical protein RUMOBE_03971 [Ruminococcus obeum ATCC 29174]

Probability Based Mowse Score

Ions score is -10*Log(P), where P is the probability that the observed match is a random event.
Individual ions scores > 59 indicate identity or extensive homology (p<0.05).
Protein scores are derived from ions scores as a non-probabilistic basis for ranking protein hits.

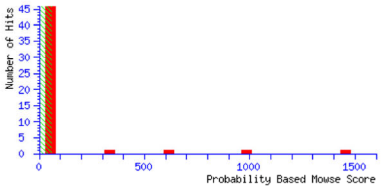

Peptide Summary Report

Format As | Peptide Summary | Help

Significance threshold p< 0.05 | Max. number of hits AUTO

Standard scoring ◉ MudPIT scoring ○ | Ions score or expect cut-off 0 | Show sub-sets 0

Show pop-ups ◉ Suppress pop-ups ○ | Sort unassigned Decreasing Score | Require bold red ☐

(6-2) Peptide Summary Report (protein spot 6, IE-6) Firmicutes (gram-positive bacteria)

Mascot Search Results

User : liaopc
Email :
Search title : IE-6
MS data file : merge.txt
Database : NCBInr 090616 (9069431 sequences; 3106408609 residues)
Taxonomy : Firmicutes (gram-positive bacteria) (1006590 sequences)
Timestamp : 11 Jan 2012 at 02:57:03 GMT
Protein hits :

gi|116629131	fructose/tagatose bisphosphate aldolase [Lactobacillus gasseri ATCC 33323]
gi|42518609	fructose-bisphosphate aldolase [Lactobacillus johnsonii NCC 533]
gi|227360402	fructose-bisphosphate aldolase [Lactobacillus sakei subsp. carnosus DSM 15831]
gi|227876934	fructose-bisphosphate aldolase [Lactobacillus crispatus JV-V01]
gi|164687868	hypothetical protein CLOBAR_01512 [Clostridium bartlettii DSM 16795]
gi|229135967	transposase, IS605 [Bacillus cereus BDRD-ST196]
gi|197301564	hypothetical protein RUMLAC_00290 [Ruminococcus lactaris ATCC 29176]
gi|30262473	hypothetical protein BA2474 [Bacillus anthracis str. Ames]
gi|158321234	adenosylcobinamide-phosphate guanylyltransferase [Alkaliphilus oremlandii OhILAs]
gi|169191287	UDP-N-acetylglucosamine 1-carboxyvinyltransferase [Paenibacillus sp. JDR-2]
gi|47567502	ABC transporter, ATP-binding protein [Bacillus cereus G9241]
gi|153813549	hypothetical protein RUMOBE_03971 [Ruminococcus obeum ATCC 29174]
gi|81429140	fructose-bisphosphate aldolase [Lactobacillus sakei subsp. sakei 23K]
gi|1706361	RecName: Full=Transcriptional regulatory protein degU
gi|168333288	hypothetical protein Epulo_00390 [Epulopiscium sp. 'N.t. morphotype B']
gi|228948651	Cell surface protein [Bacillus thuringiensis serovar monterrey BGSC 4AJ1]
gi|172058143	glutamate-1-semialdehyde-2,1-aminomutase [Exiguobacterium sibiricum 255-15]
gi|145952657	hypothetical protein CdifQ_04004036 [Clostridium difficile QCD-32g58]
gi|229229306	ATPase component of various ABC-type transport systems with duplicated ATPase domain [Desulfotomaculum a
gi|229092047	beta-lactamase class C [Bacillus cereus Rock3-42]
gi|228583035	trigger factor protein [Clostridium sp. 7_2_43FAA]
gi|229106627	hypothetical protein bcere0019_54130 [Bacillus cereus Rock3-28]
gi|187935417	serine protein kinase [Clostridium botulinum B str. Eklund 17B]
gi|229172724	Fis-type helix-turn-helix domain protein [Bacillus cereus MM3]
gi|152974405	sortase family protein [Bacillus cereus subsp. cytotoxis NVH 391-98]
gi|194398220	ABC transporter, ATP-binding protein [Streptococcus pneumoniae G54]
gi|238916527	basic amino acid/polyamine antiporter, APA family [Eubacterium eligens ATCC 27750]
gi|167766734	hypothetical protein CLOSS21_01240 [Clostridium sp. SS2/1]
gi|150391186	YheD [Alkaliphilus metalliredigens QYMF]
gi|222529355	von Willebrand factor type A [Anaerocellum thermophilum DSM 6725]
gi|192808568	hypothetical protein GYMC10DRAFT_1077 [Geobacillus sp. Y412MC10]
gi|212640452	S-layer glycoprotein protein, contains three N-terminal SLH domains [Anoxybacillus flavithermus WK1]
gi|52078774	putative lipoprotein [Bacillus licheniformis ATCC 14580]
gi|229541561	two component transcriptional regulator, LuxR family [Bacillus coagulans 36D1]
gi|194016979	YpiF [Bacillus pumilus ATCC 7061]
gi|160939974	hypothetical protein CLOBOL_04864 [Clostridium bolteae ATCC BAA-613]
gi|229051384	hypothetical protein bcere0027_53120 [Bacillus cereus AH676]
gi|218284163	hypothetical protein EUBIFOR_02562 [Eubacterium biforme DSM 3989]
gi|15614407	3-oxoacyl-[acyl-carrier-protein] synthase III [Bacillus halodurans C-125]
gi|16799344	2-C-methyl-D-erythritol 4-phosphate cytidylyltransferase [Listeria innocua Clip11262]

Probability Based Mowse Score

Ions score is -10*Log(P), where P is the probability that the observed match is a random event.
Individual ions scores > 52 indicate identity or extensive homology (p<0.05).
Protein scores are derived from ions scores as a non-probabilistic basis for ranking protein hits.

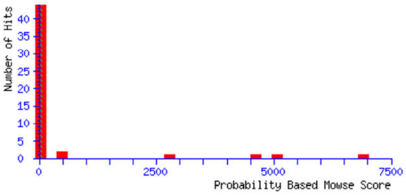

Peptide Summary Report

Format As | Peptide Summary | Help

Significance threshold p< 0.05 | Max. number of hits AUTO

Standard scoring ○ MudPIT scoring ◉ | Ions score or expect cut-off 0 | Show sub-sets 0

Show pop-ups ◉ Suppress pop-ups ○ | Sort unassigned Decreasing Score | Require bold red ☐

(6-3) Peptide Summary Report (protein spot 6, IE-6) Other Firmicutes

Mascot Search Results

```
User          : liaopc
Email         :
Search title  : IE-6
MS data file  : merge.txt
Database      : NCBInr 090616 (9069431 sequences; 3106408609 residues)
Taxonomy      : Other Firmicutes (967613 sequences)
Timestamp     : 11 Jan 2012 at 03:19:47 GMT
Protein hits  : gi|116629131 fructose/tagatose bisphosphate aldolase [Lactobacillus gasseri ATCC 33323]
                gi|42518609  fructose-bisphosphate aldolase [Lactobacillus johnsonii NCC 533]
                gi|227360402 fructose-bisphosphate aldolase [Lactobacillus sakei subsp. carnosus DSM 15831]
                gi|227876934 fructose-bisphosphate aldolase [Lactobacillus crispatus JV-V01]
                gi|164687868 hypothetical protein CLOBAR_01512 [Clostridium bartlettii DSM 16795]
                gi|229135967 transposase, IS605 [Bacillus cereus BDRD-ST196]
                gi|197301564 hypothetical protein RUMLAC_00290 [Ruminococcus lactaris ATCC 29176]
                gi|30262473  hypothetical protein BA2474 [Bacillus anthracis str. Ames]
                gi|158321234 adenosylcobinamide-phosphate guanylyltransferase [Alkaliphilus oremlandii OhILAs]
                gi|81429140  fructose-bisphosphate aldolase [Lactobacillus sakei subsp. sakei 23K]
                gi|169191287 UDP-N-acetylglucosamine 1-carboxyvinyltransferase [Paenibacillus sp. JDR-2]
                gi|47567502  ABC transporter, ATP-binding protein [Bacillus cereus G9241]
                gi|153813549 hypothetical protein RUMOBE_03971 [Ruminococcus obeum ATCC 29174]
                gi|1706361   RecName: Full=Transcriptional regulatory protein degU
                gi|168333288 hypothetical protein Epulo_00390 [Epulopiscium sp. 'N.t. morphotype B']
                gi|228948651 Cell surface protein [Bacillus thuringiensis serovar monterrey BGSC 4AJ1]
                gi|172058143 glutamate-1-semialdehyde-2,1-aminomutase [Exiguobacterium sibiricum 255-15]
                gi|145952657 hypothetical protein CdifQ_04004036 [Clostridium difficile QCD-32g58]
                gi|229229306 ATPase component of various ABC-type transport systems with duplicated ATPase domain [Desulfotomaculum a
                gi|229092047 beta-lactamase class C [Bacillus cereus Rock3-42]
                gi|228583035 trigger factor protein [Clostridium sp. 7_2_43FAA]
                gi|229106627 hypothetical protein bcere0019_54130 [Bacillus cereus Rock3-28]
                gi|187935417 serine protein kinase [Clostridium botulinum B str. Eklund 17B]
                gi|229172724 Fis-type helix-turn-helix domain protein [Bacillus cereus MM3]
                gi|152974405 sortase family protein [Bacillus cereus subsp. cytotoxis NVH 391-98]
                gi|238916527 basic amino acid/polyamine antiporter, APA family [Eubacterium eligens ATCC 27750]
                gi|167766734 hypothetical protein CLOSS21_01240 [Clostridium sp. SS2/1]
                gi|150391186 YheD [Alkaliphilus metalliredigens QYMF]
                gi|222529355 von Willebrand factor type A [Anaerocellum thermophilum DSM 6725]
                gi|192808568 hypothetical protein GYMC10DRAFT_1077 [Geobacillus sp. Y412MC10]
                gi|212640452 S-layer glycoprotein protein, contains three N-terminal SLH domains [Anoxybacillus flavithermus WK1]
                gi|52078774  putative lipoprotein [Bacillus licheniformis ATCC 14580]
                gi|229541561 two component transcriptional regulator, LuxR family [Bacillus coagulans 36D1]
                gi|194016979 YpiF [Bacillus pumilus ATCC 7061]
                gi|160939974 hypothetical protein CLOBOL_04864 [Clostridium bolteae ATCC BAA-613]
                gi|229051384 hypothetical protein bcere0027_53120 [Bacillus cereus AH676]
                gi|218284163 hypothetical protein EUBIFOR_02562 [Eubacterium biforme DSM 3989]
                gi|15614407  3-oxoacyl-[acyl-carrier-protein] synthase III [Bacillus halodurans C-125]
                gi|16799344  2-C-methyl-D-erythritol 4-phosphate cytidylyltransferase [Listeria innocua Clip11262]
```

Probability Based Mowse Score

Ions score is -10*Log(P), where P is the probability that the observed match is a random event.
Individual ions scores > 52 indicate identity or extensive homology (p<0.05).
Protein scores are derived from ions scores as a non-probabilistic basis for ranking protein hits.

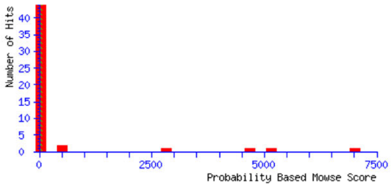

Peptide Summary Report

Format As | Peptide Summary | Help

Significance threshold p< 0.05 | Max. number of hits AUTO

Standard scoring ○ MudPIT scoring ◉ | Ions score or expect cut-off 0 | Show sub-sets 0

Show pop-ups ◉ Suppress pop-ups ○ | Sort unassigned Decreasing Score | Require bold red ☐

If readers are interested in further analysis of this proteomic data, we can make it available upon reasonable request.
